# Repositioning of a Diaminothiazole Series Confirmed to Target the Cyclin-Dependent Kinase CRK12 for Use in the Treatment of African Animal Trypanosomiasis

**DOI:** 10.1021/acs.jmedchem.1c02104

**Published:** 2022-03-18

**Authors:** Alasdair Smith, Richard J. Wall, Stephen Patterson, Tim Rowan, Eva Rico Vidal, Laste Stojanovski, Margaret Huggett, Shahienaz E. Hampton, Michael G. Thomas, Victoriano Corpas Lopez, Kirsten Gillingwater, Jeff Duke, Grant Napier, Rose Peter, Hervé S. Vitouley, Justin R. Harrison, Rachel Milne, Laura Jeacock, Nicola Baker, Susan H. Davis, Frederick Simeons, Jennifer Riley, David Horn, Reto Brun, Fabio Zuccotto, Michael J Witty, Susan Wyllie, Kevin D. Read, Ian H. Gilbert

**Affiliations:** † Drug Discovery Unit, Wellcome Centre for Anti-Infectives Research, Division of Biological Chemistry and Drug Discovery, University of Dundee, Dow Street, Dundee DD1 5EH, United Kingdom; ‡ Wellcome Centre for Anti-Infectives Research, Division of Biological Chemistry and Drug Discovery, School of Life Sciences, University of Dundee, Dow Street, Dundee DD1 5EH, United Kingdom; § GALVmed, Doherty Building, Pentlands Science Park, Bush Loan, Penicuik, Edinburgh EH26 0PZ, United Kingdom; ∥ Swiss Tropical and Public Health Institute, Socinstrasse 57, CH-4002 Basel, Switzerland; ⊥ University of Basel, Petersplatz 1, CH-4001 Basel, Switzerland; # University of Greenwich, Medway Campus, Central Avenue, Chatham Maritime, Chatham, Kent ME4 4TB United Kingdom; ∇ Centre International de Recherche-Développement sur l’Elevage en zone Subhumide (CIRDES), No 559 ru 5-31 angle Av. du Gouverneur Louveau, 01 BP: 454 Bobo-Dioulasso 01, Burkina Faso

## Abstract

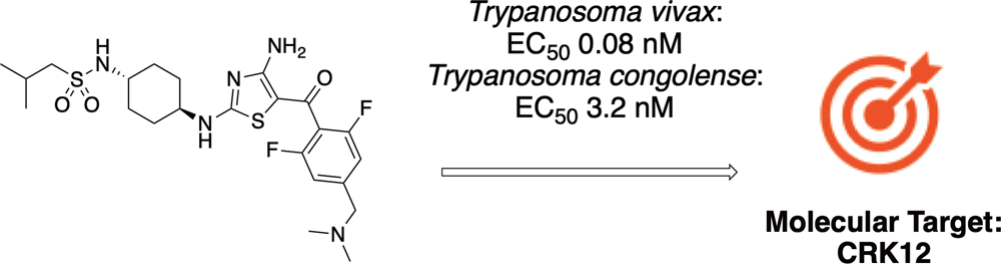

African animal trypanosomiasis or nagana, caused principally by infection of the protozoan parasites *Trypanosoma congolense* and *Trypanosoma vivax,* is a major problem in cattle and other livestocks in sub-Saharan Africa. Current treatments are threatened by the emergence of drug resistance and there is an urgent need for new, effective drugs. Here, we report the repositioning of a compound series initially developed for the treatment of human African trypanosomiasis. A medicinal chemistry program, focused on deriving more soluble analogues, led to development of a lead compound capable of curing cattle infected with both *T. congolense* and *T. vivax* via intravenous dosing. Further optimization has the potential to yield a single-dose intramuscular treatment for this disease. Comprehensive mode of action studies revealed that the molecular target of this promising compound and related analogues is the cyclin-dependent kinase CRK12.

## Introduction

African animal trypanosomiasis (AAT) or nagana is caused by a variety of trypanosome species, principally *Trypanosoma congolense*, *Trypanosoma vivax*, and *Trypanosoma bruceibrucei*, although other species can also cause the disease. Nagana is endemic in at least 37 countries in sub-Saharan Africa,^1^ but it is also present in other regions of the world.^2^ Animal trypanosomes affecting livestock have represented a major constraint to agricultural development in Africa for centuries, and their negative economic impact is increasing in South America and Asia. Chemotherapy and chemoprophylaxis based on diminazene aceturate and isometamidium chloride represent the main means of control. However, there has been little investment in the development of new trypanocides, which has remained inadequate for decades, leading to a situation where the few treatment options available are losing efficacy due to the emergence of drug-resistant parasites. In Africa, the parasite infection is spread through the bite of the tsetse fly. The disease predominantly affects domesticated livestock animals such as cattle and goats, where it causes a range of symptoms in infected animals, including anemia, weight loss, and death. AAT has an enormous economic impact on the agricultural industry in many parts of Africa, resulting in estimated losses of over $600 M annually (www.galvmed.org). Current treatments are at risk from the emergence of acquired drug resistance;^2^ thus, there is an urgent need for the development of new treatments. In the absence of a viable vaccine, improved therapeutics for AAT could have a transformative economic impact in some of the poorest regions of the developing world.

A suitable target product profile (TPP) for a compound to treat AAT is essential to guide the drug discovery process. In terms of drug discovery, there are some particularly challenging issues related to the TPP for this disease:

i. A single-dose treatment is needed, since cattle often roam around the bush and cattle handling facilities are poor, making repeated dosing of animals challenging. Existing chemotherapeutics are single-dose products. To achieve a cure following single dosing, the drug in question needs to be potent, have a suitable mode of action, and have appropriate pharmacokinetic properties. It should distribute widely around the body, particularly to locations where parasites reside. The drug should remain at these locations at trypanocidal concentrations and for a sufficient time to kill all parasites including, importantly, those resistant to current therapies. It is critically important that activity is established against the target pathogens in the host species rather than against model organisms such as *T. brucei* in mouse models of infections.
ii. The drug must be rapidly cleared from treated animals to avoid drug residues in meat and milk.
iii. Treatment should be injectable, ideally intramuscular (IM) for ease of administration but potentially subcutaneous (SC). Ideally this should be done with a single injection due to the difficulty of retaining cattle normally roaming in the wild. Sufficient compound should be administered to give compound levels above the parasiticidal concentration for sufficient time to clear all the parasites. There is a limit to the volume of an individual IM injection, to avoid pain to the cattle due to excess administration of fluid (<5 mL/100 kg). Given the size of cattle (∼250–500 kg), this requires a compound with significant potency, a fast rate of kill, high solubility, together with good distribution to the site of parasite infection, and a reasonably long half-life (but not too long to avoid risk of residues in meat and milk).
iv. The formulation should be easily prepared in a sterile form (or be pre-made) and should be safe for the person administering.
v. The cost of goods should be low, since most of the potential customers are likely to be subsistence farmers.
vi. There should be no requirement for a cold chain for distribution of the drug. Therefore, the compound must be chemically stable under hot and humid conditions for at least 2 years.

The parasites that cause AAT are closely related to those responsible for human African trypanosomiasis (HAT, causative agents: *T.b. rhodesiense* and *T.b. gambiense*). HAT has been the focus of a number of drug discovery programs around the world, and many of the compounds developed in these studies have been assessed for their utility in the animal health arena. As a result of our extensive HAT drug discovery program, several series were developed that demonstrated potent activity *in vitro* and also in our HAT mouse model but were not CNS penetrant. CNS penetration is now considered a requirement for therapeutics targeting HAT; however, this is not the case for AAT. Here, we describe the repurposing and development of a non-CNS penetrant diaminothiazole series, initially developed for HAT, for use in the treatment of AAT. These studies highlight the unique challenges that accompany the development of therapeutics for veterinary use. We also report our comprehensive mode of action studies, revealing cyclin-dependent kinase 12 (CRK12) as the principal molecular target of this promising diaminothiazole series.

## Results

The starting point for our diaminothiazole series was a program aimed at targeting glycogen synthase kinase-3 (GSK3) in *T. brucei*,^3^ as part of the program to tackle HAT. As the series was optimized, it became clear that anti-parasitic activity was not driven by inhibition of *Tb*GSK3 but through an alternative mechanism of action evidenced by a divergence between enzyme inhibition and phenotypic activity. Some compounds from this series were extremely potent against the bloodstream form of *T.b. bruceiin vitro.* Compound **1** was selected as a starting point for the AAT program as it was extremely potent against *T.b. brucei* and in animal models of *T.b. brucei* infection and was our most characterized compound (Table 1). It was also found to be a potent inhibitor of *T. congolensein vitro* and marginally less active against *T. vivax* (Table 1). Importantly, this compound was cytocidal, since our experience indicates that cidality is essential for *in vivo* activity.^4,5^ While our initial entry point was activity against *T.b. brucei*, our ultimate focus was *T. congolense* and *T. vivax.*

**Table 1:**
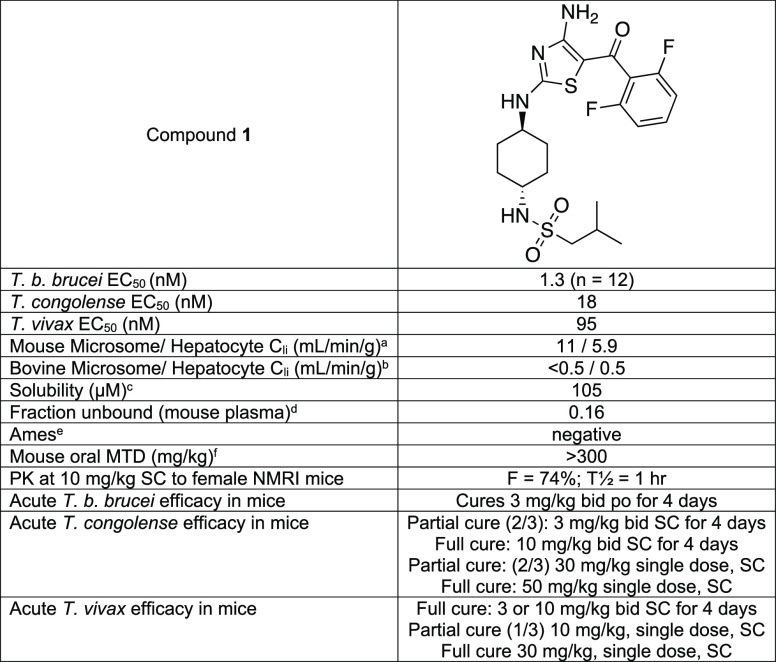
Profile of Compound **1**

a Intrinsic clearance in mouse microsomes and hepatocytes.

b Intrinsic clearance in bovine liver microsomes and hepatocytes.

c Aqueous solubility.

d Fraction of the compound unbound in mouse plasma.

e The Ames test for potential genotoxicity.^6^

f The maximum tolerated dose when given orally.

Based on promising *in vitro* data, a pharmacokinetic study was initiated with compound **1** in NMRI mice. Following a single (SC) administration at 10 mg/kg, bioavailability of compound **1** in female NMRI mice was determined to be 74% with a half-life of ∼1 h (Table 1 and Figure S1). With good SC pharmacokinetics, an efficacy study was initiated in mouse models of *T. congolense* and *T. vivax* to give an early indication of the feasibility of proceeding to cattle. Against *T. congolense* infected mice, a single SC dose of compound **1** at 50 mg/kg or dosing at 10 mg/kg once daily for 4 days elicited sterile cure. Against *T. vivax*, compound **1** was marginally more efficacious *in vivo* with a single SC dose at 30 mg/kg or dosing at 3 mg/kg once daily for 4 days eliciting sterile cure. Please see the Supporting Information (Sections 2.5, 3.1, and 3.2) for more details of our mice models.

### Pharmacokinetic and Cattle Efficacy Proof of Concept Studies

Given promising *in vivo* efficacy data in mice (Table 1), compound **1** was progressed into cattle pharmacokinetic studies, dosing both IV and IM (Table 2, Figure S2, and Table S4). The volume of distribution indicated that the compound penetrated tissues beyond the vasculature. However, it had a moderate clearance from the blood and a relatively short half-life (∼1.8 h)*. In vitro* experiments with microsomes and hepatocytes (Table 1) indicated that the compound was very stable to metabolism in the liver, suggesting that non-hepatic clearance mechanisms may also play a role in the total blood clearance, although the precise cause is not known. Following IM administration, compound **1** was found to have low bioavailability (IM 9%; Table 2). This low bioavailability was likely a consequence of poor compound release from the injection site following IM administration in cattle due to the low solubility of compound **1**, probably leading to compound accumulation and precipitation at the injection site. However, considering that the exposure at mouse efficacious doses suggested that it was only necessary to maintain free blood levels above the estimated EC_99_ (1.6 ng/mL) for approximately 16 h to deliver sterile cure, compound **1** continued its progression into preliminary cattle efficacy, based on the assumption that similar exposure levels were required in cattle to mice to obtain efficacy. Our previous experience indicates that free blood levels need to be maintained above EC_99_ to deliver efficacy, although this is dependent on the mechanism of action, which in the case of HAT must be cytocidal.^7^

**Table 2:** Pharmacokinetic Parameters of Compounds **1** and **2** Following IV or IM of Free Base to Male Fresian-Holstein Cattle^a^

compound	**1**	**2**
IV dose	2.5 mg/kg; *n* = 4	2 mg/kg; *n* = 3
*C*_lb_ (mL/min/kg)	20 ± 4	15 ± 5
*V*_dss_ (L/kg)	2.4 ± 0.8	2.3 ± 0.6
half-life (h)	1.8 ± 0.5	4 ± 2
AUC _0-∞_ (ng-min/mL)	130,000 ± 30,000	140,000 ± 40,000
IM dose	5 mg/kg; *n* = 4	10 mg/kg; *n* = 3
*C*_max_ (ng/mL)	130 ± 60	1700 ± 800
*T*_max_ (h)	0.44 ± 0.13	1.3 ± 0.6
AUC _0-∞_ (ng-min/mL)	21,000 ± 5000	500,000 ± 30,000
*F* (%)	8.8 ± 2.1	73 ± 6

a Data are mean ± SD; for compound **1**, formulation IV was 2.5% NMP, 30% PEG400, 37.5% propylene glycol, and 30% water by volume; formulation IM was 10% NMP, 40% 2-pyrrolidone, 30% PEG400, and 20% water by volume; for compound **2**, formulation was 40% 0.3 M acetate buffer, 20% Kolliphor EL, 20% glycerol formal, and 20% 2-pyrrolidone.

Following SC administration of compound **1** to calves infected with *T. congolense* at 5 mg/kg given twice at 12 h intervals, parasitaemia was suppressed below the level of detection. However, following the cessation of treatment, parasitaemia quickly re-emerged to such an extent that after 4 days, all calves were positive. It should be noted that in this study, calves were infected with a highly virulent *T. congolense* isolate known to be resistant to established anti-trypanosomal agents diminazene and isometamidium. Testing compounds in this model represents a rigorous test of efficacy; thus, the observed reduction in parasite numbers in calves dosed with compound **1** was very encouraging.

These findings suggested that efficacy could be improved if a formulation could be found to increase levels of compound release following IM injection without accompanying precipitation. Indeed, considering the potency of compound **1**, and the mouse PK/PD data, a fivefold or greater improvement in distribution from the injection site in calves could be sufficient to deliver sterile cure. Unfortunately, investigation of several different formulations failed to improve the pharmacokinetic profile (for example, see Figure S3). These studies were carried out in Sprague Dawley rats, which we showed was a good model to predict of the IM profiles in cattle (Figure S4).

### Compound Optimization

**Scheme 1:**
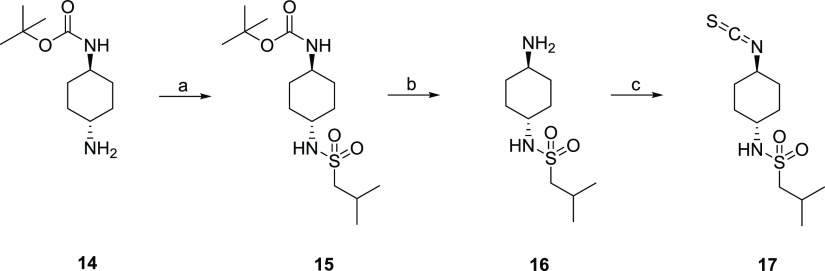
Synthesis of the Isothiocyanate Building Block **17** Reagents and conditions: (a) *n*-BuLi, isobutylsulfonyl chloride, THF, −78 °C → R.T., 12 h; (b) HCl, dioxane/CH_2_Cl_2_, 0 °C → R.T., 24 h; (c) 1,1′-thiocarbonyldiimidazole, CH_2_Cl_2_, R.T., 16 h.

**Scheme 2:**
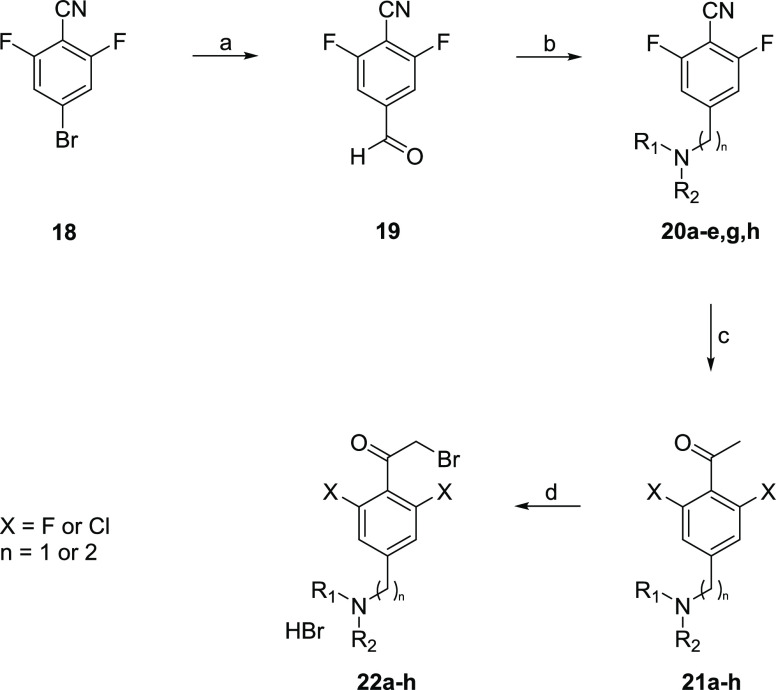
Generic Synthetic Route to Key α-bromoketone Building Blocks Used in the Synthesis of Anti-Trypanosomal Aminothiazoles Reagents and conditions: (a) *i*-PrMgCl, formyl piperidine, THF, 0 °C, 2 h; (b) amine, NaBH(OAc)_3_, CH_2_Cl_2_, R.T. 16 h; (c) MeMgBr, PhMe, 110 °C, 2.5 h; (d) Br_2_, HBr, AcOH, 60 °C, 2 h. Intermediates **20g** (*n* = 2) and **21f** (X = Cl) were prepared individually, not from **19** and **20**, respectively.

**Scheme 3:**
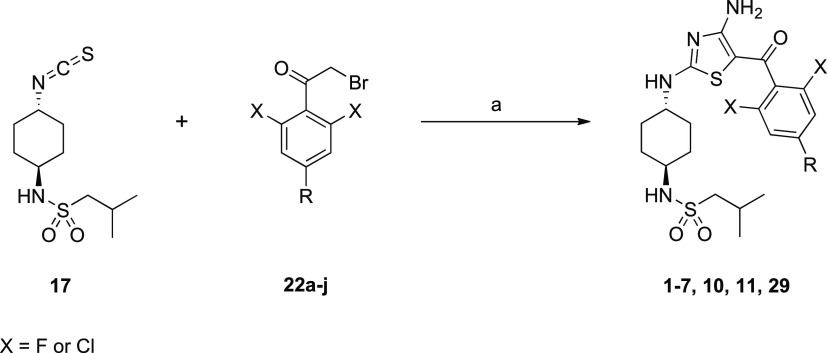
Generic Synthesis of Anti-Trypanosomal Amino-Thiazoles from Isothiocyanate **17** and Substituted α-Bromoketones Reagents and conditions: (a) cyanamide, KO^t^Bu, MeCN/^t^BuOH, R.T., 12 h.

A chemistry program was initiated to develop compounds with enhanced solubility (see Schemes 1–3 in the “Chemistry Synthesis” section below). We were also looking to increase the volume of distribution (*V*_dss_), which should increase half-life and give exposures above EC_99_ for longer times. Of course, it should be noted that this is a delicate balance since increasing volume to increase compound half-life could give rise to unwanted residues in meat or milk for an extended duration.

Previous structure–activity relationship data from our HAT program indicated that the NH_2_ of the amino thiazole, the 2,6-substitution of the phenyl ring, and the *trans* isomer of the cyclohexyl ring were optimal. Significant changes to the sulfonamide group were not tolerated. However, it was possible to introduce a basic substituent in the 4-position of the phenyl group (compounds **2**–**9** and **11**, Table 3), which should increase solubility. Basic compounds also tend to have a higher *V*_dss_ that can lead to a longer half-life. The methylene dimethylamine analogue (**2**) was particularly efficacious in mouse models of infection. Methyl groups attached to nitrogen can be metabolically labile; therefore, a series of analogues were prepared where the methyl groups were tied up. The methylpiperazine (**3**) and morpholine (**4**) analogues were less efficacious in the mouse models. The pyrrolidine (**5**) and azetidine (**6**) analogues lost potency against the parasites *in vitro*. Putting an extra methyl group in the chain (**7**) led to a reduction in activity against *T. congolense.* Removing one of the methyl groups (**8**) led to a compound with a similar profile to dimethyl analogue **2**. This compound (**8**) is a metabolite of compound **2**. Removing both methyl groups (**9**) led to a slight loss in activity against *T. congolense.* Replacing the difluoro group with a dichloro group (**10**, **11**) resulted in compounds that were very potent *in vitro*, with compound **10** also demonstrating impressive potency *in vivo*. Unfortunately, however, compound **10** did not offer a solubility improvement over compound **1**.

**Table 3:**
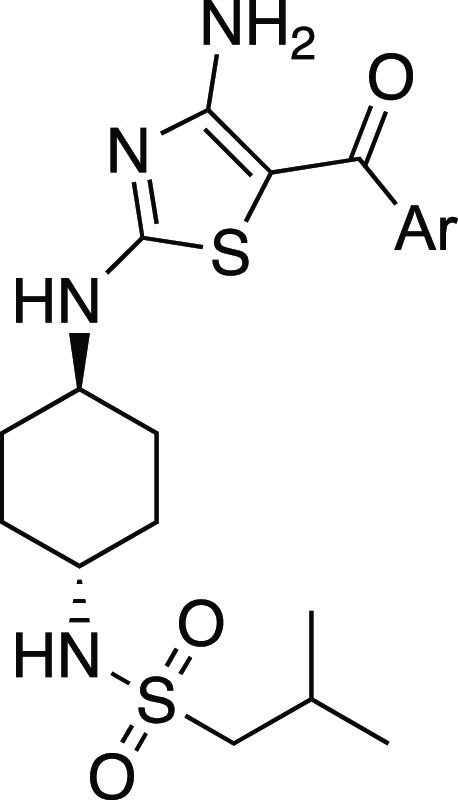

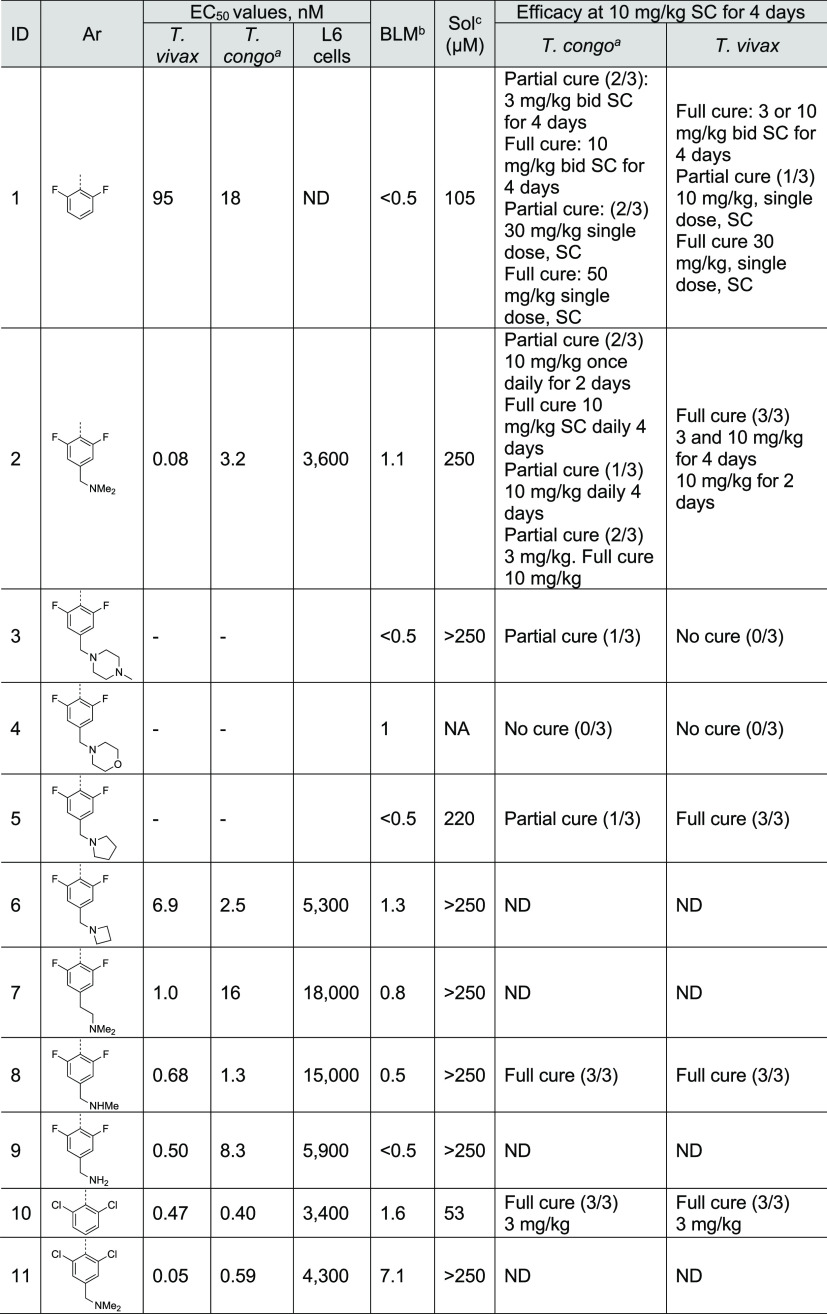
*In Vitro* and *in Vivo* Data for Various Analogues of Compound **1**

a *T. congo*: *T. congolense*.

b BLM: bovine microsomal clearance (mL/min/g).

c Sol: kinetic solubility. For *T. congolense*, *T. vivax* and L6 cells data represent the mean of at least 3 independent experiments each comprised of two technical replicates. ND = not determined.

### Cattle Pharmacokinetics and Efficacy

Our experience indicates that compounds with EC_50_ values <10 nM (ideally ∼1 nM) are more likely to be efficacious via IM injection. From those compounds assessed *in vivo* (Table 3), compound **2** was fully curative against both *T. congolense* and *T. vivax*-infected mice dosed once daily for 4 days at 10 mg/kg and showed promising efficacy at lower doses. The potential metabolite of compound **2** due to demethylation (**8**) retained or even improved potency. The derivative with both methyl groups removed (**9**) also retained some activity, albeit with slightly less activity against *T. congolense*. Therefore compound **2** became our lead compound; its extensive profiling is detailed below.

We estimated that a solubility of 50–100 mg/mL would be required to conduct cattle pharmacokinetic and efficacy studies and for any future veterinary therapy. Following formulation work with compound **2**, the hydrochloride salt in 40% 0.3 M acetate buffer/20% Kolliphor EL/20% glycerol formal/20% 2-pyrrolidone delivered the best pharmacokinetic profile (see Figure S5A) when dosed IM to rat at a dose level of 10 mg free base/kg (50 mg/mL). The pharmacokinetic IM properties of compound **2** with this improved formulation were such that delivery of an efficacious dose was considered possible based on a comparison with the pharmacokinetic profile required for full efficacy in mouse (Figure S5B).

Following IM administration to cattle, compound **2** was found to have markedly improved bioavailability (73%), giving a much larger exposure. Interestingly, despite being a basic amine, compound **2** had a similar *V*_dss_ to compound **1** but had a slightly lower clearance in blood than compound **1**, as well as a longer half-life. Based on the comparison with the blood concentration–time profile necessary to deliver full cure in mice (Figure S5B), there was potential that the blood concentration–time profile in cattle following IM administration of compound **2** at 10 mg/kg could deliver cure (Figure S6).

**Figure 1:**
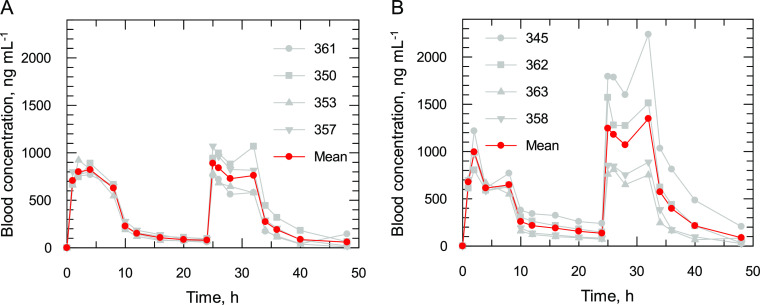
Blood concentration–time profiles of compound **2** in Friesian-Holstein cattle following an IV bolus and infusion protocol to animals infected with (A) *T. congolense* or (B) *T. vivax*. The mean concentration–time profile is highlighted in red. The vehicle was 5% glucose. This work was carried out at Clinvet.

Unfortunately, an injection site tolerability issue arose during the pharmacokinetic study in cattle that was not evident in rats. The effects, irritancy and necrosis, were of sufficient severity to prevent progression to efficacy in cattle using IM delivery without further investigation into the cause of the tolerability issue. To circumvent these issues, compound **2** was assessed in an *in vivo* proof of concept efficacy study in Friesian-Holstein cattle using an IV infusion regimen. The study was conducted in four animals infected with *T. congolense* (KONT2/133) and four animals infected with *T. vivax* (STIB719). Three additional animals infected with each strain formed the vehicle control groups and were dosed IV once daily with saline. Pharmacokinetic modeling was used to define a dosing regimen that mimics the blood concentration–time profile that delivered cure in mice (10 mg/kg SC once daily for 2 days, with a *C*_trough_ of >20 ng/mL (>38 nM)). A continuous infusion over 36 h and two loading doses 24 h apart were required. The exact regimen was a loading IV bolus dose of 2 mg/kg at time 0 and 24 h with continuous infusions of 0.3 mg/kg/h (0–8 h and 24–32 h) and 0.03 mg/kg/h (8–24 h and 32–36 h) (Figure 1). All cattle infected with either *T. vivax* or *T. congolense* and treated with compound **2** were cured of infection at 100 days using this regimen. With proof of concept achieved, further investment of time to address the IM tolerability issues associated with compound **2** was now warranted.

### Formulation and Alternative Salt Studies

In an attempt to circumvent injection site tolerability issues and improve solubility to support IM dosing, the salt was varied, and the formulation changed (Supporting Information, Section 2.8, Table S6, and Figure S7). During the study, all calves remained clinically well with only minor local injection site reactions. We also examined the feasibility of SC administration in cattle, but blood exposure was considerably lower than following IM administration (Table S7). As a result of these studies, the free base was progressed using a formulation of Kolliphor EL, *N*-methyl pyrrolidone, 2-pyrrolidone, and 11% water using IM administration, which appeared to solve the problem.

In parallel, all the formulations were assessed in rats (IM), and the results were comparable to those in cattle, observationally and with regard to creatine kinase levels. These observations offer confidence that rats can be used as a model animal in the drug development path going forward prior to studies in cattle. Exposure following IM injection in rats was sufficient to warrant progression to cattle efficacy studies, although the short half-life raised concerns that the required time above the EC_99_ blood concentration may not be sufficient for cure following a single IM dose at 10 mg/kg. However, studies in *T. congolense*-infected mice, using implanted osmotic pumps (ALZET; Figure S8, full cure with a blood total *C*_max_ at 34–65 ng/mL) and microcalorimetry (compound **2** slowed parasite growth at 10 ng/mL and delivered rapid kill at 30 and 100 ng/mL), provided confidence that IM efficacy in cattle remained feasible following two IM doses at 10 mg/kg.

### Cattle Proof of Concept IM Study

Compound **2** was progressed into IM efficacy studies dosing at 10 mg/kg either once or twice, 15 h apart, in cattle infected with *T. congolense* or *T. vivax*. Dosing twice at 10 mg/kg 15 h apart cured three out of the eight cattle infected with *T. congolense* (treatment groups T2 and T4), while none of the *T. vivax-*infected cattle were cured*.* The study design and efficacy outcome are summarized in Table 4 (Tables S8 and S9).

**Table 4:** Cattle Efficacy for Compound **2**^b^

treatment group	compound	parasite species	dosage (mg/kg)	regimen	no. of animals	efficacy
T1	**2**	*T. congolense*	10	once	6	0/6
T2	**2**	*T. congolense*	10	twice, 15 h apart	4	1/4
T3	saline	*T. congolense*	0.2 mL/kg	twice, 15 h apart	2	0/2
T4	**2**	*T. congolense*	10	saline twice, followed by compound **2** rescue dose twice IM 5 days later	4	2/4
T5	**2**	*T. vivax*	10	once	6	0/5^a^
T6	**2**	*T. vivax*	10	twice, 15 h apart	4	0/3^a^
T7	saline	*T. vivax*	0.2 mL/kg	twice, 15 h apart	2	0/2
T8	**2**	*T. vivax*	10	saline twice, followed by compound **2** rescue dose once IM 4 days later	1	ND
T9	**2**	*T. vivax*	10	saline twice, followed by compound **2** rescue dose twice IM 4 days later	3	0/2^a^

a Efficacy was not determined for one of the animals.

b All treatments were administered to female Fulani Zebu cattle via IM injection using Kolliphor EL, *N*-methyl pyrrolidone, 2-pyrrolidone, and 11% water as the formulation using the free base; all animals in the study were monitored for 100 days following treatment.

Even though this formulation was well tolerated when administered to European cattle, when injected into African cattle, it again demonstrated injection site tolerance issues. Compound **2** was only partially curative for cattle infected with *T. congolense* following two doses (1/4 and 2/4 in different treatment arms), administered 15 h apart (Table 4, Table S8, and Figure S9a). The curative regimen aligned well with PK/PD data from mice. However, cattle infected with *T. vivax* and dosed IM were not cured despite the fact that PK data from some *T. vivax*-infected cattle (Table 4, Table S9, and Figure S9b) predicted that a dosing at this level should have been curative based on mouse data. This lack of efficacy at exposures expected to deliver a cure is even more perplexing when the metabolism of compound **2** is considered. This compound is metabolized rapidly to an equally potent metabolite (compound **8**). The rate and extent of this metabolism is higher in *T. vivax*-infected animals compared to those infected with *T. congolense* (Table S10) for reasons that are not clear.

### Mode of Action Studies

Understanding of the mode of action may help facilitate future development of this series. Our experience of drug target deconvolution in kinetoplastids has demonstrated that no one methodology is sufficient to determine the mechanism of action (MoA) of all compounds. With this in mind, we have developed an integrated approach to MoA studies using a range of orthogonal methodologies. The value of our orthogonal strategy is that multiple, unbiased approaches can be used in concert to identify and validate the molecular targets of phenotypically active compounds. Here, we describe the various approaches that were employed to determine the molecular target of compound **2** and related compounds from this series.

### Genome-Wide RNAi Screens

**Figure 2:**
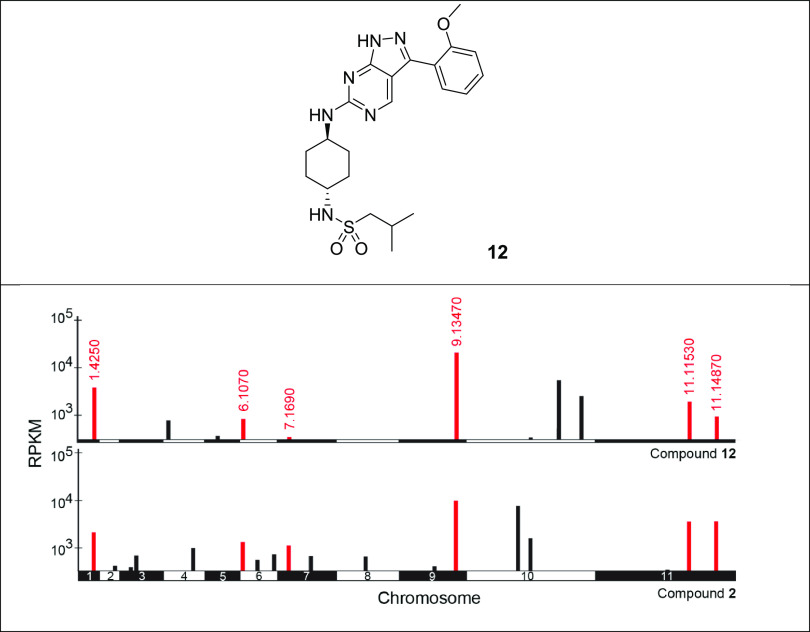
RNAi screens in *T. brucei*. Compounds **2** and **12** were screened against a genome-wide RNAi library in *T. brucei*. Genome-wide maps showing RIT-seq hits from the screening of compounds **2** and **12**. Multiple hits are shared by the two screens (red) with *Tb* gene IDs annotated. RPKM, reads per kilobase of transcript per million mapped reads.

**Figure 3:**
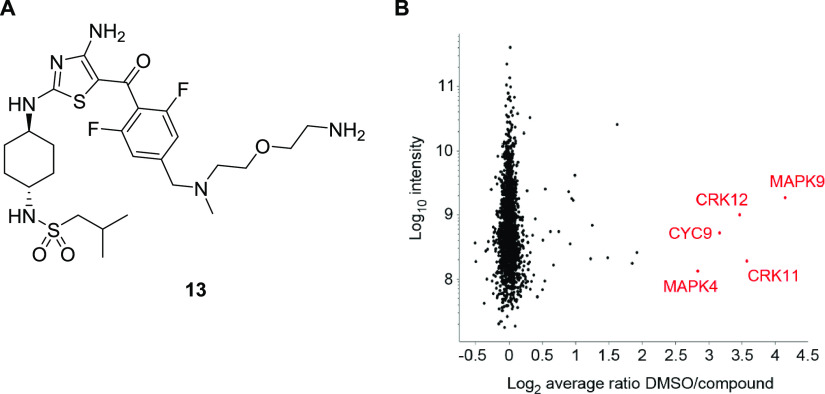
Identification of proteins specifically binding to compound **13**. Stable isotope-labeled cell lysate from *T. brucei* procyclics was incubated in the presence of either compound **2** or DMSO. Lysates were then incubated with (A) probe **13**, an active derivative of compound **2**, bound to a resin via a polyethyleneglycol (PEG) linker. (B) Resin-bound proteins were analyzed by LS-MS/MS with a high DMSO/compound ratio, indicating specific binding to probe **13**. Red annotations: Log_2_ DMSO/compound ratio >2.5, a 5.7-fold enrichment. Proteins shown were identified by the detection of at least two unique peptides and two ratio H/L counts. Data is the mean of three experiments.

Our previous studies have demonstrated that genome-wide RNA interference (RNAi) screens can be effective in identifying drug resistance determinants in *T. brucei* and assisting in the determination of drug MoA. Lethal concentrations of compound **2** and a compound from a closely related series (compound **12**) were used to screen a genome-scale *T. brucei* bloodstream-form RNAi library, and drug-resistant populations were then subjected to RNAi target sequencing (RIT-seq).^8,9^ During screening under tetracycline induction, each trypanosome produces double-stranded RNA (dsRNA) from an integrated RNAi target fragment, and the resulting target knockdown has the potential to confer a growth advantage under drug selection. RIT-seq then generates a readout that identifies the RNAi target fragments responsible (Figure 2). High-throughput sequencing of the selected populations following screening with compound **2** identified several proteins including a histone acetyltransferase (HAT2, 11.11530),^10^ a kinetochore protein and putative acetyltransferase (KKT23, 10.6600), a putative SET-domain methyltransferase (9.13470), and a putative CW-type zinc finder involved in DNA-binding and/or protein–protein interactions (10.11720) (Tables S14 and S15). All the proteins are predicted to be nuclear except 11.14870.^11^ The RIT-seq profiles of compound **2** shared significant similarities to those identified in screens with compound **12** (Figure 3), a compound that was developed in a program for visceral leishmaniasis following a scaffold hop from this series.^12,13^ Six out of the ten top “hits” identified in screens with the diaminothiazole compound **2** were also identified in screening with compound **12**. In previous studies, we established that the pyrazolopyrimidine compound **12** specifically targets cyclin-dependent kinase 12 (CRK12) in *L. donovani*, raising the possibility that compound **2** may also target this kinase in *T. brucei*.

### SILAC-Enabled Pull-Down Studies

**Scheme 4:**
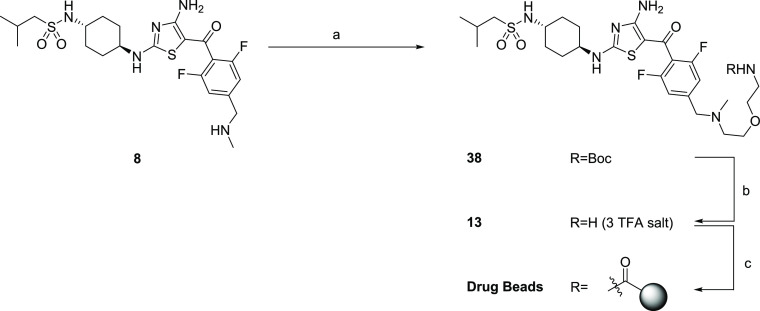
Synthesis of Aminothiazole Drug Beads for Pull-Down Chemical Proteomics Studies Reagents and conditions: (a) **37**, K_2_CO_3_, MeCN, 82 °C, 16 h; (b) TFA, CH_2_Cl_2_, 0 °C → R.T., 2.5 h; (c) (i) NHS-activated resin, DIPEA, DMSO, R.T., 24 h and (ii) ethanolamine, DIPEA, DMSO, R.T., 24 h.

Chemical proteomic studies were carried out by immobilizing an analogue of compound **2** (probe **13**; Figure 3A and Scheme 4) to magnetic beads via a polyethyleneglycol (PEG) linker. Appropriate attachment sites for the PEG linker were informed by the established structure–activity relationships within this series, and an advanced intermediate toward the final derivatized compound was screened against both bloodstream and procyclic forms of *T. brucei* to ensure that it retained potency. The analogue was immobilized by attachment to NHS-ester-activated paramagnetic beads. Activated beads were then incubated in SILAC (stable isotope labeling by amino acids in cell culture)-labeled *T. brucei* procyclic whole-cell lysates in the presence or absence of compound **2** (10 μM) with either “light-“ or “heavy”-labeled lysate. After combining the eluates from the beads and performing proteomic analyses, proteins that bound specifically to the diaminothiazole pharmacophore were distinguished from proteins that bound non-specifically to the beads by virtue of compound **2**:DMSO tryptic peptide isotope ratios. As a result of these studies, four kinases and a cyclin were identified as specific binders of the compound **2**-derivatized beads (Figure 3B and Table S11). It should be noted that of the four kinases identified, only CRK12 has been established as essential for survival of *T. brucei*.^14^ In addition, the cyclin identified in this data set (cyclin 9) is the partner cyclin of CRK12.^15^

### Resistant Cell Line Generation Followed by Whole Genome Sequencing

**Figure 4:**
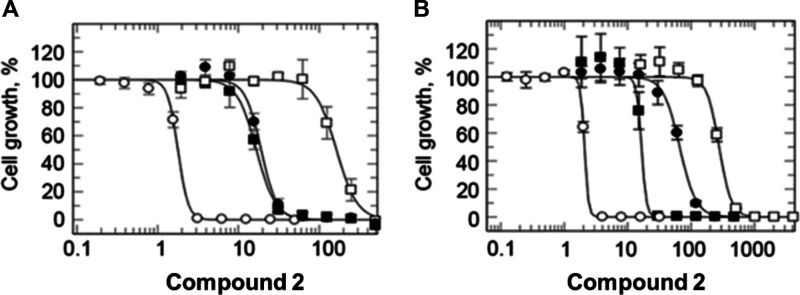
Target identification and validation. (A) Dose–response curves for wild-type (open circles) and resistant lines (R1: closed squares, R2: open squares, and R3: closed circles) treated with compound **2**. (B) Dose–response curves for wild-type (open circles) and CRISPR-Cas9-edited CRK12 with the following changes: Gly492Val (closed squares), Gly492Gln (closed circles), and Leu482Phe (open squares) in the presence of compound **2**.

To gain further insight into the molecular target(s) of this series, resistance was generated against compound **2** in a drug-sensitive clonal line of bloodstream *T. brucei*. Trypanosomes were cultured in the continuous presence of compound **2** for a total of 122 days. Starting at 4 nM (∼3 × EC_50_, Table 1), three independent cultures were exposed to stepwise increasing concentrations of the drug until they were routinely growing in 25 nM compound **2**. Following drug selection, resistant parasites were cloned by limiting dilution. The relative sensitivities of these cloned cell lines were established and compared to that of wild-type parasites. Resistant lines R1 and R3 were 10-fold resistant, while R2 was 90-fold resistant to compound **2** (Figure 4A). Whole genome sequencing analysis (WGS) of all three resistant clones revealed that they shared four single nucleotide polymorphisms compared to parental wild-type (Tables S9 and S10). In addition, all three clones had lost a significant part of chromosome 10 and gained additional copies of a region of chromosome 8. All three resistant lines carried a mutation in Tb927.4.4590, a gene encoding a non-essential putative kinase and in Tb927.11.10300, encoding a hypothetical protein. Of particular note, all three clones (R1–3) shared a non-synonymous substitution (Gly492Ala) in Tb927.11.12310, the gene encoding CRK12. In addition, R2 harbored a second non-synonymous substitution (Leu482Phe) in CRK12 (Table S10).

### Validation of CRK12 as the Target of Compound **2**

Three unbiased and independent approaches converged to identify CRK12 as the likely target of compound **2**. Next, we used CRISPR-Cas9 precision base editing to interrogate the role of CRK12-related mutations in resistance.^16^ A codon-targeted saturation mutagenesis strategy was used to investigate mutations that could support resistance to compound **2** at positions 482 or 492. This was achieved using repair templates randomized (*NNN*) in these specific positions. In position 482, only parasites bearing the same Leu to Phe mutations identified in our drug-selected clones were recovered (*n* = 6), suggesting that alternative mutations did not confer resistance or were not tolerated by trypanosomes. These edited parasites were 133-fold less sensitive to compound **2** compared to wild-type parasites. In contrast, several different amino acids (Ile, Gln, Met, and Val) were tolerated in position 492. Each mutation conferred differing levels of resistance to compound **2**, for instance, parasites bearing a Gly492Val edit were eightfold more tolerant to the compound than wild-type cells, while a Gly492Gln mutation led to 32-fold resistance (Figure 4B); parasites bearing a Gly492Ile or a Gly492Met edit behaved similarly to parasites bearing a Gly492Gln edit. During our CRISPR-Cas9 studies, we did not recover trypanosomes bearing the Gly492Ala mutation identified in our original drug-resistant clones. This is most likely explained by the pronounced growth defect associated with all three of our resistant clones (R1–3) that harbor this specific mutation.

### Molecular Modeling and Docking Studies

**Figure 5:**
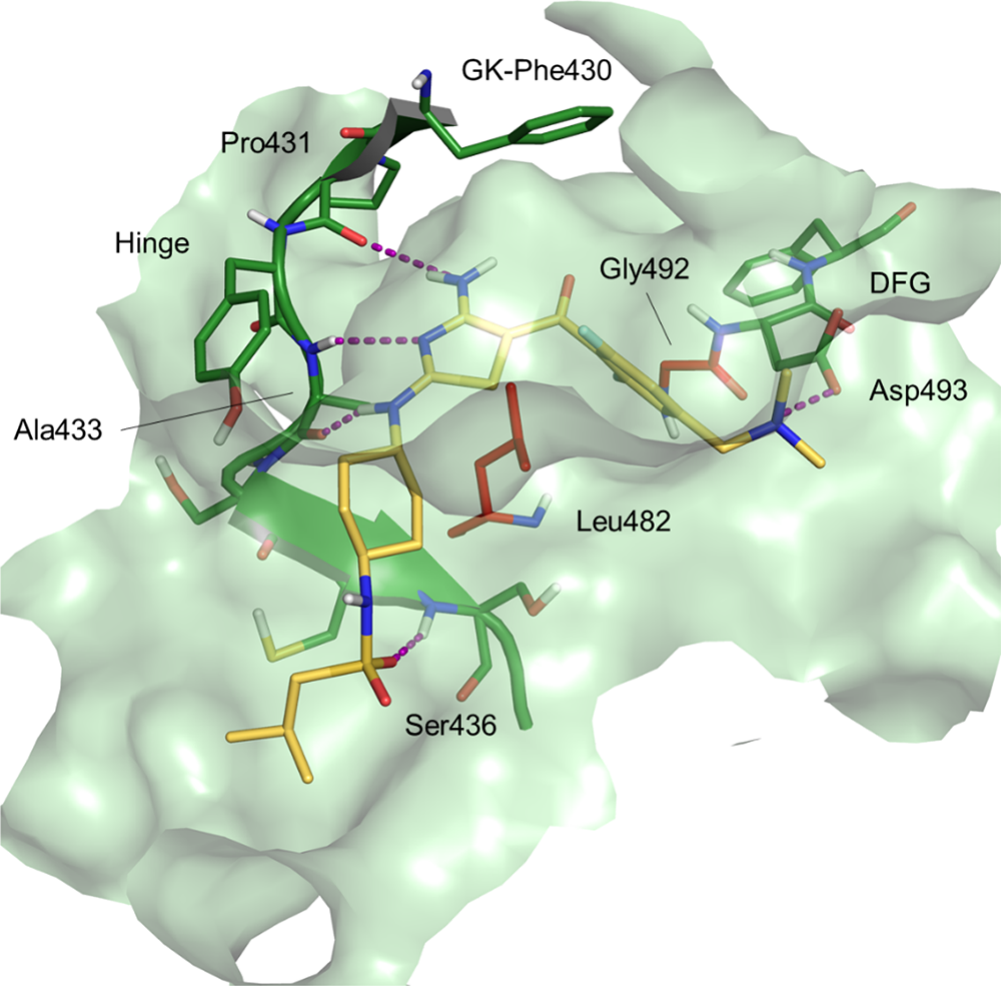
Docking pose for compound **2** in the *Tb*CRK12 homology model. Dotted purple lines represent hydrogen bonds. DFG denotes the conserved Asp-Phe-Gly motif and GK indicates the gate-keeper residue.

To rationalize the role of the mutations identified in our resistant clones, we investigated the binding of compound **2** to CRK12. In the absence of a crystal structure for CRK12 in the kinetoplastids, a homology model was generated using the *human* CDK13 as a structural template. *T. brucei* CRK12 shares 35% sequence identity with this human orthologue (Figure S10). Despite the relatively low level of sequence identity, the high structural conservation amongst the kinase family enables models of sufficient quality to be generated to support ligand binding studies. This homology model was used for molecular docking studies to investigate the binding of compound **2** in the ATP binding site of CRK12. In the best scoring binding pose, the central 2,4-diaminothiazole moiety of compound **2** binds in the adenine region of the ATP binding site, with the sp^2^ nitrogen of the thiazole ring establishing a critical hydrogen bond with Ala433 backbone NH (Figure 5). The two amino groups in positions 2 and 4 of the thiazole ring act as hydrogen bond donors and interact with the backbone carbonyl oxygens of residues Ala433 and Pro431, respectively. An additional hydrogen bond is formed with the active site, specifically between the oxygen atom of the sulfonamide moiety of compound **2** and the backbone NH of Ser436. The terminal tertiary amine substituent on the phenyl ring of compound **2** is located in an area normally occupied by a magnesium atom that coordinates ATP phosphates and is involved in a charge–charge interaction with Asp493, part of the regulatory DFG motif, common to most kinases.^17^ In addition, the di-fluoro phenyl ring occupies the sugar region below the P-loop of the ATP binding site. The mode of binding obtained by docking is consistent with the SAR generated during the optimization process. It is also consistent with structural information generated for a related diaminothiazole series developed as inhibitors of *human* CDK12.^18^ In these studies, RC-2-73, a compound containing a cyclohexyl substituent on the amino group in position 2 of the thiazole and a −COAr substituent in position 5, similar to compound **2**, was one of the ligands crystallized in the active site of *h*CDK2 (PDB ID 3RKB).^18^

The *Tb*CRK12 homology model allowed us to map the two residues mutated in our compound **2**-resistant clones. Both residues are in the ATP binding site. Gly492 is adjacent to the DFG motif (DFG-1), while Leu482 is in the adenine sub-pocket. Mutation of the DFG-1 residue has been reported previously as a common route to kinase inhibitor resistance. Anaplastic lymphoma kinase (ALK) is a kinase whose dysregulation in humans is associated to numerous oncological diseases including non-small cell lung cancer and anaplastic large cell lymphoma. The DFG-1 mutation G1269A in *h*ALK, equivalent to the G492A mutation in *Tb*CRK12, leads to resistance to Crizotinib,^19^ approved for treatment of non-small cell lung carcinoma. Leu482 is part of the catalytic hydrophobic spine stabilized by the adenine ring.^20^ To maintain the integrity of the hydrophobic spine, Leu482 is highly conserved, and it is present in more than 80% of the kinases in the kinome. In the remaining 20% of kinases, it is replaced with other hydrophobic residues such as Phe or Met.^21^ Thus, the L482F mutation preserves the catalytic function of the kinase and does not compromise ATP binding.

## Chemical Synthesis

The routes to prepare the above compounds are summarized in Schemes 1–4. The cyclohexyldiamine moiety was prepared from *tert*-butyl (*trans*-4-aminocyclohexyl)carbamate (**14**) (Scheme 1). Following sulfonylation (**15**) and removal of the BOC-protecting group (**16**), the free amine was converted to isothiocyanate **17**.

The phenyl moiety was prepared from the appropriate benzonitrile, with the *ortho*-substituents (normally fluorine) in place and a bromine in the 4-position to allow for addition of the methylene amine (Scheme 2). The first step was to formylate at this 4-position. This was achieved by bromine–metal exchange using a Grignard reaction then quenching with formyl piperidine (**19**). The amine could then be introduced by reductive amination (**20**). A Grignard addition to the nitrile gave the ketone **21**, which was then brominated on the α-position using bromine.

Isothiocyanate (**17**) was then condensed with the α-bromoketones **22a**–**j** to give the diaminothiazole core, with the cyclohexyl group and phenyl ketone substituted (**1–7**, **10**, **11**, and **29**) (Scheme 3).

## Discussion

Here, we describe the re-positioning of a compound series that showed potent activity against *T.b. brucei* for AAT. Compounds within this diaminothiazole series were extremely potent against the model organism *T.b. brucei*, and rapidly cytocidal. Importantly, these compounds were confirmed as equally potent against the relevant animal pathogens, *T. congolense* and *T. vivax*, and capable of curing AAT in cattle, caused by either pathogen when given intravenously.

The major challenge we faced was to deliver sufficient compound in a single IM injection to elicit a cure in cattle, in compliance with the current TPP for AAT. Although this was not achieved in this study, we propose that this could be achievable by a combination of one or more of the following: (1) identifying a better formulation to increase the amount of compound delivered to cattle in a single dose, (2) increasing the solubility of the compound, through chemical modification, to facilitate delivery, and (3) increasing the half-life of the compound, potentially achievable by increasing the volume of distribution through chemical modification, although care must be taken to avoid compound residues in meat and milk. One key learning is the very high levels of aqueous solubility required for medicines for cattle efficacy; this is significantly higher than that generally required for human health medicines. Given the tight structure–activity relationships, modification of this diaminothiazole series to address these issues proved to be very challenging, whilst retaining the required potency. A further key learning and one that can be used to refine the drug discovery pathway going forward for AAT, is that rat IM PK proved to be a reliable surrogate of cattle IM PK when exploring different formulations and also for predicting injection site tolerability when equivalent dose volume was applied.

Mouse studies suggest that the potency/efficacy of compound **2** is greater against *T. vivax* than *T. congolense*. The fact that our cattle efficacy studies do not replicate this trend raises broader questions on the legitimacy of the current mouse model as a predictor for efficacy in cattle. *T. vivax* is known to have different tissue tropism compared to *T. congolense.* Further investigation is warranted to determine if tissue trophism played any part in the negative outcome of our studies. In cattle, compound **2** was only curative against *T. congolense* and only when two doses were administered. A more soluble compound with the ability to deliver higher blood levels through higher injection site concentrations or a compound with a longer half-life will be required to achieve cure. With this in mind, progression of compound **2** was discontinued for IM treatment.

We also noted some variability in injection site tolerability across the different breeds of cattle used in this study. This could be due to differences in the nutritional status and/or muscle tone of the cattle, alternatively other factors could be involved. Certainly, this issue merits further investigation and again has potential implications for future AAT-drug discovery programs.

Our comprehensive mode of action studies, using multiple orthoganol approaches, identified the cyclin-dependent kinase CRK12 as the molecular target of this diaminothiazole series. This work again highlights the importance of CRK12 as a viable target for anti-parasitic drug discovery. Indeed, a pyrazolopyrimidine compound (GSK3186899), known to act principally by inhibiting CRK12, has been advanced into clinical trials for use in the treatment of visceral leishmaniasis.^12^ The physiological function and substrate(s) of this kinase in kinetoplastids remain to be determined. However, this information may enable this and related targets to be further exploited for drug discovery.

## Conclusions

In summary, we have identified a compound series that can cure AAT in cattle, when given intravenously. Unfortunately, the compounds within this series are insufficiently soluble to support IM delivery. Nevertheless, this compound series validates CRK12 as a viable and promising drug target for AAT. Either modification of our current series or finding a new series capable of inhibiting CRK12 could yield a compound for treatment of AAT.

## Experimental Section

### Ethical Statements

#### Clinvet

The study protocol was submitted to the Clinvet Animal Ethics Committee (CAEC), and an approval certificate was issued authorizing the research facility to conduct the study. The study protocol was designed to allow the use of the study animals in compliance with the Clinvet Policy on the ethical use of animals using the South African National Standard “SANS 10386:2008 The care and use of animals for scientific purposes” as a reference.

#### Dundee

All regulated procedures on living animals in the Drug Discovery Unit, University of Dundee, were carried out under the authority of a project license issued by the Home Office under the Animals (Scientific Procedures) Act 1986, as amended in 2012 (and in compliance with EU Directive EU/2010/63). License applications will have been approved by the University’s Ethical Review Committee (ERC) before submission to the Home Office. The ERC has a general remit to develop and oversee policy on all aspects of the use of animals on university premises and is a sub-committee of the University Court, its highest governing body.

#### Ridgeway

The study conducted at Ridgeway Research Ltd. was under the authority of a project license issued by the U.K. Home Office under the Animals (Scientific Procedures) Act 1986, as amended in 2012, and in compliance with EU Directive EU/2010/63. The study protocol was approved prior to study commencement by the Ridgeway Animal Welfare and Ethics Review Board (reference RRCA-114-13-07). The protocol was conducted in compliance with the VICH GL9 Good Clinical Practice and EMEA Guidelines for the Conduct of Pharmacokinetic Studies in Target Animal Species (EMEA/CVMP/133/99).

#### Cirdes

The study conducted at Centre International de Recherche–Développement sur l’Elevage en zone Subhumide (CIRDES) was conducted in compliance with applicable regulatory, legal, and ethical requirements, the study protocol, and standard operating procedures. The study protocol was approved prior to commencement of the study by the CIRDES ethical review committee (reference BFA/BOV/15/041) and complied with the requirements of the European Directive 2010/63/EU for protection of animals used for scientific purposes, and with VICH GL9 Good Clinical Practice.

#### NorthWest Biopharm

All procedures during the animal phase of the study were conducted in accordance with the standard operating procedures of NorthWest Biopharm Limited. Data collection and procedures were performed in accordance with the VICH guideline “Good Clinical Practice” (VICH GL9). Animals were housed and maintained according to the requirements of the European Directive 2010/63/EU. The animal facility provided by NorthWest Biopharm Limited is authorized under the establishment authorization number AE19123, by the Health Products Regulatory Authority, which is the body responsible for implementing European Directive 2010/63/EU in Ireland. The study protocol was assessed and authorized by the Health Products Regulatory Authority under the project authorization number AE19123/P005.

### Chemistry

Chemicals and solvents were purchased from Sigma-Aldrich, Alfa Aesar, Apollo Scientific, Fisher Chemicals, TCI U.K., and VWR and were used as received. Air and moisture sensitive reactions were carried out under an inert atmosphere of nitrogen. Analytical thin-layer chromatography (TLC) was performed using pre-coated TLC plates (layer of 0.20 mm silica gel 60 with fluorescent indicator UV254, from Merck). Developed plates were air-dried and analyzed under a UV lamp (UV254/365 nm), and/or with chemical stains where appropriate. Flash column chromatography was performed using prepacked silica gel cartridges (230–400 mesh, 35–70 μm, from Teledyne ISCO) using a Teledyne ISCO CombiFlash Rf. The ^1^H-NMR, ^13^C-NMR, ^19^F-NMR, and 2D-NMR spectra were recorded on a Bruker Avance DPX 500 spectrometer (^1^H at 500.1 MHz, ^13^C at 125.8 MHz, and ^19^F at 470.5 MHz), a Bruker Avance III HD (^1^H at 400.1 MHz and ^13^C at 100.6 MHz), or a Bruker Avance DPX 300 (^1^H at 299.9 MHz, 13C at 75.4 MHz, and ^19^F at 282 MHz). Chemical shifts (δ) are expressed in ppm recorded using the residual solvent as the internal reference in all cases. Signal splitting patterns are described as singlet (s), doublet (d), triplet (t), quartet (q), multiplet (m), broad (br), or a combination thereof. LC–MS analyses were performed with either an Agilent HPLC 1100 series connected to a Bruker Daltonics MicrOTOF or an Agilent Technologies 1200 series HPLC connected to an Agilent Technologies 6130 quadrupole LC/MS, where both instruments were connected to an Agilent diode array detector. LC–MS chromatographic separations were conducted with either a Waters XBridge C18 column, 50 mm × 2.1 mm, 3.5 μm particle size, or a Waters XSelect C18 column, 30 mm × 2.1 mm, 2.5 μm particle size, using a mobile phase of water/acetonitrile +0.1% HCOOH or water/acetonitrile +0.1% NH_3_. High-resolution electrospray measurements were performed on a Bruker Daltonics MicrOTOF mass spectrometer. Preparative HPLC separations were performed with a Waters mass-directed HPLC (system fluidics organizer, 2545 binary gradient module, 2 × 515 HPLC pumps, 2767 sample manager) connected in parallel to a Waters 3100 mass detector and a 2998 photodiode array detector.^22^ HPLC chromatographic separations were conducted using a Waters XBridge C18 column, 19 × 100 mm, 5 μm particle size, using a mobile phase of water/acetonitrile +0.1% NH_3_. Note that a number of peaks in the ^1^H-NMR of the amino-thiazoles and intermediates are broad, and in some cases, one of the cyclohexyl CH peaks is not observed, presumably due to restricted rotation. In addition, some of the peaks are not observed in the ^13^C-NMR due to the large proportion of quaternary carbons and fluorine coupling. Purity of compounds was assessed by LC–MS, and purity is ≥95%. Key LC–MS traces are shown in the Supporting Information.

### Chemical Synthesis–Experimental Protocols (Intermediates)

#### General Methods

##### General Method A (Scheme 2, Compounds **20b**–**e**, **h**)

A solution of substituted benzaldehyde (1 equiv) and amine (1.5 equiv) in CH_2_Cl_2_ (∼0.06 M) was stirred at room temperature under N_2_ for ∼1 h. Sodium triacetoxyborohydride (2 equiv) was then added in portions and the reaction was stirred further for 16 h at room temperature. The reaction was then washed with water, or a solution of NaHCO_3_ (satd. aq.), the layers were separated, the organics were dried over MgSO_4_ and filtered, and the solvent was removed under reduced pressure.

##### General Method B (Scheme 2, Compounds **21a**–**e**, **g**, **h**)

A solution of nitrile (1 equiv) in PhMe was added dropwise to a degassed solution of methylmagnesium bromide (3.0 M in diethylether, 2–3 equiv) in anhydrous toluene, and the mixture was heated to 110 °C for 2 h. The reaction was then cooled to room temperature and adjusted to pH 2 with 2 M HCl aq. and subsequently heated to 110 °C for an additional 30 min. The mixture was then basified with NaOH (2 M, aq) to pH 11, extracted with EtOAc, dried over MgSO_4_, filtered, and concentrated under reduced pressure.

##### General Method C (Scheme 2, Compounds **22a**–**h**)

A solution of molecular bromine (1.1 equiv) in hydrogen bromide (33% (w/v) in acetic acid, 20 equiv) was added to the ketone substrate (1 equiv) and heated at 60 **°**C for 2 h. The reaction was then cooled to room temperature, diluted with diethyl ether, and stirred further for 2 h. The resulting solid was collected by filtration, washed with diethyl ether, and dried to give the product salt as a brown solid.

##### General Method D (Scheme 3, Compounds **1–7**, **10**, **11**, **29**)

A solution of cyanamide (1.2 equiv) in MeCN was added to potassium *tert*-butoxide (1 M in THF, 1.3–2.2 equiv) at room temperature and stirred for 15 min. *N*-(4-isothiocyanatocyclohexyl)-2-methyl-propane-1-sulfonamide (**17**) (1 equiv) was then added as a solution in MeCN/^*t*^BuOH (1:1) and stirred further for 15 min. Subsequently, a solution of the appropriate α-bromo-ketone (1 equiv) was then added as a solution in MeCN/^*t*^BuOH (1:1), and the reaction was stirred for 12 h at RT.

##### Synthesis of *tert*-Butyl (*trans*-4-(2-Methylpropylsulfonylamido)cyclohexyl)carbamate (Details Reported Previously^12,13^) **(15)**

A solution of *n*-BuLi (2.5 M in hexanes) (16.4 mL, 41.1 mmol) was added dropwise to a solution of *tert*-butyl (*trans*-4-aminocyclohexyl)carbamate (**14**) (8.00 g, 37.3 mmol) in THF (100 mL) at −78 °C. The reaction was allowed to warm to −10 °C and stirred for 15 min, then subsequently cooled to −78 °C, and followed by the dropwise addition of neat 2-methylpropane-1-sulfonyl chloride (7.74 g, 49.4 mmol). The reaction was then allowed to warm to room temperature for over 12 h before quenching with aqueous 2 M HCl. The reaction mixture was extracted with EtOAc (3 × 50 mL), dried over magnesium sulfate, and concentrated under reduced pressure to give a white solid. The crude product was triturated from ether/hexane to give a white solid (4.88 g, 39%). ^1^H NMR (500 MHz, CDCl_3_) δ: 4.39 (1H, br d, *J* = 7 Hz, NH), 4.22 (1H, d, *J* = 7.7 Hz, NH), 3.45–3.35 (1H, m, CH), 3.34–3.21 (1H, m, CH), 2.93 (2H, d, *J* = 6.4 Hz, CH_2_), 2.31–2.25 (1H, m, CH), 2.20–2.00 (4H, m, 4 × cyclohexyl CH), 1.45 (9H, s, ^t^Bu), 1.45–1.14 (4H, m, 4 × cyclohexyl CH), 1.10 (6H, d, *J =* 6.7 Hz, 2 × CH_3_). MS (ES−) (%): 333 (100) [M – H]^−^.

##### *N*-(*trans*-4-Aminocyclohexyl)-2-methyl-propane-1-sulfonamide (**16**) (Details Reported Previously^12^)

A solution of 4 M HCl in dioxane (30 mL, 120 mmol) was added to a suspension of carbamate **15** (4.77 g, 14.3 mmol) in CH_2_Cl_2_ (100 mL) at 0 °C under N_2_. The reaction was warmed to room temperature and stirred for 24 h. The resulting precipitate was recovered by filtration and dried to give a white solid (3.88 g, 100%). ^1^H NMR (500 MHz, DMSO-*d*_6_) δ: 8.20 (3H, br s, NH_3_^+^), 7.09 (1H, d, *J* = 7.5 Hz, NH), 3.10–2.98 (1H, m, CH), 2.98–2.85 (3H, m, CH & CH_2_), 2.13–2.02 (1H, m, CH), 1.98–1.86 (4H, m, 4 × cyclohexyl CH), 1.48–1.25 (4H, m, 4 × cyclohexyl CH), 1.03 (6H, d, *J* = 6.7 Hz, 2 × CH_3_). MS (ES+) (%): 235 (100) [M + H]^+^, 469 (30) [2 M + H]^+^.

##### *N*-(*trans*-4-Isothiocyanatocyclohexyl)-2-methyl-propane-1-sulfonamide (**17**)

Solid 1,1′-thiocarbonyldiimidazole (1.13 g, 6.34 mmol) was added to a solution of sulfonamide **16** (1.35 g, 5.76 mmol) in CH_2_Cl_2_ (40 mL) at room temperature, and the reaction was stirred overnight. The reaction was washed with water, the layers were separated, the CH_2_Cl_2_ layer was dried over magnesium sulfate, filtered, and the was solvent removed under reduced pressure. The crude product was purified by silica column chromatography (10–60% EtOAc in hexane) to give a white solid (1.40 g, 88%). ^1^H NMR (500 MHz, DMSO-*d*_6_) δ: 7.05 (1H, d, *J* = 7.3 Hz, NH), 3.80–3.70 (1H, m, CH), 3.21–3.13 (1H, m, CH), 2.88 (2H, d, *J* = 6.4 Hz, CH_2_), 2.12–2.01 (3H, m, CH & cyclohexyl CH), 1.63–1.54 (2H, m, cyclohexyl CH), 1.36–1.26 (2H, m, cyclohexyl CH), 1.02 (6H, d, 2 × CH_3_). MS (ES+) (%): 294 (100) [M + NH_4_]^+^.

##### 2,6-Difluoro-4-formylbenzonotrile (**19**)

A solution of 4-bromo-2,6-difluoro-benzonitrile (**18**) (10 g, 45.9 mmol) in THF (80 mL) was added dropwise to isopropyl magnesium chloride (2.0 M in THF, 20 mL, 55.1 mmol) under an atmosphere of N_2_ at 0 °C, and the reaction was stirred for 1 h. Neat 1-formylpiperidine (6.23 g, 55.1 mmol) was then added and the reaction was stirred further for 1 h. The reaction was quenched by the addition of a solution of HCl (1 M, aq., 50 mL), and the resultant mixture was extracted with CH_2_Cl_2_ (3 × 100 mL). The organic extracts were combined, dried over MgSO_4_, and concentrated under reduced pressure. The crude product was purified by silica column chromatography (1–30% EtOAc in hexane) to give the title compound as a yellow liquid (5.2 g, 68%). ^1^H NMR (300 MHz, CDCl_3_) δ: 10.03 (1H, dd, *J* = 1.3, 1.3 Hz, CHO), 7.61–7.59 (2H, m, 2 × ArH).

##### 4-((Dimethylamino)methyl)-2,6-difluorobenzonotrile (**20a**)

A solution of dimethylamine (2.0 M in THF, 26.8 mL, 53.6 mmol) was added to a solution of 2,6-difluoro-4-formyl-benzonitrile (**19**) (1.79 g, 10.7 mmol) in CH_2_Cl_2_ (50 mL) and stirred at room temperature for 2 h. Solid sodium triacetoxyborohydride (5.36 g, 21.4 mmol) was then added in portions, and the reaction was stirred overnight at room temperature. The reaction was quenched by the addition of water followed by extraction with CH_2_Cl_2_ (2 × 50 mL). The extracts were combined, dried over MgSO_4_, and concentrated under reduced pressure. The crude product was purified by silica column chromatography (1–10% MeOH in CH_2_Cl_2_) to give the product amine as a yellow liquid (0.603 g, 28%). ^1^H NMR (400 MHz, CDCl_3_) δ: 7.08–7.06 (2H, m, 2 × ArH), 3.48 (2H, s, CH_2_), 2.28 (6H, s, 2 × CH_3_). ^19^F{^1^H} NMR (470 MHz, CDCl_3_) δ: −104.4 (s, ArF). ^13^C NMR (125 MHz, CDCl_3_) δ: 163.2 (dd, *J*_CF_ = 259, 3.8 Hz, CF), 150.1 (weak, confirmed by HMBC), 111.8 (dd, *J*_CF_ = 20, 2.5 Hz, C), 109.3 (weak), 90.7 (weak, confirmed by HMBC), 63.3, 45.4. Note that three of the quaternary carbon peaks are very weak due to splitting from carbon-fluorine coupling. MS (ES+) (%): 197 (100) [M + H]^+^. HRMS (ES+): calcd. for C_10_H_11_F_2_N_2_ [M + H]^+^, 197.0885; found, 197.0883 (1.1 ppm).

##### 1-(4-((Dimethylamino)methyl)-2,6-difluorophenyl)ethan-1-one (**21a**)

A solution of nitrile **20a** (0.603 g, 3.07 mmol) in PhMe (20 mL) was reacted with methylmagnesium bromide (3.0 M in diethylether, 2.05 mL, 6.15 mmol) in anhydrous toluene (20 mL) according to general method B. The crude product was purified by silica column chromatography (0–5% MeOH in CH_2_Cl_2_) to give the title compound as an oil (0.337 g, 51%). ^1^H NMR (500 MHz, CDCl_3_) δ: 6.96–6.94 (2H, m, 2 × ArH), 3.41 (2H, s, CH_2_), 2.59 (3H, dd, *J* = 1.8 Hz), 2.25 (6H, s, 2 × CH_3_). ^19^F{^1^H} NMR (470 MHz, CDCl_3_) δ: −112.2 (s, ArF). ^13^C NMR (125 MHz, CDCl_3_) δ: 194.6, 160.2 (dd, *J*_CF_ = 253, 9 Hz, CF), 146.0 (dd, *J*_CF_ = 10, 10 Hz), 116.7–116.4 (m, weak due to C–F coupling, confirmed by HMBC), 112.1–111.9 (m), 63.2, 45.3, 32.3. MS (ES+) (%): 214 (100) [M + H]^+^. HRMS (ES+): calcd. for C_11_H_14_F_2_NO [M + H]^+^, 214.1038; found, 214.1038 (0.1 ppm).

##### 2-Bromo-1-(4-((dimethylamino)methyl)-2,6-difluorophenyl)ethan-1-one Hydrobromide (**22a**)

A solution of molecular bromine (0.094 g, 0.588 mmol) in hydrogen bromide (33% (w/v) in acetic acid, 5.8 mL) was reacted with ketone **21a** (0.114 g, 0.54 mmol) according to general method C to give the product salt as a brown solid (0.129 g, 65%), which was used without further purification. ^1^H NMR (500 MHz, DMSO-*d*_6_) δ: 9.86 (1H, br s, NH^+^), 7.55–7.43 (2H, m, 2 × ArH), 4.77 (2H, s, CH_2_), 4.38 (2H, br s, CH_2_), 2.78, (6H, br s, NMe_2_). ^19^F{^1^H} NMR (470 MHz, CDCl_3_) δ: −110.4 (s, ArF). MS (ES+) (%): 292 (100) [^79^BrM + H]^+^, 294 (100) [^81^BrM + H]^+^.

##### 2,6-Difluoro-4-((4-methylpiperzin-1-yl)methyl)benzonitrile (**20b**)

A solution of 2,6-difluoro-4-formyl-benzonitrile (1.04 g, 6.21 mmol) and 1-methylpiperazine (0.559 g, 5.59 mmol) in CH_2_Cl_2_ (100 mL) was reacted according to general method A. The crude product was purified by silica column chromatography (1–40% MeOH in CH_2_Cl_2_) to give the title compound as a yellow oil (1.03 g, 66%). ^1^H NMR (500 MHz, CDCl_3_) δ: 7.11–7.08 (2H, m, 2 × ArH), 3.55 (2H, s, CH_2_), 2.62–2.45 (8H, m, 4 × CH_2_), 2.31 (3H, s, CH_3_). MS (ES+) (%): 252 (100) [M + H]^+^.

##### 1-(2,6-Difluoro-4-((4-methylpiperazin-1-yl)methyl)phenyl)ethan-1-one (**21b**)

A solution of nitrile **20b** (1.03 g, 4.10 mmol) in PhMe (50 mL) was reacted with methylmagnesium bromide (3.0 M in diethylether, 4.1 mL, 12.3 mmol) according to general method B. The crude product was purified by silica column chromatography (1–10% MeOH in CH_2_Cl_2_) to give the title compound as an amber oil (0.398 g, 36%). ^1^H NMR (300 MHz, CDCl_3_) δ: 7.02–6.96 (2H, m, 2 × ArH), 3.51 (2H, s, CH_2_), 2.61 (3H, dd, *J* = 1.9, 1.9 Hz, CH_3_), 2.57–2.39 (8H, m, 4 × CH_2_), 2.32 (3H, s, NMe).

##### 2-Bromo-1-(2,6-difluoro-4-(4-methylpiperazin-1-yl)methyl)ethan-1-one Hydrobromide (**22b**)

Ketone **21b** (0.398 g, 1.48 mmol) was reacted according to general method C to give the title compound as a brown solid (0.608 g, 96%). MS (ES+) (%): 347 (100) [^79^BrM + H]^+^, 349 (100) [^81^BrM + H]^+^.

##### 2,6-Difluoro-4-(morpholinomethyl)benzonitrile (**20c**)

A solution of 2,6-difluoro-4-formyl-benzonitrile (**19**) (0.984 g, 5.89 mmol) and morpholine (0.769 g, 8.83 mmol) in CH_2_Cl_2_ (100 mL) was reacted according to general method A. The crude product was purified by silica column chromatography (1–5% MeOH in CH_2_Cl_2_) to give the title compound as an amber oil (0.952 g, 68%). ^1^H NMR (500 MHz, CDCl_3_) δ: 7.13–7.11 (2H, m, 2 × ArH), 3.76–3.73 (4H, m, 2 × CH_2_), 3.55 (2H, s, CH_2_), 2.49–2.47 (4H, m, 2 × CH_2_). MS (ES+) (%): 239 (100) [M + H]^+^.

##### 1-(2,6-Difluoro-4-(morpholinomethyl)phenyl)ethan-1-one (**21c**)

A solution of nitrile **20c** (0.952 g, 4.10 mmol) in PhMe (25 mL) was reacted with methylmagnesium bromide (3.0 M in diethyl ether, 4.0 mL, 12.0 mmol) according to general method B. The crude product was purified by silica column chromatography (10–60% EtOAc in hexane) to give the title compound as a yellow oil (0.453 g, 44%). ^1^H NMR (300 MHz, CDCl_3_) δ: 6.93–6.86 (2H, m, 2 × ArH), 3.67–3.64 (4H, m, 2 × CH_2_), 3.41 (2H, s, CH_2_), 2.52–2.51 (3H, m, Me), 2.39–2.36 (4H, m, 2 × CH_2_). MS (ES+) (%): 256 (100) [M + H]^+^.

##### 2-Bromo-1-(2,6-difluoro-4-(morpholinomethyl)phenyl)ethan-1-one Hydrobromide (**22c**)

Ketone **21c** (0.453 g, 1.77 mmol) was reacted according to general method C to give the title compound as a brown solid (0.252 g, 34%). ^1^H NMR (300 MHz, DMSO-*d*_6_) δ: 10.14 (1H, br s, NH^+^), 7.55–7.47 (2H, m, 2 × ArH), 4.77 (2H, s, CH_2_), 4.46 (2H, br s, CH_2_), 4.07–3.65 (4H, m, 2 × CH_2_), 3.38–3.04 (4H, m, 2 × CH_2_). MS (ES+) (%): 334 (100) [^79^BrM + H]^+^, 336 (100) [^81^BrM + H]^+^.

##### 2,6-Difluoro-4-(pyrrolidin-1-ylmethyl)benzonitrile 2,6-Difluoro-4-(pyrrolidin-1-ylmethyl)benzonitrile (**20d**)

A solution of 2,6-difluoro-4-formyl-benzonitrile (**19**) (1.04 g, 6.21 mmol) and pyrrolidine (0.397 g, 5.59 mmol) in CH_2_Cl_2_ (100 mL) was reacted according to general method A. The crude product was purified by silica column chromatography (1–4% MeOH in CH_2_Cl_2_) to give the title compound as a clear oil (1.12 g, 72%). ^1^H NMR (500 MHz, CDCl_3_) δ: 7.11–7.08 (2H, m, 2 × ArH), 3.67 (2H, s, CH_2_), 2.56–2.49 (4H, m, 2 × CH_2_), 1.84–1.81 (4H, m, 2 × CH_2_).

##### 1-(2,6-Difluoro-4-(pyrrolidin-1-ylmethyl)phenyl)ethenone (**21d**)

A solution of nitrile **20d** (0.660 g, 2.97 mmol) in PhMe (40 mL) was reacted with methylmagnesium bromide (3.0 M in diethyl ether, 3.0 mL, 8.91 mmol) according to general method B to give the title compound as an amber oil (0.150 g, 21%). ^1^H NMR (300 MHz, CDCl_3_) δ: 7.00–6.94 (2H, m, 2 × ArH), 3.62 (2H, s, CH_2_), 2.60–2.58 (3H, m, CH_3_), 2.54–2.50 (4H, m, 2 × CH_2_), 1.83–1.79 (4H, m, 2 × CH_2_). MS (ES+) (%): 240 (100) [M + H]^+^.

##### 2-Bromo-1-[2,6-difluoro-4-(pyrrolidin-1-ylmethyl)phenyl]ethanone Hydrobromide (**22d**)

Ketone **21d** (0.150 g, 0.627 mmol) was reacted according to general method C to give the title compound as a brown solid (0.143 g, 57%). ^1^H NMR (500 MHz, DMSO-*d*_6_) δ: 9.97 (1H, s, NH^+^), 7.55–7.51 (2H, m, 2 × ArH), 4.78 (2H, s, CH_2_), 4.48–4.45 (2H, m, CH_2_), 3.48–3.41 (2H, m, 2 × C*H*H), 3.15–3.08 (2H, m, 2 × C*H*H), 2.09–2.01 (2H, m, 2 × C*H*H), 1.92–1.86 (2H, m, 2 × C*H*H). MS (ES+) (%): 318 (100) [^79^BrM + H]^+^, 320 (100) [^81^BrM + H]^+^.

##### 4-(Azetidin-1-ylmethyl)-2,6-difluorobenzonitrile (**20e**)

A solution of 2,6-difluoro-4-formyl-benzonitrile (**19**) (4.57 g, 27.4 mmol) and azetidine (5.00 g, 87.6 mmol) in CH_2_Cl_2_ (100 mL) was reacted according to general method A. The crude product was purified by silica column chromatography (1–10% MeOH in CH_2_Cl_2_) to give the title compound as a yellow oil (2.12 g, 37%). ^1^H NMR (400 MHz, CDCl_3_) δ: 7.05–7.02 (2H, m, 2 × ArH), 3.71 (2H, s, CH_2_), 3.36 (4H, dd, *J* = 7.2, 7.2 Hz, 2 × CH_2_), 2.21–2.14 (2H, m, CH_2_).

##### 1-(4-(Azetidin-1-ylmethyl)-2,6-difluorophenyl)ethan-1-one (**21e**)

A solution of nitrile **20e** (1.06 g, 5.08 mmol) in PhMe (20 mL) was reacted with methylmagnesium bromide (3.0 M in diethyl ether, 3.4 mL, 10.2 mmol) according to general method B. The crude product was purified by silica column chromatography (0–10% MeOH in CH_2_Cl_2_) to give the title compound as a solid (0.287 g, 25%). ^1^H NMR (400 MHz, CDCl_3_) δ: 6.94–6.89 (2H, m, 2 × ArH), 3.57 (2H, s, CH_2_), 3.26 (4H, dd, *J* = 7, 7 Hz, 2 × CH_2_), 2.60–2.59 (3H, m, CH_3_), 2.174–2.10 (2H, m, CH_2_). MS (ES+) (%): 226 (100) [M + H]^+^, 267 (90) [M + H + MeCN]^+^.

##### 1-(4-(Azetidin-1-ylmethyl)-2,6-difluorophenyl)-2-bromoethan-1-one Hydrobromide (**22e**)

Ketone **21e** (0.278 g, 1.23 mmol) was reacted according to general method C to give the title compound as a brown semi-solid (0.400 g, 84%). MS (ES+) (%): 304 (100) [^79^BrM + H]^+^, 306 (100) [^81^BrM + H]^+^, 345 (50) [^79^BrM + H + MeCN]^+^, 347 (50) [^81^BrM + H + MeCN]^+^.

##### 1-(3,5-Dichlorophenyl)-*N*,*N*-dimethylmethanamine (**24**)

1,3-Dichloro-5-(chloromethyl)benzene (**23**) (5.0 g, 25.58 mmol) was dissolved in a solution of dimethyamine (33% in EtOH, 25 mL) and heated under microwave irradiation at 100 °C for 20 min. The reaction was then concentrated under reduced pressure, diluted with CH_2_Cl_2_ (50 mL), and washed with water (20 mL). The layers were subsequently separated, the organic layer was dried over MgSO_4_, filtered, and concentrated under reduced pressure to give amine **24** as a brown liquid (4.98 g, 95%) that was used without further purification. ^1^H NMR (400 MHz, DMSO-*d*_6_) δ: 7.44–7.42 (1H, m, ArH), 7.32–7.30 (2H, m, 2 × ArH), 3.39 (2H, s, CH_2_), 2.12 (6H, s, 2 × CH_3_).

##### 2,6-Dichloro-4-((dimethylamino)methyl)benzaldehyde (**25**)

*n*-Butyllithium (2.5 M in hexanes, 9.11 mL, 22.8 mmol) was added dropwise to a solution of 1-(3,5-dichlorophenyl)-*N*,*N*-dimethyl-methanamine (**24**) (4.65 g, 22.8 mmol) in THF (50 mL) at −78 °C under N_2_. The reaction was then stirred for 30 min prior to the addition of DMF (5 g, 68.3 mmol). The reaction was subsequently stirred at −78 °C for an additional 30 min, warmed to 0 °C, quenched by the addition of water, and extracted with CH_2_Cl_2_ (3 × 50 mL). The combined organic extracts were dried over MgSO_4_, filtered, and concentrated under reduced pressure. The crude product was purified by silica column chromatography (0–60% EtOAc in heptane) to give the title compound as an amber liquid (2.62 g, 50%). ^1^H NMR (400 MHz, DMSO-*d*_6_) δ; 10.38 (1H, s, CHO), 7.52 (2H, s, 2 × ArH), 3.47 (2H, s, CH_2_), 2.16 (6H, s, NMe_2_).

##### 1-(2,6-Dichloro-4-((dimethylamino)methyl)phenyl)ethan-1-ol (**26**)

Methyl magnesuim bromide (3 M in diethyl ether, 2.0 mL, 6.0 mmol) was added dropwise to a solution of aldehyde **25** (1.06 g, 4.57 mmol) in THF (10 mL) at 0 °C. The reaction was allowed to warm with stirring for 30 min before being quenched with HCl (1 M, aq). The reaction was subsequently diluted with CH_2_Cl_2_, dried over MgSO_4_, filtered, and concentrated under reduced pressure to give the product alcohol as a solid (1.01 g, 89%). ^1^H NMR (500 MHz, DMSO-*d*_6_) δ: 7.53 (2H, s, 2 × ArH), 5.48–5.40 (2H, m, CH & OH), 3.92 (2H, br s, CH_2_), 2.50 (6H, s, NMe_2_), 1.46 (3H, d, *J* = 6.7 Hz). MS (ES+) (%): 248 (100) [^35^Cl_2_M + H]^+^, 250 (70) [^35^Cl^37^ClM + H]^+^.

##### 1-(2,6-Dichloro-4-((dimethylamino)methyl)phenyl)ethan-1-one (**21f**)

A solution of alcohol **26** (0.556 g, 2.24 mmol) and Dess–Martin periodinane (1.05 g, 2.46 mmol) in CH_2_Cl_2_ (10 mL) was stirred for 2 h at RT. The reaction was then diluted with CH_2_Cl_2_, washed with sodium carbonate (aq, 5% w/v) and water, then dried over MgSO_4_, filtered, and concentrated under reduced pressure to give ketone **21f** (0.441 g, 80%). ^1^H NMR (500 MHz, CDCl_3_) δ: 7.32–7.31 (2H, m, 2 × ArH), 3.40 (2H, br s, CH_2_), 2.60 (3H, s, CH_3_), 2.27 (6H, s, NMe_2_). MS (ES+) (%): 246 (100) [^35^Cl_2_M + H]^+^, 248 (65) [^35^Cl^37^ClM + H]^+^, 287 (60) [^35^Cl_2_M + H + MeCN]^+^, 289 (45) [^35^Cl^37^ClM + H + MeCN]^+^.

##### 2-Bromo-1-(2,6-dichloro-4-((dimethylamino)methyl)phenyl)ethan-1-one Hydrobromide (**22f**)

Ketone **21f** (0.441 g, 1.79 mmol) was reacted according to general method C to give the title compound as a brown gum that was used without further purification (0.727 g, quant.). MS (ES+) (%): 324 (60) [^35^Cl_2_^79^BrM + H]^+^, 326 (100) [^35^Cl^37^Cl^79^Br & ^35^Cl_2_^81^Br M + H]^+^, 328 (45) [^37^Cl_2_^79^Br & ^35^Cl^37^Cl^81^Br M + H]^+^.

##### *tert*-Butyl-(4-cyano-3,5-difluorophenethyl)carbamate (**27**)

A mixture of 4-bromo-2,6-difluoro-benzonitrile (**18**, 0.537 g, 2.46 mmol), Pd(dppf)Cl_2_ (0.101 g, 0.12 mmol), cesium carbonate (2.41 g, 7.39 mmol), and potassium 2-(Boc-aminoethyl)trifluoroborate (0.680 g, 2.71 mmol) in toluene (15 mL) and water (2 mL) was heated at 80 °C for 12 h. The reaction was cooled to room temperature and diluted with a solution of NH_4_Cl (satd. aq.). The crude mixture was then extracted with CH_2_Cl_2_ (2 × 50 mL), dried over MgSO_4_, filtered, and the solvent was removed under reduced pressure. The crude product was purified by silica column chromatography (0–30% EtOAc in heptane) to give the title compound as a white solid (0.403 g, 58%). ^1^H NMR (400 MHz, CDCl_3_) δ: 6.93–6.91 (2H, m, 2 × ArH), 4.60 (1H, br s, NH), 3.43–3.38 (2H, m, CH_2_), 2.90 (2H, t, *J* = 6.9 Hz, CH_2_), 1.45 (9H, s, ^t^Bu).

##### 4-(2-Aminoethyl)-2,6-difluorobenzonitrile (**28**)

HCl (4 M in dioxane, 4.24 mL, 17 mmol) was added to a suspension of carbamate **27** (0.479 g, 1.70 mmol) in CH_2_Cl_2_ (100 mL) at room temperature. The reaction was then stirred for 12 h at RT, and the resultant white precipitate was collected by filtration and washed with CH_2_Cl_2_. The solid was then partitioned between a sodium carbonate solution (5% w/v, 200 mL) and CH_2_Cl_2_ (250 mL) and stirred vigorously until solubilized. The layers were then separated, and the aqueous layer was further extracted with CH_2_Cl_2_ (4 × 50 mL). The organic extracts were combined, washed with NaCl solution (satd. aq.), dried over MgSO_4_, filtered, and concentrated under reduced pressure to give the title compound as a light beige solid (0.235 g, 76%). ^1^H NMR (400 MHz, CDCl_3_) δ: 6.95–6.92 (2H, m, 2 × ArH), 3.01 (2H, t, *J* = 6.8 Hz, CH_2_), 2.80 (2H, dt, *J* = 6.8, 0.4 Hz, 2H), 1.24 (2H, br s, NH).

##### 4-(2-Dimethylamino)ethyl-2,6-difluorobenzonitrile (**20g**)

A mixture of 4-(2-aminoethyl)-2,6-difluoro-benzonitrile (**28**, 0.450 g, 2.47 mmol) and paraformaldehyde (0.371 g, 12.4 mmol) in formic acid (5 mL) was heated to 100 °C for 12 h. The reaction mix was then basified with NaOH (2 M, aq) and extracted with EtOAc (3 × 50 mL). The organic extracts were combined, washed with NaCl solution (satd. aq.), dried over MgSO_4_, and concentrated under reduced pressure. The crude product was purified by silica column chromatography (0–5% MeOH in CH_2_Cl_2_) to give the title compound as an orange oil (0.250 g, 48%). ^1^H NMR (500 MHz, CDCl_3_) δ: 6.96–6.93 (2H, m, 2 × ArH), 2.84 (2H, t, *J* = 7.4 Hz, CH_2_), 2.57 (2H, t, *J* = 7.4 Hz, CH_2_), 2.28 (6H, s, NMe_2_). MS (ES+) (%): 211 (50) [M + H]^+^, 252 (100) [M + H + MeCN]^+^.

##### 1-(4-(2-Dimethylamino)ethyl-2,6-difluorophenyl)ethan-1-one (**21g**)

A solution of nitrile **20g** (1.06 g, 5.08 mmol) in PhMe (20 mL) was reacted with methylmagnesium bromide (3.0 M in diethyl ether, 0.856 mL, 2.57 mmol) according to general method B. The crude product was purified by silica column chromatography (0–5% MeOH in CH_2_Cl_2_) to give the title compound as an oil (0.130 g, 45%). ^1^H NMR (500 MHz, CDCl_3_) δ: 6.83–6.80 (2H, m, 2 × ArH), 2.77 (2H, t, *J* = 7.6 Hz, CH_2_), 2.58–2.52 (5H, m, CH_2_ & CH_3_), 2.28 (6H, s, 2 × CH_3_).

##### 2-Bromo-1-(4-(2-(dimethylamino)ethyl)-2,6-difluorophenyl)ethan-1-one Hydrobromide (**22g**)

Ketone **21g** (0.130 g, 0.57 mmol) was reacted according to general method C to give the title compound as a brown solid that was used without further purification (0.221 g, quant.). MS (ES+) (%): 306 (100) [^79^BrM + H]^+^, 308 (90) [^81^BrM + H]^+^, 347 (50) [^79^BrM + H + MeCN]^+^, 349 (55) [^81^BrM + H + MeCN]^+^.

##### 2,6-Difluoro-4-(((4-methoxybenzyl)(methyl)amino)methyl)benzonitrile (**20h**)

A solution of 2,6-difluoro-4-formyl-benzonitrile (**19**) (4.57 g, 27.4 mmol) and 4-methoxy-*N*-methylbenzylamine (3.62 g, 23.9 mmol) in CH_2_Cl_2_ (100 mL) was reacted according to general method A. The crude product was purified by silica column chromatography (0–50% EtOAc in heptane) to give the title compound as a yellow oil (2.25 g, 62%). ^1^H NMR (400 MHz, CDCl_3_) δ: 7.29–7.25 (2H, m, 2 × ArH), 7.14–7.09 (2H, m, 2 × ArH), 6.92–6.87 (2H, m, 2 × ArH), 3.83 (3H, s, CH_3_), 3.54–3.52 (4H, m, 2 × CH_2_), 2.22 (3H, m, CH_3_). MS (ES+) (%): 121 (100) [C_8_H_9_O]^+^, 303 (42) [M + H]^+^.

##### 1-(2,6-Difluoro-4-(((4-methoxybenzyl)(methyl)amino)methyl)phenyl)ethan-1-one (**21h**)

A solution of nitrile **20h** (2.25 g, 7.45 mmol) in PhMe (50 mL) was reacted with methylmagnesium bromide (3.0 M in diethyl ether, 3.72 mL, 11.2 mmol) according to general method B. The crude product was purified by silica column chromatography (0–20% EtOAc in heptane) to give the title compound as an oil (1.789 g, 75%). ^1^H NMR (400 MHz, CDCl_3_) δ: 7.30–7.25 (2H, m, 2 × ArH), 7.03–6.97 (2H, m, 2 × ArH), 6.92–6.87 (2H, m, 2 × ArH), 3.83 (3H, s, CH_3_), 3.51 (2H, s, CH_2_), 3.48 (2H, s, CH_2_), 2.61 (3H, s, CH_3_), 2.20 (3H, s, CH_3_). MS (ES+) (%): 121 (25) [C_8_H_9_O]^+^, 320 (100) [M + H]^+^.

##### 2-Bromo-1-(2,6-difluoro-4-(((4-ethoxybenzyl)(methyl)amino)methyl)phenyl)ethan-1-one Hydrobromide (**22h**)

Ketone **21h** (0.555 g, 1.74 mmol) was reacted according to general method C to give the title compound as an orange gum that was used without further purification (0.832 g, quant.). MS (ES+) (%): 398 (98) [^79^BrM + H]^+^, 400 (100) [^81^BrM + H]^+^.

##### *N*-((*trans*)-4-((4-Amino-5-(4-(((4-methoxybenzyl)(methyl)amino)methyl)benzoyl)thiazol-2-yl)amino)cyclohexyl)-2-methylpropane-1-sulfonamide (**29**)

Cyanamide (0.88 g, 2.08 mmol) in MeCN (3 mL), potassium *tert*-butoxide (1 M in THF, 2.26 mL), **17** (0.480 g, 1.74 mmol) in MeCN/^*t*^BuOH (1:1, 4 mL), and **22h** (0.832 mg, 1.74 mmol) in MeCN/^*t*^BuOH (1:1, 6 mL) were reacted according to general method D. The reaction was then concentrated under reduced pressure, and the resultant solid was triturated with diethyl ether and recovered by filtration. The crude solid thiazole was dissolved in CH_2_Cl_2_/MeOH (1:1), basified with sodium carbonate solution (5% w/v, aq), and extracted with CH_2_Cl_2_ (2 × 20 mL). The combined organics were washed with a solution of NaCl (satd. aq.), dried over MgSO_4_, filtered, and concentrated under reduced pressure. The crude product was purified by silica column chromatography (0–100% EtOAc in heptane) to give **29** as a yellow solid (0.628 g, 56%). ^1^H NMR (400 MHz, CDCl_3_) δ: 7.30–7.28 (2H, m, 2 × ArH), 7.01–6.96 (2H, m, 2 × ArH), 6.92–6.88 (2H, m, 2 × ArH), 5.56–5.51 (1H, m, NH), 4.11–4.07 (1H, m, NH), 3.84 (3H, s, CH_3_), 3.51 (2H, s, CH_2_), 3.48 (2H, s, CH_2_), 3.29 (2H, br s, NH_2_), 2.92 (2H, d, *J* = 6.5 Hz, CH_2_), 2.30–2.23 (1H, m, CH), 2.22 (3H, s, CH_3_), 2.20–2.09 (4H, m, 4 × C*H*H), 1.41–1.30 (4H, m, 4 × C*H*H), 1.12 (6H, d, *J* = 6.8 Hz, 2 × CH_3_). Note that two CH peaks are not observed. MS (ES+) (%): 636 (100) [M + H]^+^.

##### *tert*-Butyl-(3,5-difluorobenzyl)carbamate (**30**)

Neat triethylsilane (49.1 g, 422 mmol) and TFA (32.1 g, 281 mmol) were added to a solution of 3,5-difluorobenzaldehyde (**29**) (20 g, 141 mmol) and *tert*-butyl carbamate (49.5 g, 422 mmol) in MeCN (400 mL) at RT, and the reaction was stirred at 75 °C for 16 h. A NaHCO_3_ solution (satd. aq. 250 mL) was added to the mixture to quench the reaction. The MeCN was then removed under reduced pressure, and the aqueous layer was extracted with EtOAc (3 × 100 mL). The combined organics were dried over Na_2_SO_4_, filtered, and concentrated under reduced pressure. The crude residue was purified by column chromatography (0–50% EtOAc in petroleum ether) to give the title compound as a white solid (5.20 g, 15%). ^1^H NMR (300 MHz, CDCl_3_) δ: 6.82–6.79 (2H, m, 2 × ArH), 6.73–6.69 (1H, m, ArH), 4.94 (1H, br s, NH), 4.30 (2H, d, *J* = 6 Hz, CH_2_), 1.47 (9H, s, ^*t*^Bu).

##### 2,4-Dichlorothiazole-5-carbaldehyde (**32**)

DMF (125 g, 171 mmol) was added dropwise to a suspension of thiazolidine-2,4-dione (**31**) (100 g, 854 mmol) in POCl_3_ (876 g, 5.71 mol) at 0 °C. The reaction was then degassed and purged with N_2_ three times. The mixture was then stirred at 60 °C for 1 h and then stirred at 110 °C for an additional 4 h. The reaction mixture was subsequently cooled to 20 °C and concentrated under reduced pressure. The residue was poured into water (3 L) and stirred for 20 min. The aqueous phase was then extracted with EtOAc (3 × 2 L). The extracts were combined and washed with a solution of NaCl (satd. aq. 2 × 1 L), dried over anhydrous Na_2_SO_4_, filtered, and concentrated under reduced pressure. The residue was purified by silica column chromatography (20–100% EtOAc in petroleum ether) to give the product aldehyde as a yellow solid (49.0 g, 32%). ^1^H NMR (400 MHz, CDCl_3_) δ: 9.97 (s, 1H).

##### *tert*-Butyl-(4-((dichlorothiazol-5-yl)(hydroxyl)methyl)-3,5-difluorobenzyl)carbamate (**33**)

To a solution of *tert*-butyl *N*-[(3,5-difluorophenyl)methyl]carbamate (**30**) (0.300 g, 1.23 mmol) in THF (5 mL) was added TMEDA (0.357 g, 3.08 mmol). The mixture was stirred at −78 °C for 30 min. To the reaction was added *n*-BuLi (2.5 M in hexanes, 1.23 mL, 3.08 mmol) dropwise. The mixture was stirred at −78 °C for 1 h. Then, 2,4-dichlorothiazole-5-carbaldehyde (269 mg, 1.48 mmol) dissolved in THF (3 mL) was added to the mixture and stirred at 0 °C for 1 h. A solution of NH_4_Cl (satd. aq. 10 mL) was then added, and the reaction was extracted with EtOAc (2 × 10 mL). The combined organic extracts were dried over anhydrous Na_2_SO_4_, filtered, and concentrated under reduced pressure. The crude product was then purified by silica column chromatography (15–25% EtOAc in petroleum ether) to give the title compound as a yellow solid (0.800 g, significant traces of EtOAc, therefore no yield given). ^1^H NMR (400 MHz, DMSO-*d*_6_) δ: 7.50 (1H, t, *J* = 6.0 Hz, NH), 7.13 (1H, d, *J* = 4.4 Hz, OH), 6.96–6.93 (2H, m, 2 × ArH), 6.15 (1H, d, *J* = 4.4 Hz, C*H*OH), 4.12, (2H, d, *J* = 6.0 Hz, CH_2_), 1.37 (9H, s, ^t^Bu). MS (ES+) (%): 369 (100) [^35^Cl_2_M – ^t^Bu + H]^+^, 371 (73) [^35^Cl^37^ClM – ^*t*^Bu + H]^+^.

##### *tert*-Butyl-(4-(2,4-dichlorothiazole-5-carbonyl)-3,5-difluorobenzyl)carbamate (**34**)

To a solution of *tert*-butyl-(4-((dichlorothiazol-5-yl)(hydroxyl)methyl)-3,5-difluorobenzyl)carbamate (**33**) (0.570 g, 1.34 mmol) in CHCl_3_ (12 mL) was added Dess–Martin periodinane (1.14 g, 2.68 mmol). The mixture was then stirred at 15 °C for 30 min. A solution of Na_2_SO_3_ (satd. aq. 20 mL) was then added, and the resultant mixture was extracted with CH_2_Cl_2_ (2 × 30 mL). The organic extracts were combined, dried over anhydrous Na_2_SO_4_, filtered, and concentrated under reduced pressure. The crude product was purified by silica column chromatography (15–25% EtOAc in petroleum ether) to give ketone **34** as a yellow solid (0.350 g, 52%). ^1^H NMR (400 MHz, DMSO-*d*_6_) δ: 7.56–7.54 (1H, m, NH), 7.18–7.16 (2H, m, 2 × ArH), 4.23 (2H, d, *J* = 6.0 Hz), 1.40 (9H, s, ^*t*^Bu). MS (ES+) (%): 367 (100) [^35^Cl_2_M – ^*t*^Bu + H]^+^, 369 (98) [^35^Cl^37^ClM – ^*t*^Bu + H]^+^.

##### *tert*-Butyl-(4-(4-chloro-2-(((*trans*)-4-((2-methylpropyl)sulfonamide)cyclohexyl)amino)thiazole-5-carbonyl)-3,5-difluorobenzyl)carbamte (**35**)

To a solution of *tert*-butyl-(4-(2,4-dichlorothiazole-5-carbonyl)-3,5-difluorobenzyl)carbamte (0.350 g, 0.823 mmol) in dioxane (10 mL) was added amine **16** (0.349 g, 1.49 mmol). The mixture was stirred at 100 °C for 16 h. The reaction was subsequently concentrated under reduced pressure and purified by silica column chromatography (15–33% EtOAc in petroleum ether) to give the title compound as a yellow solid (0.290 g, 49%). ^1^H NMR (300 MHz, DMSO-*d*_6_) δ: 9.33 (1H, br s, NH), 7.53 (1H, t, *J* = 6.0 Hz, NH), 7.08–7.05 (3H, m, 2 × ArH & NH), 4.18 (2H, d, *J* = 6.0 Hz, CH_2_), 3.11–3.08 (1H, m, CH), 2.89 (2H, d, *J* = 6.6 Hz, CH_2_), 2.15–1.90 (5H, m, CH & 4 × C*H*H), 1.40–1.33 (13H, m, ^*t*^Bu & 4 × C*H*H), 1.01 (6H, d, *J* = 6.6 Hz, 2 × CH_3_). Note that one CH peak is not observed. MS (ES+) (%) 621 (100) [^35^Cl_2_ M + H]^+^, 623 (46) [^35^Cl ^37^Cl M + H]^+^.

##### *tert*-Butyl-(4-(4-amino-2(((*trans*)-4-((2-methylpropyl)sulfonamide)cyclohexyl)amino)thiazole-5-carbonyl)-3,5-difluorobenzyl)carbamate (**36**)

To a solution of *tert*-butyl-(4-(4-chloro-2-(((*trans*)-4-((2-methylpropyl)sulfonamide)cyclohexyl)amino)thiazole-5-carbonyl)-3,5-difluorobenzyl)carbamate (**35**) (0.280 g, 0.451 mmol) in EtOH (3 mL) was added NH_3_·H_2_O (1.90 g, 13.5 mmol). The mixture was stirred at 85 °C for 16 h. The mixture was washed with water (10 mL) and extracted with EtOAc (2 × 15 mL). The combined organic extracts were dried over anhydrous Na_2_SO_4_, filtered, and concentrated under reduced pressure to give the product amino-thiazole as a yellow solid (0.250 g, 86%). MS (ES+) (%): 602 (100) [M + H]^+^.

#### Chemistry Experimental–Analogues for Testing

##### *N*-((*trans*)-4-((4-Amino-5-(4-(aminomethyl)-2,6-difluorobenzoyl)thiazol-2-yl)amino)cyclohexyl)-2-methylpropane-1-sulfonamide Hydrochloride (**9**)

TFA (1.91 mL, 25.8 mmol) was added to a solution of *tert*-butyl-(4-(4-amino-2(((*trans*)-4-((2-methylpropyl)sulfonamide)cyclohexyl)amino)thiazole-5-carbonyl)-3,5-difluorobenzyl)carbamate (**36**) (0.250 g, 0.416 mmol) in CH_2_Cl_2_ (15 mL). The mixture was stirred at room temperature for 2 h and subsequently concentrated under reduced pressure. The resultant residue was washed with NaHCO_3_ (satd. aq. 15 mL) and extracted with CH_2_Cl_2_/MeOH (10:1, 5 × 20 mL). The combined organic extracts were dried over anhydrous Na_2_SO_4_, filtered, and concentrated under reduced pressure. The crude product was purified by preparative HPLC [water (+0.05% HCl v/v):MeCN], and the product containing fractions was lyophilized to give the title compound as a yellow solid (0.060 g, 29%). ^1^H NMR (400 MHz, CD_3_OD) δ: 7.39–7.37 (2H, m, 2 × ArH), 4.26 (2H, s, CH_2_), 4.00–3.97 (1H, m, CH), 3.21–3.19 (1H, m, CH), 2.93 (2H, d, *J* = 6.4 Hz, CH_2_), 2.22–2.00 (5H, m, CH & 4 × C*H*H), 1.60–1.35 (4H, m, 4 × C*H*H), 1.09 (6H, d, *J* = 6.8 Hz, 2 × CH_3_). ^13^C NMR (125 MHz, DMSO-*d*_6_) δ: 171.0, 166.4, 158.4 (dd, *J* = 246, 9.7 Hz, CF), 138.5, 119.7, 113.2–113.0 (m), 60.5, 51.3, 41.7, 32.8, 31.0, 24.9, 22.8. Note that one CH and two quaternary carbons are not observed. Note that some quaternary carbon peaks are not strong enough to observe the expected multiplicity. MS (ES+) (%): 251.5 (29) [M + 2H]^2+^, 502 (100) [M + H]^+^. HRMS (ES+): calcd. for C_21_H_30_F_2_N_5_O_3_S_2_ [M + H]^+^, 502.1753; found, 502.1751 (0.4 ppm).

##### *N*-(*trans*)-[4-[[4-Amino-5-[4-(dimethylaminomethyl)-2,6-difluoro-benzoyl]thiazol-2-yl]amino]cyclohexyl]-2-methyl-propane-1-sulfonamide (**2**)

Cyanamide (0.013 g, 0.32 mmol) in MeCN (0.65 mL), potassium *tert*-butoxide (1 M in THF, 0.322 mL), **17** (0.074 g, 0.27 mmol) in MeCN/^*t*^BuOH (1:1, 0.9 mL), and **22a** (0.10 g, 0.27 mmol) were reacted according to general method D. The resulting precipitate was recovered by filtration and then further purified using an SCX cartridge (20% MeOH in CH_2_Cl_2_ → 20% 7 N NH_3_ MeOH in CH_2_Cl_2_) to give the title compound as a white solid (0.040 g, 28%). ^1^H NMR (500 MHz, DMSO-*d*_6_) δ: 8.66 (1H, br s, NH), 8.11 (2H, br s, NH_2_), 7.22–7.12 (1H, m, 2 × ArH), 7.06 (1H, d, *J* = 7.6 Hz, NH), 3.62 (2H, br s, CH_2_), 3.20–3.08 (1H, m, CH), 2.94 (2H, d, *J* = 6.4 Hz, CH_2_), 2.32 (6H, br s, 2 × CH_3_), 2.16–2.09 (1H, m, CH), 2.02–1.90 (4H, m, 4 × C*H*H), 1.40–1.29 (4H, m, 4 × C*H*H), 1.07 (6H, d, *J* = 6.7 Hz, 2 × CH_3_). Note that one CH peak is not observed. ^19^F{^1^H} NMR (470 MHz, DMSO-*d*_6_) δ: −113.61. ^13^C NMR (125 MHz, DMSO-*d*_6_) δ: 170.9, 166.4, 158.4 (dd, *J* = 246, 9.2 Hz, CF), 134.5, 120.7, 115.4–115.2 (m), 60.5, 58.6, 51.3, 42.3, 32.7, 31.0, 24.9, 22.8. Note that one CH and two quaternary carbons are not observed. Note that some quaternary carbon peaks are not strong enough to observe the expected multiplicity. MS (ES+) (%): 530 (100) [M + H]^+^. HRMS (ES+): calcd. for C_23_H_34_F_2_N_5_O_3_S_2_ [M + H]^+^, 530.2066; found, 530.2062 (0.7 ppm).

##### *N*-((*trans*)-4-((4-Amino-5-(2,6-difluoro-4-((4-methylpiperazin-1-yl)methyl)benzoyl)thiazol-2-yl)amino)cyclohexyl)-2-methylpropane-1-sulfonamide Hydrochloride (**3**)

Cyanamide (0.072 g, 1.7 mmol) in MeCN (0.5 mL), potassium *tert*-butoxide (1 M in THF, 2.8 mL), **17** (0.393 g, 1.42 mmol) in MeCN/^*t*^BuOH (1:1, 1.0 mL), and **22b** (0.609 g, 1.42 mmol) were reacted according to general method D. The reaction was then diluted with CH_2_Cl_2_, washed with water, and evaporated under reduced pressure. The resulting crude was purified by silica column chromatography (1–20% MeOH in CH_2_Cl_2_). Treatment with HCl (1.0 M in diethyl ether) gave the title compound as a white solid (0.030 g, 9%). ^1^H NMR (500 MHz, DMSO-*d*_6_) δ: 11.38 (1H, br s, NH^+^), 8.81 (1H, br s, NH), 8.09 (2H, br s, NH_2_), 7.51–7.42 (2H, m, 2 × ArH), 7.02 (1H, d, *J* = 7.0 Hz, NH), 4.74–4.26 (8H, m, 4 × CH_2_), 3.57 (2H, s, CH_2_), 3.13–3.05 (1H, m, CH), 2.89 (2H, d, *J* = 6.4 Hz, CH_2_), 2.81 (3H, s, CH_3_), 2.12–2.04 (1H, m, CH), 1.97–1.86 (4H, m, 4 × C*H*H), 1.37–1.24 (4H, m, 4 × C*H*H), 1.02 (6H, d, *J* = 6.8 Hz, 2 × CH_3_). Note that one CH peak is not observed. MS (ES+) (%): 293 (20) [M + 2H]^2+^, 585 (100) [M + H]^+^.

##### *N*-((trans)-4-((4-Amino-5-(2,6-difluoro-4-((4-morpholinomethyl)benzoyl)thiazol-2-yl)amino)cyclohexyl))-2-methylpropane-1-sulfonamide Hydrochloride (**4**)

Cyanamide (0.030 g, 0.72 mmol) in MeCN (0.5 mL), potassium *tert*-butoxide (1 M in THF, 1.2 mL), **17** (0.166 g, 0.602 mmol) in MeCN/^*t*^BuOH (1:1, 1.0 mL), and **22c** (0.250 g, 0.602 mmol) were reacted according to general method D. The reaction was then diluted with CH_2_Cl_2_, washed with water, and evaporated under reduced pressure. The resulting crude was purified by silica column chromatography (1–20% MeOH in CH_2_Cl_2_). Treatment with HCl (1.0 M in diethyl ether) gave the title compound as a white solid (0.030 g, 7%). ^1^H NMR (500 MHz, DMSO-*d*_6_) δ: 11.00 (1H, br s, NH^+^), 8.73 (1H, br s, NH), 8.25–8.01 (2H, m, NH_2_), 7.52–7.43 (2H, m, 2 × ArH), 7.05 (1H, d, *J* = 7.6 Hz, NH), 4.38 (2H, s, CH_2_), 4.00–3.91 (2H, m, 2 × C*H*H), 3.82–3.57 (3H, m, 2 × C*H*H & CH), 3.33–3.24 (2H, m, 2 × C*H*H), 3.16–3.01 (2H, m, 2 × C*H*H), 2.89 (2H, d, *J* = 6.4 Hz, CH_2_), 2.10–2.03 (1H, m, CH), 1.97–1.83 (4H, m, 4 × C*H*H), 1.36–1.22 (4H, m, 4 × C*H*H), 1.02 (6H, d, *J* = 6.7 Hz, 2 × CH_3_). Note that one CH peak is not observed. MS (ES+) (%): 572 (100) [M + H]^+^. HRMS (ES+): calcd. for C_25_H_36_F_2_N_5_O_4_S_2_ [M + H]^+^, 572.2171; found, 572.2176 (−0.8 ppm).

##### *N*-((*trans*)-4-((-4-Amino-5-(2,6-difluoro-4-(pyrrolidin-1-ylmethyl)benzoyl)thiazol-2-yl)amino)cyclohexyl)-2-methyl-propane-1-sulfonamide Hydrochloride (**5**)

Cyanamide (0.017 g, 0.41 mmol) in MeCN (0.5 mL), potassium *tert*-butoxide (1 M in THF, 0.68 mL), **17** (0.094 g, 0.338 mmol) in MeCN/^*t*^BuOH (1:1, 1.0 mL), and **22d** (0.135 g, 0.338 mmol) were reacted according to general method D. The reaction was then diluted with CH_2_Cl_2_, washed with water, and evaporated under reduced pressure. The resulting crude was purified by silica column chromatography (1–20% MeOH in CH_2_Cl_2_). Treatment with HCl (1.0 M in diethyl ether) gave the title compound as an orange solid (0.025 g, 11%). ^1^H NMR (500 MHz, DMSO-*d*_6_) δ: 10.91 (1H, br s, NH^+^), 8.78 (1H, br s, NH), 8.30–8.01 (2H, m, NH_2_), 7.51–7.47 (2H, m, 2 × ArH), 7.05 (1H, d, *J* = 7.5 Hz, NH), 4.39 (2H, d, *J* = 5.8 Hz, CH_2_), 3.44–3.35 (2H, m, 2 × C*H*H), 3.13–3.00 (3H, m, 2 × C*H*H & CH), 2.89 (2H, d, *J* = 6.4 Hz, CH_2_), 2.11–1.82 (9H, m, CH, 4 × C*H*H & 4 × C*H*H), 1.36–1.23 (4H, m, 4 × C*H*H), 1.02 (6H, d, *J* = 6.7 Hz, 2 × CH_3_). Note that one CH peak is not observed. MS (ES+) (%): 556 (100) [M + H]^+^.

##### *N*-(((trans)-4-((4-Amino-5-(4-azetidin-1-ylmethyl)-2,6-difluorobenzoyl)thiazol-2-yl)amino)cyclohexyl)-2-methylpropane-1-sulfonamide Hydrochloride (**6**)

Cyanamide (0.040 g, 0.96 mmol) in MeCN (1.0 mL), potassium *tert*-butoxide (1 M in THF, 1.20 mL), **17** (0.220 g, 0.80 mmol) in MeCN/^*t*^BuOH (1:1, 2.0 mL), and **22e** (0.307 g, 0.80 mmol) were reacted according to general method D. The reaction was then diluted with CH_2_Cl_2_, washed with water, and evaporated under reduced pressure. The resulting crude was purified by reversed-phase preparative HPLC [water (+0.05% HCOOH v/v):MeCN]. The resultant formate salt was neutralized to the free base followed by treatment with HCl (1.0 M in diethyl ether), which gave the title compound as a solid (0.051 g, 11%). ^1^H NMR (500 MHz, DMSO-*d*_6_) δ: 11.08–11.02 (1H, m, NH^+^), 8.78 (1H, br s, NH), 8.13 (2H, br s, NH_2_), 7.41–7.36 (2H, m, 2 × ArH), 7.03 (1H, d, *J* = 7.7 Hz, NH), 4.40 (2H, d, *J* = 6.4 Hz, CH_2_), 4.11–4.00 (4H, m, 2 × CH_2_), 3.73–3.57 (1H, m, CH), 3.15–3.01 (1H, m, CH), 2.89 (2H, d, *J* = 6.4 Hz, CH_2_), 2.44–2.28 (2H, m, CH_2_), 2.11–2.04 (1H, m, CH), 1.97–1.86 (4H, m, 4 × C*H*H), 1.36–1.24 (4H, m, 4 × C*H*H), 1.02 (6H, d, *J* = 6.7 Hz, 2 × CH_3_). MS (ES+) (%): 542 (100) [M + H]^+^. HRMS (ES+): calcd. for C_24_H_34_F_2_N_5_O_3_S_2_ [M + H]^+^, 542.2066; found, 542.2073 (−1.3 ppm).

##### *N*-((*trans*)-4-((4-Amino-5-(4-(dimethylaminomethyl)-2,6-dichlorobenzoyl)thiazol-2-yl)amino)cyclohexyl)-2-methylpropane-1-sulfonamide Hydrochloride (**11**)

Cyanamide (0.090 g, 2.15 mmol) in MeCN (1.0 mL), potassium *tert*-butoxide (1 M in THF, 2.33 mL), **17** (0.495 g, 1.79 mmol) in MeCN/^*t*^BuOH (1:1, 2.0 mL), and **22f** (0.727 g, 1.79 mmol) were reacted according to general method D. The reaction was then partitioned between CH_2_Cl_2_ and water, the layers separated, and the aqueous layer evaporated under reduced pressure. The resulting crude product was loaded onto an SCX cartridge, eluted with NH_3_ in MeOH:CH_2_Cl_2_, and purified by silica column chromatography (0–6% MeOH in CH_2_Cl_2_). Treatment with HCl (1.0 M in diethyl ether) gave the title compound as a solid (0.035 g, 3%). ^1^H NMR (400 MHz, DMSO-*d*_6_) δ: 10.68 (1H, br s, NH^+^), 8.74 (1H, br s, NH), 8.02 (2H, br s, NH_2_), 7.78 (2H, s, 2 × ArH), 7.03 (1H, d, *J* = 7.8 Hz, NH), 4.30 (2H, d, *J* = 5.3 Hz, CH_2_), 3.14–3.01 (1H, m, CH), 2.89 (2H, d, *J* = 6.4 Hz, CH_2_), 2.73 (6H, d, *J* = 4.7 Hz, 2 × CH_3_), 2.12–2.02 (1H, m, CH), 1.99–1.82 (4H, m, 4 × C*H*H), 1.36–1.23 (4H, m, 4 × C*H*H), 1.02 (6H, d, *J* = 6.7 Hz, 2 × CH_3_). Note that one CH peak is not observed. MS (ES+) (%): 562 (100) [^35^Cl_2_ M + H]^+^, 564 (76) [^35^Cl ^37^Cl M + H]^+^. HRMS (ES+): calcd. for C_23_H_34_Cl_2_N_5_O_3_S_2_ [M + H]^+^, 562.1475; found, 562.1456 (3.3 ppm).

##### *N*-(((*trans*)-4-((4-Amino-5-(4-(2-dimethylamino)ethyl)-2,6-difluorobenzoyl)thiazol-2-yl)amino)cyclohexyl)-2-methylpropane-1-sulfonamide Hydrochloride (**7**)

Cyanamide (0.043 g, 1.02 mmol) in MeCN (1.0 mL), potassium *tert*-butoxide (1 M in THF, 1.10 mL), **17** (0.234 g, 0.85 mmol) in MeCN/^*t*^BuOH (1:1, 2.0 mL), and **22g** (0.328 g, 0.847 mmol) were reacted according to general method D. The resulting precipitate was recovered by filtration, converted to the free base by treatment with Na_2_CO_3_ (5% w/v, aq), extracted into CH_2_Cl_2_, dried, and the solvent was evaporated under reduced pressure. Treatment with HCl (1.0 M in diethyl ether) gave the title compound as a yellow solid (0.152 g, 30%). ^1^H NMR (500 MHz, DMSO-*d*_6_) δ: 10.19 (1H, br s, NH^+^), 8.73 (1H, br s, NH), 8.08 (2H, br s, NH_2_), 7.18–7.14 (2H, m, 2 × ArH), 7.02 (1H, d, *J* = 7.4 Hz, NH), 3.39–3.33 (2H, m, CH_2_), 3.11–3.04 (3H, m, CH & CH_2_), 2.89 (2H, d, *J* = 6.3 Hz, CH_2_), 2.80 (6H, d, *J* = 4.8 Hz, 2 × CH_3_), 2.10–2.05 (1H, m, CH), 1.96–1.85 (4H, m, 4 × C*H*H), 1.36–1.24 (4H, m, 4 × C*H*H), 1.02 (6H, d, *J* = 6.7 Hz, 2 × CH_3_). Note that one CH peak is not observed. MS (ES+) (%): 544 (100) [M + H]^+^. HRMS (ES+): calcd. for C_24_H_36_F_2_N_5_O_3_S_2_ [M + H]^+^, 544.2222; found, 544.2194 (5.1 ppm).

##### *N*-((*trans*)-4-((4-Amino-5-(2,6-difluoro-4-((methylamino)methyl)benzoylthiazol-2-yl)amino)cyclohexyl))-2-methylpropane-1-sulfonamide Hydrochloride (**8**)

Neat 1-chloroethyl chloroformate (0.135 g, 0.94 mmol) was added to a suspension of **29** (0.5 g, 0.79 mmol) and potassium carbonate (0.218 g, 1.57 mmol) in CH_2_Cl_2_ (5 mL), and the reaction was stirred at room temperature for 12 h. CH_2_Cl_2_ was then removed under reduced pressure, the residue re-dissolved in MeOH (5 mL) and was heated at 65 °C for 2 h. The reaction was then cooled, diluted with CH_2_Cl_2_, washed with water, dried over MgSO_4_, filtered, and the solvent was removed under reduced pressure. The crude product was purified by preparative HPLC [water (+0.05% NH_4_OH v/v):MeCN] to give a gum. Treatment with HCl (4.0 M in dioxane) gave the title compound as a yellow solid (0.115 g, 26%). ^1^H NMR (400 MHz, CD_3_OD) δ: 7.05–7.00 (m, 2H, 2 × ArH), 3.74 (s, 2H, CH_2_), 3.22–3.15 (m, 1H, CH), 2.92 (d, *J* = 6.4 Hz, 2H, CH_2_), 2.38 (s, 3H, NCH_3_), 2.24–2.14 (m, 1H, CH), 2.11–2.00 (m, 4H, 4 × C*H*H), 1.44–1.34 (m, 4H, 4 × C*H*H), 1.08 (d, *J* = 6.8 Hz, 6H, 2 × CH_3_). Note that one CH peak is not observed. ^19^F{^1^H} NMR (470 MHz, CD_3_OD) δ: −115.4 (CF). ^13^C NMR (100 MHz, CD_3_OD) δ: 172.6 (C), 166.9 (C), 158.9 (dd, *J* = 247, 8.6 Hz, CF), 144.1 (t, *J* = 8.6 Hz, C), 117.9 (t, *J* = 23.6 Hz, C), 111.3–111.0 (m, CH), 60.8 (CH_2_), 53.8 (CH_2_), 51.5 (CH), 34.0 (CH_3_), 32.5 (CH_2_), 30.7 (CH_2_), 24.8 (CH), 21.5 (CH_3_). Note that one CH and two quaternary carbon peaks are not observed. MS (ES+) (%): 516 (100) [M + H]^+^. HRMS (ES+): calcd. for C_22_H_32_F_2_N_5_O_3_S_2_ [M + H]^+^, 516.1909; found, 516.1891 (3.5 ppm).

##### *N*-((*trans*)-4-((4-Amino-5-(2,6-dichlorobenzoyl)thiazol-2-yl)amino)cyclohexyl)-2-methylpropane-1-sulfonamide (**10**)

Cyanamide (0.142 g, 3.37 mmol) in MeCN (3.0 mL), potassium *tert*-butoxide (1 M in THF, 3.37 mL), **17** (0.777 g, 2.81 mmol) in MeCN/^*t*^BuOH (1:1, 4.0 mL), and 2-bromo-1-(2,6-dichlorophenyl)ethanone (**22i**) (0.753 g, 2.81 mmol) were reacted according to general method D. The resulting crude was directly purified by silica column chromatography (20–100% EtOAc in hexane) to give the title compound as a green solid (1.02 g, 72%). ^1^H NMR (500 MHz, DMSO-*d*_6_) δ: 8.59 (1H, br s, NH), 7.98 (2H, br s, NH_2_), 7.52–7.50 (2H, m, 2 × ArH), 7.42 (1H, dd, *J* = 8.8, 7.4 Hz, ArH), 7.04 (1H, d, *J* = 7.6 Hz, NH), 3.12–3.01 (1H, m, CH), 2.89 (2H, d, *J* = 6.4 Hz, CH_2_), 2.11–2.03 (1H, m, CH), 1.97–1.84 (4H, m, 4 × C*H*H), 1.34–1.25 (4H, m, 4 × C*H*H), 1.02 (6H, d, *J* = 6.7 Hz, 2 × CH_3_). Note that one CH proton was not observed. HRMS (ES+): calcd. for C_20_H_26_Cl_2_N_4_O_3_S_2_ [M + H]^+^, 505.0896; found, 505.0898 (−0.4 ppm).

##### *N*-((*trans*)-4-((4-Amino-5-(2,6-difluorobenzoyl)thiazol-2-yl)amino)cyclohexyl)-2-methylpropane-1-sulfonamide (**1**)

Cyanamide (0.042 g, 1.0 mmol), potassium *tert*-butoxide (1 M in THF, 1.0 mL), **17** (0.276 g, 1.0 mmol), and 2-bromo-1-(2,6-difluorophenyl)ethanone (**22j**) (0.282 g, 1.2 mmol) were reacted according to general method D to give the title compound as a white solid (0.255 g, 54%). ^1^H NMR (500 MHz, DMSO-*d*_6_) δ: 8.64 (1H, br s, NH), 8.07 (2H, br s, NH_2_), 7.53–7.47 (1H, m, ArH), 7.19–7.15 (2H, m, 2 × ArH), 7.04 (1H, d, *J* = 7.6 Hz, NH), 3.12–3.01 (1H, m, CH), 2.89 (2H, d, *J* = 6.4 Hz, CH_2_), 2.10–2.03 (1H, m, CH), 1.97–1.84 (4H, m, 4 × C*H*H), 1.34–1.23 (4H, m, 4 × C*H*H), 1.02 (6H, d, *J* = 6.7 Hz, 2 × CH_3_). Note that one CH peak is not observed. ^13^C NMR (125 MHz, DMSO-*d*_6_) δ: 171.9, 166.2, 158.7 (dd, *J* = 246, 8.2 Hz, CF), 131.7 (t, *J* = 9.9 Hz), 120.1 (t, *J* = 24 Hz), 112.6–112.4 (m), 60.5, 51.3, 32.7, 31.1, 24.9, 22.8. Note that one aliphatic and two aromatic carbon peaks are not observed. MS (ES+) (%): 473 (100) [M + H]^+^. HRMS (ES+): calcd. for C_20_H_26_F_2_N_4_O_3_S_2_ [M + H]^+^, 473.1487; found, 473.1521 (−7.2 ppm).

##### *tert*-Butyl (2-(2-((4-(4-Amino-2-((*trans*-4-((2-methylpropyl)sulfonamido)cyclohexyl)amino)thiazole-5-carbonyl)-3,5-difluorophenyl)(methyl)amino)ethoxy)ethyl)carbamate (**38**)

A solution of tosylate^12^**37** (79 mg, 0.22 mmol) in anhydrous MeCN (1 mL) was added to a suspension of K_2_CO_3_ (30 mg, 0.22 mmol) and **8** (103 mg, 0.2 mmol) in MeCN (1 mL), and the resultant mixture was heated at 82 °C for 16 h. The reaction solvent was then removed using a stream of nitrogen, and the crude product was purified directly by reversed-phase HPLC (5–95% MeCN in water+0.1% NH_3_) followed by silica column chromatography (12 g silica, 0–10% EtOH in EtOAc) to give the title compound as a white solid (31 mg, 18%). Rf (silica, 10:90 EtOH:EtOAc) 0.5. ^1^H NMR (500 MHz, DMSO-*d*_6_) δ: 8.60 (br s, 1H, NH), 8.02 (s, 2H, NH_2_), 7.08–7.07 (m, 2H, 2 × ArH), 6.97 (d, *J* = 7.5 Hz, 1H, NH), 6.76–6.71 (m, 1H, NH), 3.59–3.52 (m, 5H, 2 × CH_2_ & CH), 3.38 (t, *J* = 6 Hz, 2H, CH_2_), 3.09–3.06 (m, 3H, CH_2_ & CH), 2.88 (d, *J* = 6.5 Hz, CH_2_), 2.59–2.53 (m, 2H, CH_2_), 2.20 (s, 3H, CH_3_), 2.11–2.03 (m, 1H, CH), 1.97–1.84 (m, 4H, 4 × C*H*H), 1.38–1.24 (m, 9H, ^t^Bu & 4 × C*H*H), 1.01 (d, *J* = 6.5 Hz, 6H, 2 × CH_3_). ^19^F{^1^H} NMR (470 MHz, DMSO-*d*_6_) δ: −114.8. ^13^C NMR (125 MHz, DMSO-*d*_6_) δ: 172.0, 166.1, 158.5 (dd, *J* = 245, 9 Hz, CF), 156.1, 112.0 (d, *J* = 21 Hz), 78.0, 69.5, 68.8, 60.8, 60.5, 56.5, 51.3, 42.7, 32.8, 31.1, 28.7, 24.9, 22.8. Note that four quaternary carbon and one CH peak are not observed, and one methylene peak is obscured by the DMSO-*d*_6_ multiplet. MS (ES+) (%): 603 (40) [M–Boc + H]^+^, 703 (100) [M + H]^+^. HRMS (ES+): calcd. for C_31_H_48_F_2_N_6_O_6_S_2_ [M + H]^+^, 703.3118; found, 703.3130 (1.7 ppm).

##### *N*-((*trans*)-4-((4-Amino-5-(4-(((2-(2-aminoethoxy)ethyl)(methyl)amino)methyl)-2,6-difluorobenzoyl)thiazol-2-yl)amino)cyclohexyl)-2-methylpropane-1-sulfonamide Trifluoroacetate Salt (**13**)

Neat TFA (0.1 mL) was added to a solution of carbamate **38** (7.5 mg, 10.7 μmol) in anhydrous CH_2_Cl_2_ (1.9 mL) at 0 °C. The reaction was allowed to warm to room temperature with stirring over 2.5 h. The reaction was then evaporated to dryness under vacuum, and the resultant salt was used without further purification (11 mg, quant.). ^1^H NMR (500 MHz, CD_3_OD) δ: 7.30–7.28 (m, 2H, 2 × ArH), 4.47 (s, 2H, CH_2_), 3.90–3.88 (m, 2H, CH_2_), 3.76–3.74 (m, 2H, CH_2_), 3.44–3.40 (m, 2H, CH_2_), 3.22–3.17 (m, 3H, CH_2_ & CH), 2.94–2.92 (m, 5H, CH_3_ & CH_2_), 2.23–2.15 (m, 1H, CH), 2.11–2.01 (m, 4H, 4 × C*H*H), 1.43–1.36 (m, 4H, 4 × C*H*H), 1.09 (d, *J* = 7.0 Hz, 6H, 2 × CH_3_). Note that one of the CH peaks is not visible, probably due to the broadening of peaks corresponding to the cyclohexyl moiety. ^19^F{^1^H} NMR (470 MHz, CD_3_OD) δ: −77.3 (s, 9F, 3 × CF_3_), −113.0 (s, 2F, 2 × CF). MS (ES+) (%): 302 (45) [M + 2H]^2+^, 603 (100) [M + H]^+^. HRMS (ES+): calcd. for C_26_H_41_F_2_N_6_O_4_S_2_ [M + H]^+^, 603.2593; found, 603.2568 (4.2 ppm).

#### Preparation of Resin Bound Compound **13**

Ten milligrams of beads derivatized with compound **13** were prepared as follows. The DMAc storage solvent was removed from commercial NHS-ester-functionalized magnetic beads (Thermo Scientific), and the beads were washed and resuspended in anhydrous DMSO (100 μL [mg resin]^−1^). The DMSO was then removed and replaced with a DMSO solution (100 μL [mg resin]^−1^) containing amine **13** (7 nmol [mg resin]^−1^) and DIPEA (28 nmol [mg resin]^−1^), and the resin was gently agitated for 24 h at room temperature. The reaction solvent was subsequently removed, and the beads again were washed with and resuspended in anhydrous DMSO (100 μL [mg resin]^−1^). Unreacted sites on the resin were then “blocked” by incubating the resin with a DMSO solution (100 μL [mg resin]^−1^) containing ethanolamine (70 nmol [mg resin]^−1^) and DIPEA (70 nmol [mg resin]^−1^) with gentle agitation for 24 h at room temperature. The reaction solvent was then removed, and the resin was washed with DMAc (100 μL [mg resin]^−1^), prior to storage in the same solvent.

## Supporting Information Available

Detailed materials and methods including the followign: the *in vitro* and *in vivo* parasite assays, the *in vitro* and *in vivo* drug metabolism and pharmacokinetic assays, formulation and salt formation experiments, details of the molecular modeling; mode of action methods;(Figures S1–S9 and Tables S1–15) (PDF)
Molecular formula strings for key molecules (PDB).
Coordinates for the homology model of *T. brucei* CRK12 with compound **2** docked into the ATP site, as shown in Figure 5 (CSV)

## Supplementary Material

jm1c02104_si_001.pdf

jm1c02104_si_002.pdb

## Notes

The mass spectrometry proteomics data have been deposited to the ProteomeXchange Consortium via the PRIDE partner repository with the dataset identifier PXD023089.^22^

## Abbreviations

AAT: African animal trypanosomiasis
AUC: area under the curve
*C*_lb_: clearance in blood
*C*_li_: intrinsic clearance
*C*_max_: the maximum concentration detected in blood
CNS: central nervous system
CRK12: cyclin-dependent related kinase 12
F: oral bioavailability
HAT: human African trypanosomiasis
IM: intramuscular
IV: intravenous
MTD: maxium tolerated dose
PK: pharmacokinetics
SC: subcutaneous
*T*_max_: the time after dosing that the concentration in blood is the highest
*V*_dss_: the volume of distribution
