# Supplementary material for: Repositioning of a Diaminothiazole Series Confirmed to Target the Cyclin-Dependent Kinase CRK12 for Use in the Treatment of African Animal Trypanosomiasis
Source: J Med Chem. 2022 Mar 18;65(7):5606–24. doi: 10.1021/acs.jmedchem.1c02104 (PMC9014415; doi:10.1021/acs.jmedchem.1c02104)
Supplement: Supplementary file 1 — jm1c02104_si_001.pdf [file jm1c02104_si_001.pdf]

SUPPORTING INFORMATION

Repositioning of a diaminothiazole series confirmed to target the cyclin-dependent kinase CRK12 for use in the treatment of African animal trypanosomiasis

Alasdair Smith[1#], Richard J. Wall[2#], Stephen Patterson[2#], Tim Rowan[3], Eva Rico Vidal[2], Laste Stojanovski[1], Margaret Huggett[1], Shahienaz E. Hampton[1], Michael G. Thomas[1], Victoriano Corpas Lopez[2], Kirsten Gillingwater[4,5], Jeff Duke[6], Grant Napier[3], Rose Peter[3], Hervé S. Vitouley[7], Justin R. Harrison[1], Rachel Milne[2], Laura Jeacock[2], Nicola Baker[2], Susan H. Davis[1], Frederick Simeons[1], Jennifer Riley[1], David Horn[2], Reto Brun[4,5], Fabio Zuccotto[1], Michael J Witty[3], Susan Wyllie[2]*, Kevin D. Read[1]*, Ian H. Gilbert[1]*

[1]Drug Discovery Unit, Wellcome Centre for Anti-Infectives Research, Division of Biological Chemistry and Drug Discovery, University of Dundee, Dow Street, Dundee DD1 5EH, United Kingdom.

[2]Wellcome Centre for Anti-Infectives Research, Division of Biological Chemistry and Drug Discovery, School of Life Sciences, University of Dundee, Dow Street, Dundee DD1 5EH, United Kingdom.

[3]GALVmed, Doherty Building, Pentlands Science Park, Bush Loan, Penicuik, Edinburgh, EH26 0PZ, United Kingdom

[4]Swiss Tropical and Public Health Institute, Socinstrasse 57, CH-4002 Basel, Switzerland.

[5]University of Basel, Petersplatz 1, CH-4001 Basel, Switzerland

[6]University of Greenwich, Medway Campus, Central Avenue, Chatham Maritime, Chatham, Kent ME4 4TB United Kingdom

[7]Centre International de Recherche-Développement sur l'Elevage en zone Subhumide (CIRDES) No 559, ru 5-31 angle Av. du Gouverneur Louveau. 01 BP: 454 Bobo-Dioulasso 01, Burkina Faso

*Corresponding authors: Ian Gilbert (i.h.gilbert@dundee.ac.uk); Kevin Read (k.read@dundee.ac.uk); Susan Wyllie (s.wyllie@dundee.ac.uk)

[#]Authors contributed equally

CONTENTS

1. Parasitology 3
1.1 *In vitro* drug sensitivity assays - *Trypanosoma brucei brucei* 3
1.2 *In vitro* drug sensitivity assays - *Trypanosoma congolense* 3
1.3 *Ex vivo* drug sensitivity assays - *Trypanosoma vivax* 3
1.4 Cytotoxicity assays – Rat skeletal myoblasts (L6) 4
2. Drug metabolism and pharmacokinetics 4
2.1 Solubility 4
2.2 Microsomal stability studies 6
2.3 Hepatocyte stability studies 6
2.4 Protein plasma binding 8
2.5 PK and maximum tolerated dose - mouse 8
2.6 PK and formulation studies - male Sprague rats 8
2.7 Pharmacokinetic studies in cattle (see Table 2). Studies conducted at Ridgeway 9
2.8 Formulation and tolerability studies for compound 2 (NorthWest Biopharm) 9
2.9 Salt screen 12
3. *In vivo* efficacy studies 13
3.1 Mouse efficacy studies for *T. brucei brucei.* 13
3.2 NMRI mouse efficacy studies for *T. congolense* and *T. vivax.* 13
3.3 Cattle efficacy for compound 1 (Clinvet) 14
3.4 Cattle efficacy for compound 2, intravenous infusion (Clinvet) 15
3.5 Cattle efficacy for compound 2, intramuscular (CIRDES) 15
4. Mode of action studies 16
4.1 Resistant line generation 16
4.2 Whole genome sequencing and analysis 16
4.3 RIT-seq screening, Illumina sequencing and analysis 17
4.4 SILAC pulldown 18
4.5 Peptide fractionation and LC-MS/MS analysis 19
4.6 Data analysis 20
5. Molecular modelling 20
6. Supplementary figures 21
7. LCMS chromatograms for compounds used in in vivo studies 27
8. Supplementary tables 34
9. References 47

1. Parasitology

1.1 *In vitro* drug sensitivity assays - *Trypanosoma brucei brucei*

Drug sensitivity assays were carried out with bloodstream-form (BSF) *T. b. brucei* 'single marker' S427 cells grown at 37°C with 5% CO_2 in HMI-9T medium,[1] as previously described.[2]

1.2 *In vitro* drug sensitivity assays - *Trypanosoma congolense*

EC_{50} values for test compounds against *T. congolense* (IL3000 strain) were conducted as previously described[3] with minor modifications.[4] Briefly, trypanosome cell densities were determined using a CASY cell counter (Schärfe) and diluted to a seeding density of 2 x 10^5 trypanosomes/mL. BSF trypanosomes were grown in HMI-9 (IMDM) media supplemented with 20% (v/v) bovine serum.[1] Test compounds were resuspended to a final concentration of 10 mg/mL in DMSO. Assays plates were incubated at 34 °C with 5 % CO_2, for a total of 69 h, prior to addition of 10 µL of Resazurin (12.5 mg in 100 mL phosphate buffered saline, Aldrich) into each well, followed by a further 3 h incubation. Absorbance was read using a SpectraMax (Gemini XS) fluorescence reader at an excitation wavelength of 536 nm and an emission wavelength of 588 nm. EC_{50} values were determined using SOFTmax Pro 5.2 analysis software. All experiments were conducted with technical duplicates, and at least three biological replicates.

1.3 *Ex vivo* drug sensitivity assays - *Trypanosoma vivax*

Since *T. vivax* cannot be cultured axenically, BSF trypanosomes were harvested from an infected mouse with high parasitemia via cardiac puncture and used directly in *ex vivo* assays. EC_{50} values were determined against *T, vivax* (STIB 719/ ILRAD 560) as previously described[3], with minor modifications. Briefly, cell densities were determined using a Neubauer chamber and then diluted to a seeding density of 4 x 10^5 trypanosomes / mL. BSF trypanosomes were cultured in HMI-9 (IMDM) media containing 20% (v/v) bovine serum. Assay plates were incubated at 37 °C with 5 % CO_2, for a total of 45 h prior to addition 10 µL of Resazurin (12.5 mg in 100 mL phosphate buffered saline, Aldrich) into each well, followed by a further 3 h incubation. Absorbance was read using a SpectraMax (Gemini XS) fluorescence reader at an excitation wavelength of 536 nm and an emission wavelength of 588 nm. EC_{50} values were determined using SOFTmax Pro 5.2 analysis

software. All experiments were conducted with technical duplicates and at least three biological replicates.

1.4 Cytotoxicity assays – Rat skeletal myoblasts (L6)

The cytotoxicity of test compounds was evaluated using a rat skeletal myoblast (L6) cell line, as previously described[3], but with minor modifications. Cells were grown in RPMI media supplemented with 10% (v/v) fetal calf serum. L6 cells at a seeding density of 2 x 10^4 cells/mL were incubated in microtiter plates overnight at 37 ºC and 5 % CO_2 until adherent. The following day, test compounds were added to the plates (3-fold serial dilutions), prior to incubation for 69 h. Following incubation, 10 µL of Resazurin (12.5 mg in 100 mL phosphate buffered saline, Aldrich) was added to each well and the plates were incubated for a further 3 h. The plates were read using a SpectraMax (Gemini XS) fluorescence reader at an excitation wavelength of 536 nm and an emission wavelength of 588 nm. EC_{50} values were determined using SOFTmax Pro 5.2 analysis software. All experiments were conducted as technical replicates, with at least three biological replicates.

2. Drug metabolism and pharmacokinetics

2.1 Solubility

The aqueous solubility of test compounds was measured using laser nephelometry. Compounds were subject to serial dilution from 10 mM to 0.5 mM in DMSO. Aliquot were then mixed with MilliQ water to obtain an aqueous dilution plate with a final concentration range of 250 – 12 µM. The final DMSO concentration in these plates was 2.5%. Triplicate aliquots were transferred to a flat-bottomed polystyrene plate which was immediately read on a NEPHELOstar nephelometer (BMG Lab Technologies). The laser scatter caused by insoluble particulates (relative nephelometry units, RNU) was plotted against compound concentration using a segmental regression fit, with the point of inflection being quoted as the compound's aqueous solubility (µM).

Solubility of compound **1** was measured by a different method. Here, compound **1** was dissolved in DMSO to give a 10 mM solution and solubility test samples prepared by adding a volume (5 µL) of this stock solution to phosphate buffered saline, pH 7.4 (195 µL, Sigma-Aldrich). This

solution was then mixed for 24 h (rotary mixing, 900 rpm, 25°C) excluding light. After mixing, test samples were filtered to remove any undissolved material using a proprietary filter (Millipore Multiscreen HTS filter, 96-well format). Samples were drawn through the filter using a vacuum. The resulting filtrate was analysed for dissolved compound 1 using a truncated UHPLC methodology. A Shimadzu Nexera X2 UHPLC system was used, with a reversed-phase column and a simple formic acid gradient elution. The UHPLC parameters are shown below (Table S1):

Table S1: UHPLC Parameters

Parameter	Value
**Mobile phase component A**	HPLC water plus 0.1% formic acid
**Mobile phase component B**	HPLC acetonitrile plus 0.1% formic acid
**Flow rate:**	0.6 mL/min
**Gradient program:**	Initial: 98% A, 2% B<br>At 1.2 min: 2% A, 98% B<br>At 2.0 min: 2% A, 98% B<br>Re-equilibration time: 0.6 min
**Autosampler temperature:**	25°C
**Column:**	Hypersil Gold, C18 1.9 μm, 50 × 2.1 mm
**Column temperature:**	50°C
**Detector wavelength:**	254 nm
**Bandwidth:**	4 nm

A calibration solution was prepared as follows: The same 10 mM solution used to prepare the solubility test sample was diluted in DMSO to give a 500 μM solution. This solution was further diluted with 50:50 acetonitrile:water to give a final 50 μM solution. Aliquots (0.2, 2.0 and 5.0 μL) of this 50 μM solution were injected onto the UHPLC system and the areas of the resultant peaks integrated to produce a calibration line. Aliquots of the test sample filtrate (0.4 and 5.0 μL) were then injected onto the UHPLC system and the resultant peak areas for any peaks corresponding to the test compound determined and quantified using the calibration line. Although this is also a "kinetic"

solubility method,[5] the long shaking times appear to give solubility values that more closely reflect those made from solid samples (unpublished in-house data).

2.2 Microsomal stability studies

(a) *Mouse liver microsome stability*. These methods have been previously reported.[6]

(b) *Bovine liver microsome stability.* Test compounds (0.5 µM) were incubated with 0.5 mg/mL bovine liver microsomes (Xenotech LLC ™) in 50 mM potassium phosphate buffer, pH7.4 and the reaction initiated upon addition of excess NADPH (8 mg/mL in 50 mM potassium phosphate buffer, pH7.4). Aliquots (50 µL) of the incubation mixture were taken at time zero, 3, 6, 9, 15 and 30 min then mixed with acetonitrile (100 µL) to stop the reaction. An internal standard was added to all samples, prior to centrifugation (10 min, 1,665 g, RT) to sediment precipitated protein. Reaction plates were then sealed prior to UPLCMSMS analysis using a Quattro Premier XE (Waters corporation, USA).

XLfit (IDBS, UK) was used to calculate the exponential decay and consequently the rate constant (k) from the ratio of peak area of test compound to internal standard at each timepoint. The rate of intrinsic clearance (CLi) of each test compound was then calculated using the following calculation:

$$CLi(mL/min/g\ liver) = k \times V \times \text{microsomal protein yield}$$

Where V (mL/mg protein) is the incubation volume/ mg protein added and microsomal protein yield is taken as 52.5 mg protein/ g of liver. Verapamil (0.5 µM) was used as a positive control to confirm acceptable assay performance.

2.3 Hepatocyte stability studies

(a) *Mouse hepatocyte stability studies*. Cryopreserved vials of mouse cryopreserved hepatocytes, supplied by Life Technologies, were thawed according to manufacturer's instructions and cells resuspended in Williams Medium E (WME) containing cell maintenance supplement pack (CM4000, Life Technologies). Hepatocytes were incubated in suspension (0.5 million cells/mL) in 48 well non-

collagen coated cell culture plates for 10 minutes at 37°C, 5% CO_2. Upon addition of an equal volume of supplemented WME containing 1 μM test compound, an aliquot of incubation solution was removed to acetonitrile containing internal standard (final concentration 0.5 μM test compound and a cell density of 0.25 million cells/mL). Similarly, aliquots were removed at 3, 6, 9, 15, 30, 45, 60, 90 and 120 min. 100 μL of 80:20 water:acetonitrile was added to all samples and the analysis plate was centrifuged (10 min, 1,665 *g*, RT) prior to injection and analysis of samples by UPLC-MS/MS. The response (area ratio of test compound relative to internal standard) was plotted against time using an exponential decay model and rate of disappearance calculated. Hepatocyte CLint (mL/min/10^6 cells) was scaled to in vivo CLint (ml/min/g Liver) using the hepato-cellularity scaling factor of 120 x 10^6 cells/g of liver.

(b) *Bovine hepatocyte stability.* Freshly isolated female bovine (Prim'Holstein) hepatocytes were supplied by Biopredic International™. Cells were resuspended in Williams Medium E (WME) containing cell maintenance supplement pack (CM4000, Life Technologies). Hepatocytes were incubated in suspension (5×10^5 cells/mL) in 48 well non-collagen coated cell culture plates for 10 min at 37°C. Upon addition of an equal volume of supplemented WME containing 1 μM test compound. Aliquots of incubation solution were removed and added to acetonitrile containing internal standard (final concentration 0.5 μM test compound and a cell density of 7×10^5 cells/mL). Similarly, aliquots were removed at 10, 20, 30, 45, 60, 75, 90, 105 and 120 min. 100 μL of 80:20 water:acetonitrile was added to all samples and the analysis plate was centrifuged (10 min, 1,665 g, RT) prior to injection and analysis of samples by UPLC-MS/MS. The response (area ratio of test compound to internal standard) was plotted against time using an exponential decay model and rate of disappearance calculated. Hepatocyte CLint (mL/min/10^6 cells) was scaled to *in vivo* CLint (mL/min/g liver) using the hepato-cellularity scaling factor of 120 x 10^6 cells/g of liver.

2.4 Protein plasma binding

This was determined as previously described.[6]

2.5 PK and maximum tolerated dose - mouse

PK studies in female NMRI mice (Figure S1): Test compounds **1**, **2** and **10** were dosed SC at 10 mg free base/kg (dose volume 10 mL/kg; dose vehicle: 5% (v/v) DMSO, 40% polyethylene glycol 400 (PEG400) and 55% deionized water) to female NMRI mice (n = 3). Blood samples (10 µL) were taken from each mouse at 0.08, 0.25, 0.5, 1, 2, 4, 6, 8 h post dose (24 h post dose for compounds **2** and **10** only) and mixed with two volumes of deionized water (20 µL). Alternatively, compound **2** was delivered for 48 h via SC implanted ALZET osmotic pumps (10, 25 and 50 mg free base/mL, vehicle: 50% DMSO and 50% deionized water) to the female NMRI mouse (n = 3). Blood samples (10 µL) from each mouse were taken at 1, 3, 6, 24, 30 and 48 h post-dose and mixed with two volumes of deionized water (20 µL).

Maximum tolerated dose

Compound **1** as free base in 5% DMSO; 40% PEG400; 55% MilliQ water was dosed daily subcutaneously with doses of 50 mg/kg, 100 mg/kg and 300 mg/kg for 3 days. No tolerability issues were detected.

2.6 PK and formulation studies - male Sprague rats

Compound **1** was dosed SC at 5 mg free base/kg (dose volume 0.2 or 1 mL/kg; dose vehicle 10% *N*-methyl-2- pyrrolidinone (NMP), 30% 2-pyrrolidinone, 40% PEG400, 20% deionized water or 10% DMSO, 30% PEG400 and 60% deionized water, respectively) or IM at 5 and 10 mg free base/kg (dose volume 0.2 mL/kg; dose vehicle 10% NMP, 30% 2-pyrrolidinone, 40% PEG400, 20% deionized water or 83.4% 2-pyrrolidinone/polyvinylpyrrolidone (PVP), 16.6% saline or 80% 2-pyrrolidinone/NMP/PVP, 20% (0.05% 2-propanol in saline) to male SD rats (n=3).

Compound **2** was dosed IV at 3 mg free base/kg (dose volume 1 mL/kg; dose vehicle: 10% DMSO, 40% PEG400 and 50% deionized water) or IM at 5, 10 and 20 mg free base/kg (dose volume 0.2

and 0.4 mL/kg; dose vehicle 10% NMP, 30% 2-pyrrolidinone, 40% PEG400, 20% deionized water or 83.4% 2-pyrrolidinone/polyvinylpyrrolidone (PVP), 16.6% saline or 80% 2-pyrrolidinone/NMP/PVP, 20% 0.05% 2-propanol in saline or 40% 0.3M Acetate Buffer, 20% Kolliphor, 20% glycerol formal, 20% 2-pyrrolidinone or 40% 0.3M Acetate Buffer, 20% glycerol formal, 20% 2-pyrrolidinone) to male SD rats (n=3). Blood samples (10 µL) were taken from each rat at 0.08, 0.25, 0.5, 1, 2, 4, 6, 8 and 24 h post dose and mixed with two volumes of deionized water (20 µL).

2.7 Pharmacokinetic studies in cattle (see Table 2). Studies conducted at Ridgeway

Compound **1** was dosed IM at 5 mg free base/kg (dose volume 2mL/10 kg; dose vehicle 10% N-Methyl-2-Pyrrolidinone (NMP), 40% 2-pyrrolidinone, 30% PEG400, 20% deionized water) or IV at 2.5 mg free base/kg (dose volume 0.4 mL/10 kg; 2.5% NMP, 30% PEG400, 37.5% propylene glycol, 30% water by volume) to male ruminating Holstein-Friesian cross animals (n = 4). Blood samples were taken from each animal using 18-G needles into EDTA (K2) vacutainers before dosing and at 0.25, 0.5, 1, 2, 4, 8, 12, 24, 48, 72, 96 and 120 h post dose. They were immediately transferred into a cool box containing iced water and were processed within 2 h of collection (Figure **S2**, Table **S1**).

Compound **2** was dosed IM at 10 mg free base/kg (dose volume 2 mL/10 kg; dose vehicle 40% 0.3 M Acetate Buffer, 20% Kolliphor EL, 20% glycerol formal, 20% 2-pyrrolidone) or IV at 2 mg free base/kg (dose volume 0.4 mL/10 kg; 40% 0.3 M Acetate Buffer, 20% Kolliphor EL, 20% glycerol formal, 20% 2-pyrrolidone) to male ruminating Holstein-Friesian cross animals (n = 3). Blood samples were taken from each animal using 18-G needles into EDTA (K2) vacutainers before dosing and at 0.25, 0.5, 1, 2, 4, 8, 12, 24, 48, 72, 96 and 120 h post dose. They were immediately transferred into a cool box containing iced water and were processed within 2 h of collection.

2.8 Formulation and tolerability studies for compound 2 (NorthWest Biopharm)

A study to identify alternative formulations was conducted. Compound **2**, either as the hydrochloride salt or free base, was screened to identify a 50 mg/mL soluble formulation suitable for injection.

Different formulations investigated to reduce injection site tolerability (Table S2)

Table S2: Salt Formulations Investigated

ID	Salt/ Neutral	Formulation
1	HCl	Kolliphor EL, glycerol formal, 2-pyrrolidone and 11% water
2	HCl	Kolliphor EL, *N*-methyl pyrrolidone, 2-pyrrolidone, and 11% water
3	Free base	Kolliphor EL, *N*-methyl pyrrolidone, 2-pyrrolidone and 11% water

The various formulations were then subjected to an injection site tolerability assessment (IST) in cattle to determine if any irritation at the site of injection was evident. Each formulation was administered to two female calves. The calves were cross-breeds (1 x Limousine cross 2 x Hereford cross, 3 x Aberdeen Angus cross). The three formulations of the IVP were supplied formulated ready for administration. Each formulation was administered by single intramuscular injection into the area of the semi-tendinosis muscle of the hind limb. and with ascending IM doses of 1 mL, 5 mL and 10 mL. Saline was used as a control. The site of administration for each formulation on each study day was specified in the table below (Table S3).

Table S3: Sites of Administration for Each Formulation of Compound **2**

Study Day	T01/Animal 1 (Formulation 1)	T02/Animal 2 (Formulation 2)	T03/Animal 3 (Formulation 3)
0	1ml (lower left)	1ml (lower left)	1ml (lower left)
3	5ml (upper left)<br>saline (upper right)	5ml (upper left)<br>saline (upper right)	5ml (upper left)<br>saline (upper right)
7	N/A	10ml (lower right)<br>Saline (lower left)	10ml (lower right)<br>Saline (lower left)
**Study Day**	**T01/Animal 5 (Formulation 1)**	**T02/Animal 6 (Formulation 2)**	**T03/Animal 7 (Formulation 3)**
10	1ml Highest non-reactive dose (lower left)	1ml Highest non-reactive dose (lower left)	1ml Highest non-reactive dose (lower left)
13	5ml Highest non-reactive dose (upper left)<br>Saline (upper right)	5ml Highest non-reactive dose (upper left)<br>Saline (upper right)	5ml Highest non-reactive dose (upper left)<br>Saline (upper right)
17	10ml Highest non-reactive dose (lower right)<br>Saline (lower left)	10ml Highest non-reactive dose (lower right)<br>Saline (lower left)	10ml Highest non-reactive dose (lower right)<br>Saline (lower left)

On the day of administration, prior to administration of test compounds to any animal and at 30 min and 1 h (± 5 min) and at 3 and 5 h (± 10 min) after administration, a whole blood sample was collected from that animal. This sample was used to determine creatinine kinase (CK) levels, a marker of muscle damage, and levels of compound **2** in the animal at each collection timepoint.

Approximately 5 mL of whole blood was collected from the jugular vein using an appropriate needle and syringe. The sample was divided between two 1.3 mL heparinised blood tubes and one 1.3 mL EDTA (K2) tube. Immediately after collection, the EDTA (K2) tube was placed into iced water. Thereafter, 2 x 0.5 mL of this blood was accurately pipetted into two Eppendorf tubes each containing 1.0 mL of distilled water (also accurately pipetted and stored in a refrigerator). These tubes were pre- labelled for each animal and placed in a rack so that the samples, when frozen, remained at the bottom of the tubes. The blood and water were mixed well by pipetting gently up and down. The samples were then placed in a freezer (-15°C to -20°C). One aliquot of each sample was shipped frozen to the University of Dundee, UK for assessing concentration of compound 2. All cattle blood samples were analysed by UPLC-MS/MS on a Xevo™ TQ-S Mass Spectrometer (MSn) (Waters Corporation, USA). The key Pharmacokinetic parameters were calculated using appropriate software (PK solutions, Summit Research Services, USA).

Following completion of the final clinical assessment of the site of administration (at least 72 h after the last IVP administration to that animal), each animal was euthanised by overdose of barbiturate. Barbiturate was administered via the jugular vein. The site of each IVP administration was dissected from the muscle mass in the form of a block of muscle approximately 2 cm^3. Any lesions and their extent at the site of administration were recorded and a digital colour photograph of the lesion was captured and identified with the animal number and the time and date of the photograph. The clinical observations for each formulation are presented below:

- *Formulation 1:* Following a 5 mL injection with formulation 1 (Compound **2** hydrochloride salt in Kolliphor EL, Glycerol Formal, 2-Pyrrolidone and 11% water suitable for injection), an 8 cm swelling appeared at the injection site. Thus, further study of this formulation was stopped prior to proceeding to 10 mL dosing.

- *Formulation 2:* Following a 5 mL injection with formulation 2 (compound **2** hydrochloride salt in Kolliphor EL, *N*-methyl pyrrolidone, 2-pyrrolidone, and 11% water suitable for injection), a 2-4 cm swelling appeared at the injection site, however, no pain or lameness observed. In contrast, no injection site changes were evident following an injection of 10 mL and there were no signs of associated pain or lameness.
- *Formulation 3*: Administration of a 5 mL injection of formulation 3 (compound **2** free base in Kolliphor EL, *N*-methyl pyrrolidone, 2-pyrrolidone and 11% water suitable for injection) resulted in a 1-2 cm swelling and a 10 mL injection caused 4 cm swelling.

It should be noted that swellings of <5 cm in these studies are not considered significant. Creatine kinase levels were also markedly lower with formulations 2 and 3 when compared to those in animals treated with formulation 1.

During the study all calves remained clinically well with only local injection site reactions. Blood concentration at each dose volume for each formulation are summarised in Table S3. In parallel, all the formulations were assessed in rats (IM) and the results were comparable to those in cattle, observationally and with regard to creatine kinase levels. These observations offer confidence that rats can be used as a model animal in the drug development path going forward prior to studies in cattle.

Method (IM)

Comparative cattle PK (Northwestern Biopharm)

The pharmacokinetics for compound **2** when dosed as free base in the best tolerated formulation: Kolliphor EL, *N*-methyl pyrrolidone, 2-pyrrolidone and 11% water at 5 mg/kg was assessed, IM compared to SC. Mean pharmacokinetic parameters are presented in Table S4. Bioanalysis of blood samples was conducted at the University of Dundee.

2.9 Salt screen

A screen of salt forms as alternatives to the hydrochloride was carried out at Drugabilis. Ten pharmaceutically acceptable counter ions were assessed and three were selected for further study

(citrate, fumarate and phosphate) (Figure **9**). These alternative salt forms of compound **2** were prepared and formulated in the best vehicle determined by injection site tolerability analysis (0.3M sodium acetate buffer, Kolliphor EL, N-methyl pyrrolidone, 2-pyrrolidone, and 11% water suitable for injection). Rat IM pharmacokinetics was then carried out with each salt to determine the salt form to use for efficacy. None of these salt forms offered any improvement in rat IM pharmacokinetic profile over the free base in the tolerated vehicle (Figure **S7**).

3. *In vivo* efficacy studies

The *T. congolense* (IL3000) and *T. vivax* (ILRAD V34) cell lines[7, 8], both originating from Kenya, were used in the following *in vivo* mouse studies. For studies in cattle, either *Trypanosoma congolense (savannah)* KONT 2/133, 224) or *T. vivax* STIB 719 was used.

3.1 Mouse efficacy studies for *T. brucei brucei.*

Mouse efficacy studies for *T. brucei brucei* were carried out as previously detailed.[9]

3.2 NMRI mouse efficacy studies for *T. congolense* and *T. vivax.*

Mouse models of infections were carried out using standardized protocols. Briefly, 1 mL samples of cryopreserved *T. congolense,* or *T. vivax*-infected blood harvested directly from an infected mouse, were diluted with Hanks Balanced Salt Solution plus 20 mM glucose (HBSS+G) to 5 x 10^4 trypomastigotes/mL (5 x 10^5 for *T. vivax*). Female mice (NMRI) were inoculated IP on day 0 with 0.2 mL/animal (equivalent to ~ 1 x 10^4 *T. congolense* or ~ 1 x 10^5 *T. vivax* per animal). The level of parasitaemia was inspected microscopically on day 3 using tail blood smears, counting 20 high powered fields (× 400 magnification). For untreated or treated animals whose tail smears exceeded 50 parasites/field, precise parasite levels were established using a Neubauer haemocytometer; mice with a parasite burden over 1 x 10^8 were promptly and humanely terminated (*T. congolense* only, from our experience the mice will not survive for a further 24 h). Alternatively, in the case of *T. vivax*-infected mice the immune system will bring the infection to a sub-lethal level (below limit of detection)

followed by cycles of high and low parasitaemia. Eventually mice infected with *T. vivax* succumb to the infection between 30 – 60 days post-infection.

Suppression of parasitaemia - mouse

On day 3 post-infection, animals were randomly allocated to groups, with 3 mice/group. Infected animals were dosed with one of the following: diaminazene diaceturate IP. – 40 mg/kg once only as a control (early-stage trypanocide used to clear the parasites in the systemic circulation and in tissues other than CNS), compound **1** (SC) at 3, 10, 30 and 50 mg free base/kg twice and once daily for 4 d or a single dose, or compound **2** (SC) at 3 and 10 mg free base /kg once daily for 4 d, once daily for 2 d or a single dose, one group of 3 mice acted as an untreated control group. The dose solutions were prepared daily, using 5% DMSO: 40% PEG400: 55% MilliQ H_2O for test compounds and 10% DMSO: 90% peanut oil for standard drugs. Alternatively, compound **2** was delivered for 48 h via SC implanted ALZET osmotic pumps (6, 12, 25 and 50 mg free base/mL, vehicle: 50% DMSO and 50% deionized water). Mean blood concentration data presented in Figure S8.

Response to treatment was determined by monitoring parasitaemia twice a week until day 21. After day 21, the mice were checked once a week until 63 d post-treatment. Mice surviving to the end of the experiment, and with blood smears clear of parasites, were considered cured.

3.3 Cattle efficacy for compound 1 (Clinvet)

In each of the cattle efficacy studies (3.3, 3.4 and 3.5), efficacy was assessed primarily on absence of recrudescence of parasitaemia for 100 days after treatment administration together with improvements in Packed Cell Volume, body weight and clinical signs of infected compared with uninfected animals. Assessment of parasitaemia was based on phase contrast buffy coat examinations and confirmation by PCR at 100 d.

Friesian-Holstein calves, weaned for at least 2 months and with a minimum age of 4 months, housed in a fly-proof building, were infected with *Trypanosoma conglense (savannah)* KONT 2/133, at a dose of 110,000 parasites (study day 0) via IV infusion. The parasites were obtained from a donor calf. This isolate is resistant to diminazene (20 mg/kg) and isometamidium (1 mg/kg) in cattle, and diminazene (7 mg/kg) and isometamidium (>1 mg/kg) in mice. Calves were parasitaemic by study day 6. Calves (n = 5) were treated with compound **1** (5 mg/kg, SC, twice 12 h apart) on study

day at 25 mg/ml in NMP/ 2-pyrrolidinone/ PEG400 water 10/40/30/20. Control groups were comprised of un-infected calves, infected but un-treated calves (n = 6) and calves treated with diminazene (n = 3) or isometamidium (n = 3). The calves were randomised to treatments. Calves were monitored daily for parasitaemia and packed cell volume (PCV) via blood sampling and through clinical symptoms.

One day after treatment, 80% of the animals in the treatment group were negative for parasites. After 3 d, 100% of the animals in the treatment groups were negative for parasites. After 5 d, 40% of the animals were positive for parasites. After 6 d, 80% were positive and after 8 days, 100% were positive.

3.4 Cattle efficacy for compound 2, intravenous infusion (Clinvet)

Suppression of parasitaemia - cattle

Friesian-Holstein calves (similar age and housing to those in 3.3) were infected on day 0. Animals were infected with fresh cattle blood from animals infected with *T. congolense* KON 2/133 (n = 4) or *T. vivax* STIB 719 (n = 4). Compound **2** was administered by IV bolus followed by a 24 h IV infusion, followed by another IV bolus and then an additional 12 h intravenous infusion. The concentration of the infusion was 2 mg of free base/ ml). The total dose was 14.8 mg/kg. Compound **2** was administered in 5% (w/v) glucose. Control cattle (n = 3 for *T. congolense* and n = 3 for *T.* vivax) were dosed with saline. Blood samples were collected for PK analysis from treated animals before administration of the compound and at the following times post-administration of the first bolus (+1, +2, +4, +8, +10, +12, +16, +20, +24 (before second bolus), +25, +26, +28, +32, +34, +36, +40, +48 h). Cattle were monitored for packed cell volume (PCV) daily from days 1 to 14 and then 3 times weekly. They were monitored for parasitaemia 3 times per week, from day 1 to day 114.

3.5 Cattle efficacy for compound 2, intramuscular (CIRDES)

Felani-zebu cattle were housed in a fly-proof facility, acclimatised for 15 d prior to the study, cattle were treated with an anthelmintic, an ectoparasiticide and an antibiotic. Donor animals were infected with stabilates (frozen stocks) of either *T. congolense* KOT256 (resistant to 7 mg/kg diminazene in cattle) or *T. vivax* Gando Bongaly (resistant to diminazene 7 mg/kg and to isometamidium 1 mg/kg

in cattle). Blood from the donor animals, diluted with phosphate buffered glucose was used to infect recipient animals IV via the jugular vein. Infection was with approximately 100,000 fresh viable parasites. The compounds were given IM. Each injection was up to 10 mL and given in neck in the following order: left anterior third, right anterior third, left posterior third and right posterior third. Animals over 240 kg required a fifth injection site for the IVP, and this was given in the left or right hind leg. Cattle were randomised to treatments. Parasitaemia was monitored 3 times per calendar week, though collection of blood and assessment of clinical symptoms. Blood samples were also taken for PK analysis (before treatment, 1, 2, 4, 8, 12, 16, 20, 24, 32, 36, 48, 56 and 60 h post treatment). Cattle were also monitored for PCV, clinical symptoms, body weight and temperature (Tables **S5** and **S6**).

4. Mode of action studies

4.1 Resistant line generation

Compound-resistant cell lines were generated by sub-culturing a clone of *T. brucei* in the continuous presence of **2**. Starting at sub-lethal concentrations, drug concentrations in 3 independent cultures were increased in a step-wise manner, usually by 2-fold. When parasites were able to survive and grow in concentrations of drug equivalent to more than 10 times the established EC_{50} value, the resulting cell lines were cloned by limiting dilution in the presence of compound. Three clones (RES I-III) were selected for further biological study.

4.2 Whole genome sequencing and analysis

A standard alkaline lysis protocol was used to isolate genomic DNA from compound-resistant bloodstream *T. brucei* parasites (~5 x 10^8). Whole genome sequencing was performed using a HiSeq4000 next generation sequencing platform (Beijing Genomics Institute, Hong Kong). Sequencing reads (100 bp) were aligned to the *Trypanosoma brucei* TREU927 genome (v39; tritrypDB) using Bowtie2[10] and Samtools[11] software. Samtools and BCFtools[12] were used to call single nucleotide polymorphisms (SNP) and indels compared with the wildtype starter clone, where

overall quality score (QUAL) was >100. Artemis[13] was used to analyse chromosome and gene copy number variation (CNV), as well as visualisation of SNPs.

4.3 RIT-seq screening, Illumina sequencing and analysis

The RIT-seq library screening was performed as described previously.[14] The RNAi library was maintained with blasticidin (1 µg ml^{-1}) and phleomycin (1 µg ml^{-1}) at a minimum of 2 x 10^7 cells. Following tetracycline (1 µg mL^{-1}) induction for 24 h, compound **2** (4 nM), and compound **12** (98 nM), were added to cultures and supplemented with fresh compound and tetracycline as required. DNA was extracted from compound-selected cells and RNAi target fragments were amplified from compound-selected parasites by PCR using the Lib2f and Lib2r primers.[14] PCR products were fragmented and sequenced with an Illumina HiSeq platform at BGI (Beijing Genomics Institute). Reads were mapped to the *T. brucei* 927 reference genome (v39; TriTrypDB [http://tritrypdb.org/]) using Bowtie 2 software,[10] with the following parameter: --very-sensitive-local. Following manipulation with SAMtools[11] the alignment files were searched with a custom script to identify reads with the following barcode: GCCTCGCGA.[14] The total and barcoded reads were then quantified using the Artemis genome browser.[15]

T. brucei CRK12 (Tb927.11.12310) editing

For *Tb*CRK12 mutagenesis at positions L^{482} or G^{492}, we used a bloodstream-form *T. brucei* Cas9 inducible expression system.[16] The gRNA construct was assembled by annealing Fwgcrk12_b (AGGGAATGTGCTCATTAATAGGGA) and Rvgcrk12_b (AAACTCCCTATTAATGAGCACATT) and the resulting gRNA fragment was ligating to the BbsI-digested pT7^{sgRNA} construct. Correct integration was confirmed by sequencing selected clones, which were then transfected into 2T1$^{T7\text{-}Cas9}$ cells. The repair templates were CRK12_482temp: (CCGTGATGTCAAGGCTGACAATGTG**NNN**ATCAACAGAGACGGCAGCGTCCAGCTTGGGGACTTCG) and CRK12_492temp: (ATGTCAAGGCTGACAATGTGCTCATCAACAGAGACGGAAGTGTACAACTC**NNN**GACTTCGGTTTGTGCGCCTTTGAGG)

with randomised codons at the appropriate positions shown in bold; synonymous edits that should promote insertion of randomised codons are underlined. Each template (40 μg) was delivered to the sgRNA strain by electroporation 24 h after Cas9-induction, using tetracycline at 1 μg/mL; tetracycline was removed at this point. Compound **2** selection (10 nM) was applied 6 h later. After 5 days of selection, drug-resistant cultures were subcloned and six clones were selected from each experiment. DNA was extracted from each subclone and a specific portion of the *Tb*CRK12 gene encompassing the edited region was PCR-amplified and sequenced.

4.4 SILAC pulldown

Trypanosoma brucei procyclic form parasites were grown in SDM-79 media (SILAC SDM-79, 2 g/L $NaHCO_3$, 7.5 mg haemin, 1% glutamax and 15% dialysed FBS) with either light or heavy isotopes for 6-9 divisions (6 days) to a density of ~3 x 10^7 cells/ml. A total of 3 x10^8 cells were washed thrice with PBS. Buffer 1 (0.1 mM tosyl phenylalanyl chloromethyl ketone, 1 μg/ml leupeptin, 1 mM aprotinin, 1 mM phenylmethylsulfonyl fluoride, 1x Roche Complete protease inhibitors) was added to the parasite pellet, followed by Buffer 2 (100 mM Tris pH 7.5, 10% glycerol, 300 mM NaCl, 3 mM $MgCl_2$, 1.6% IGEPAL® CA-630, 2 mM dithiothreitol) and incubated at 4°C for 20 min. Lysates were centrifuged at 100,000 g at 4°C for 1 h. Beads (blank and compound-bound) were washed twice with water and twice with lysis buffer (Buffer 1 and 2). Lysates were incubated with 2 mg blank beads for 30 min at 4°C. Lysates were then incubated with either 10 μM DDD00770675 or 1% DMSO for 30 min at 4°C. Finally, lysates were incubated with 2 mg of bound compound for 1 h at 4°C. Beads were washed three times with wash buffer (0.5% IGEPAL® CA-630, 50 mM Tris pH 8.0, 5 mM EDTA, 1 mg/ml BSA) and twice with Tris-buffered saline. Samples were combined, run 1.5 cm into a Brs-TRIS 10% acrylamide gel and stained with Coomassie quick reagent for 1 h. Gel bands were subjected to in-gel reduction with 10 mM dithiothreitol, alkylation with 50 mM iodoacetamide and digestion with 12.5 μg/mL trypsin (Pierce) for >16h at 37°C. Recovered tryptic peptides were vacuum dried.

4.5 Peptide fractionation and LC-MS/MS analysis

Peptides were fractionated using High pH RP Chromatography. A C18 Column from Waters (XBridge peptide BEH, 130Å, 3.5 µm 2.1 x 150 mm, Waters, Ireland) with a guard column (XBridge, C18, 3.5 µm, 2.1 ×10 mm, Waters) were used on a Ultimate 3000 HPLC (Thermo-Scientific). Buffers A and B used for fractionation consist, respectively, of (A) 10mM ammonium formate in milliQ water pH 9.5 and (B) 10 mM ammonium formate, pH 9.5 in 90% acetonitrile. Fractions were collected using a WPS-3000FC auto-sampler (Thermo-Scientific) at 1 min intervals. Column and guard column were equilibrated with 2% Buffer B for twenty minutes at a constant flow rate of 0.2 mL/min. Peptides (190 µL) were injected onto the column, and the separation gradient was started 1 min after sample was loaded onto the column. Peptides were eluted from the column with a gradient of 2% Buffer B to 60% Buffer B in in 53 min. The column was washed for 15 min in 100% Buffer B and equilibrated at 2% Buffer B for 20 min. The fraction collection started 1 min after injection and stopped after 70 min (total 70 fractions, 200 µl each). The total number of fractions concatenated was set to 10 and the content of the fractions was dried and suspended in 25 µL of 1% formic acid prior to analysis with LC-MS. Analysis of peptide readout was performed on a Q Exactive™ plus, Mass Spectrometer (Thermo Scientific) coupled with a Dionex Ultimate 3000 RS (Thermo Scientific). LC buffers used are the following: Buffer A (0.1% formic acid in Milli-Q water (v/v)) and Buffer B (83.7% acetonitrile and 0.1% formic acid in Milli-Q water (v/v). Aliquots of 15 µl per fraction were loaded at 10 µL min^{-1} onto a trap column (100 µm × 2 cm, PepMap nanoViper C18 column, 5 µm, 100 Å, Thermo Scientific) which was equilibrated with 98% Buffer A. The trap column was washed for 5 min at the same flow rate and then the trap column was switched in-line with a Thermo Scientific, resolving C18 column (75 µm × 50 cm, PepMap RSLC C18 column, 2 µm, 100 Å). The peptides were eluted from the column at a constant flow rate of 300 nL/min with a linear gradient from 2% buffer to 35% Buffer B in 125 min, and then to 98% Buffer B by 127 min. The column was then washed with 98% Buffer B for 20 min and re-equilibrated in 2% Buffer B for 17 min. Q-Exactive plus was used in data dependent mode. A scan cycle comprised MS1 scan (m/z range from 335-1600, with a maximum ion injection time of 20 ms, a resolution of 70,000 and automatic gain control (AGC) value of 1×106) followed by 15 sequential dependent MS2 scans (with an isolation window set to 1.4 Da, resolution at 17500, maximum ion injection time at 100 ms and AGC 2×105. To ensure mass accuracy, the mass

spectrometer was calibrated on the first day that the runs were performed. LC-MS analysis was performed by the FingerPrints Proteomics Facility (University of Dundee).

4.6 Data analysis

MS data analysis was performed using the software MaxQuant (http://maxquant.org/, version 1.6.2.6a). Carbamidomethyl (C), oxidation (M), acetyl (Protein N-term), deamidation (NQ) and Gln-> pyro-Glu were set as a variable modification. Proteins were identified by searching a protein sequence database containing *T. brucei brucei* TREU927 annotated proteins (downloaded from TriTrypDB 42, http://www.tritrypdb.org). Trypsin/P was selected as the digestive enzyme with two potential missed cleavages. The FDR threshold for peptides and proteins was 0.01. FTMS MS/MS mass tolerance was set to 10 ppm and ITMS MS/MS mass tolerance was 0.6 Da. Normalized SILAC ratios were calculated using unique and razor peptides and required a minimum of two SILAC pairs. Data was visualized using Perseus 1.6.2.1 (https://maxquant.org/perseus/).

5. Molecular modelling

The sequence of *Tb*CRK12 kinase domain (residues 323-685 of the Tb927.11.12310 entry in the TriTrypDB database) was used to identify homologues performing a Blast search on the Protein Data Bank proteins. The structure of the *human* CRK13 structure complexed with ADP (PDB ID 5EFQ), displaying a sequence identity of 35%, was selected as template for homology modelling. The sequence alignment between the Tb (target) and human (template) sequences was generate using the multisequence viewer tool in the Schrödinger modelling platform (Schrödinger 301 Release 2019-3: Schrödinger, LLC, New York, NY, 2020) and was manually curated to ensure that the highly conserved kinase catalytic residues were correctly aligned (Figure **S10**). The homology model of the *Tb*CRK12:ADP structure was generated using the energy-based method implemented in the Schrödinger protein structure prediction module Prime. Low energy conformers for compound **2** were generated using LigPrep (Schrödinger 301 Release 2019-3: Schrödinger, LLC, New York, NY, 2020). The prepared ligand was then docked into the ATP binding of the *Tb*CRK12 model using Glide (Schrödinger 301 Release 2019-3: Schrödinger, LLC, New York, NY, 2020) in standard

precision mode. No distance or hydrogen-bond constraints were applied. The 10 best scoring docking poses obtained using the default settings were further optimised by post-docking molecular energy minimisation. A 20 Å cubic box centred on the centroid of the ADP molecule was used to generate the docking grid. Within the grid both hydroxyl and thiol groups were allowed to rotate.

6. Supplementary figures

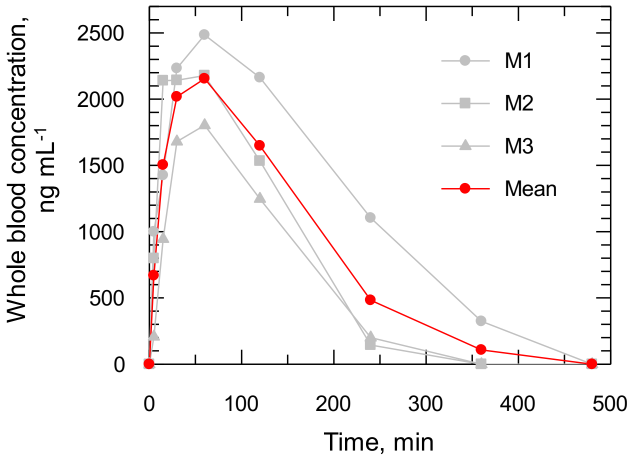

Figure S1: The pharmacokinetics of compound **1** following single SC administration to the female NMRI mouse at 10 mg free base/kg (n=3).

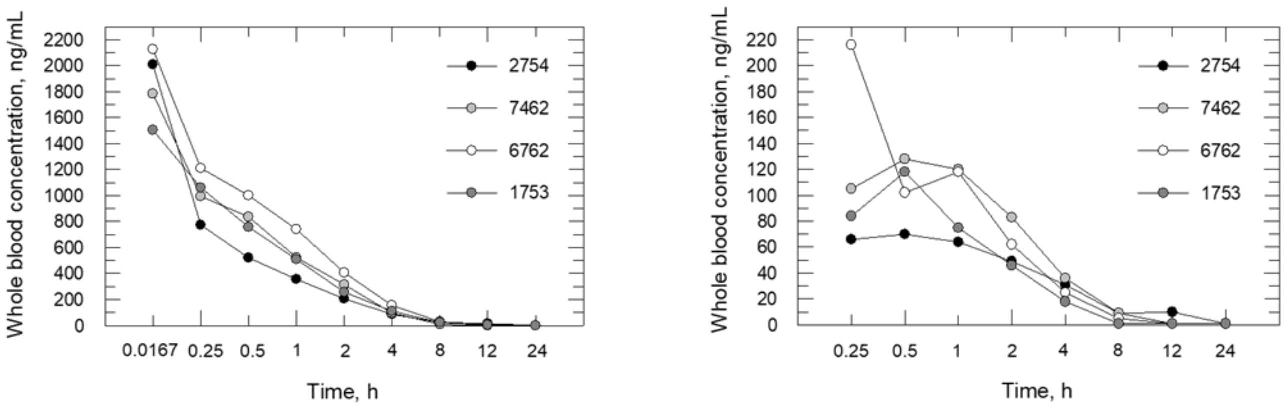

Figure S2: The pharmacokinetics of compound **1** over 24 h following IV (left panel) and IM (right panel) administration to male Fresian-Holstein cattle (n=4) at 2.5 mg free base/ kg. For IV bolus injection, the vehicle was 2.5% NMP, 30% PEG400, 37.5% Propylene Glycol, 30% deionised water. For IM injection, the dose vehicle was 10% *N*-Methyl-2-Pyrrolidinone (NMP), 40% 2-pyrrolidinone, 30% PEG400, 20% deionized water

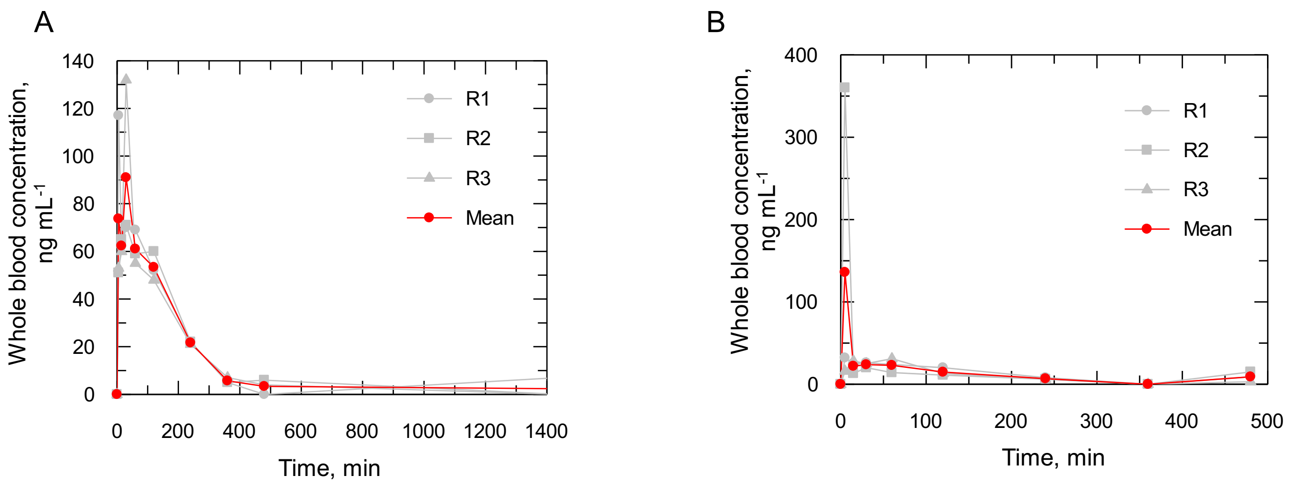

A: 83.4% PVP/2-pyrrolidone; 16.6% saline

B: 80% vehicle 1 (50% N-methyl-2-pydrrolidone: 50% 2-pyrrolidone): 20% vehicle 2 (5% 2-propanol: 95% 0.86% sodium chloride solution).

Figure S3: The pharmacokinetics of compound **1** following single IM administration to the female Sprague Dawley rat at 5 mg free base/kg using two different formulations (n=3 per formulation). Free concentration in blood <EC_{99} at 3 or 4 h, respectively.

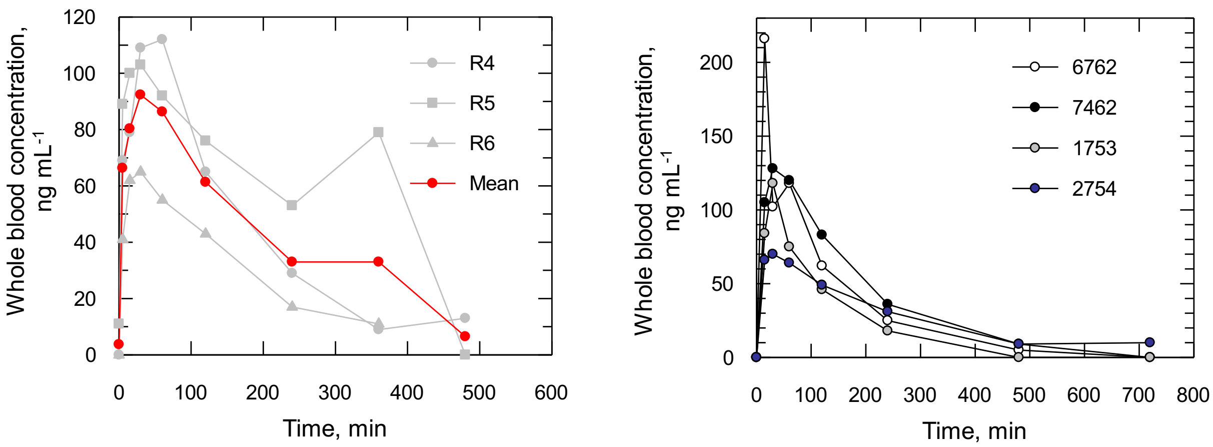

Figure S4: Comparison of the pharmacokinetics of compound **1** following single IM administration to female Sprague Dawley rats or to male Fresian-Holstein cattle following IM administration. Both experiments were conducted using the same formulation (10% NMP, 30% 2-pyrrolidinone, 40% PEG400, 20% MilliQ H_2O) at the same dose and dose level (5 mg/kg; 25 mg/mL; 0.2 mL/kg). Comparable mean drug exposure was observed (Rat: Cmax 92ng/mL, AUC_{0-8} 21265ng-min/mL; Cattle: Cmax 133ng/mL, AUC_{0-8} 20924ng-min/mL).

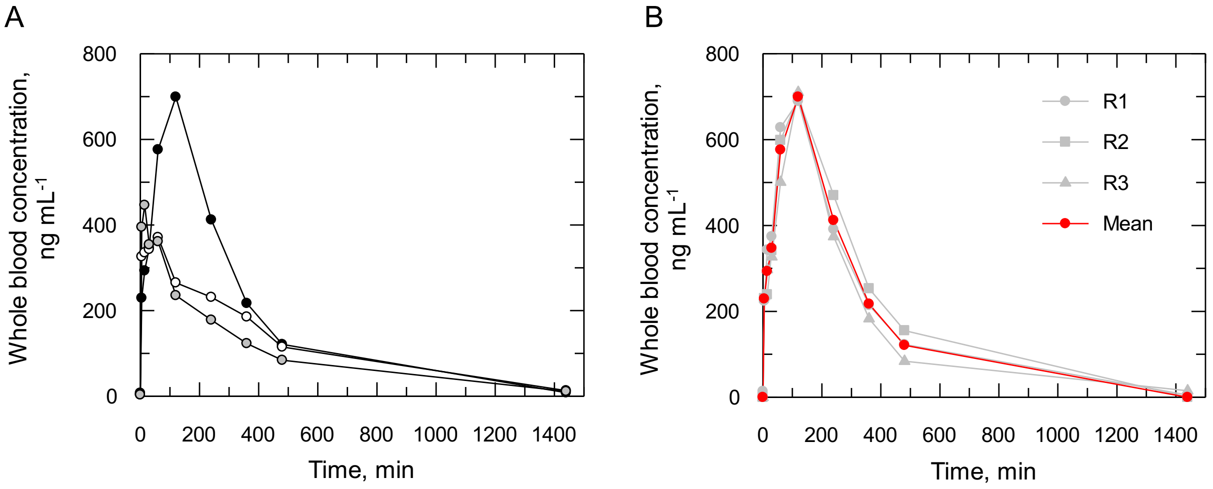

Figure S5: (A) The pharmacokinetics of compound **2** following single IM administration to female Sprague Dawley rats at 10 mg free base/kg using three different formulations (n=3 rats per formulation). Shown in black: 40% 0.3 M Acetate Buffer/ 20% Kolliphor EL/ 20% Glycerol Formal/ 20% 2-pyrrolidone. Shown in white: 50% 0.3 M Acetate Buffer/ 25% Glycerol Formal/ 25% 2-pyrrolidone. Shown in grey: 83.4% 2-pyrrolidinone/polyvinylpyrrolidone/ 16.6% saline. (B) The blood concentration time profile of compound **2** in female NMRI mice following single SC administration at 10 mg free base/kg using used 5% DMSO/ 40% PEG400/ 55% MilliQ water. $AUC_{0\text{-}24}$ 129608 ng-min/mL; Cmax 818 ng/mL; Cav 78 ng/mL; Cmin (8 h) 10 ng/mL.

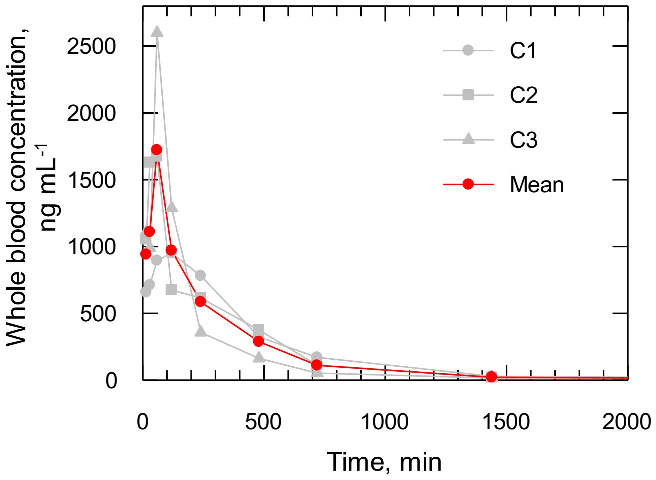

Figure S6: Blood concentration time profile of compound **2** in Friesian cattle following single IM administrations at 10 mg free base/kg. AUC 439785 ng-min/mL; Cmax 1723 ng/mL; Cav 253 ng/mL; Cmin (24 h) 20 ng/mL.

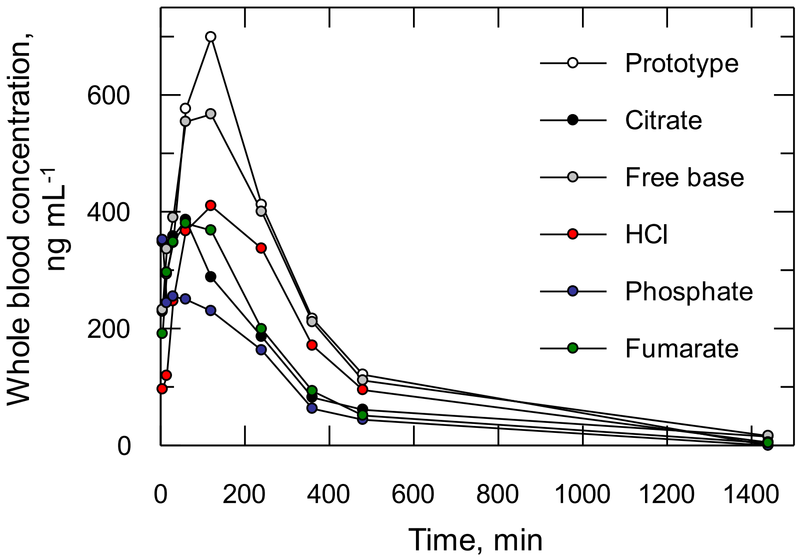

	Formulation					
	Prototype (10 mg kg^{-1})	Free base (10 mg kg^{-1})	HCL salt (10 mg kg^{-1})	Citrate salt (6.632 mg kg^{-1})	Fumarate salt (6.594 mg kg^{-1})	Phosphate salt (8.186 mg kg^{-1})
**AUC (ng-min/mL)**	250,000	230,000	180,000	130,000	130,000	97,000
**Dose normalised AUC (ng-min/mL)**	25,000	23,000	18,000	20,000	19,000	12,000

Figure S7: Exposures of different salt formulations when dosed to male Sprague Dawley rats IM. Prototype formulation was 0.3 M Acetate Buffer (40%), Kolliphor EL (20%), Glycerol Formal (20%) and 2-Pyrrolidone (20 %). Free base and salt forms were prepared and formulated in the best vehicle from the injection site tolerability work (Kolliphor EL, N-methyl Pyrrolidone, 2-Pyrrolidone, and 11% water suitable for injection)

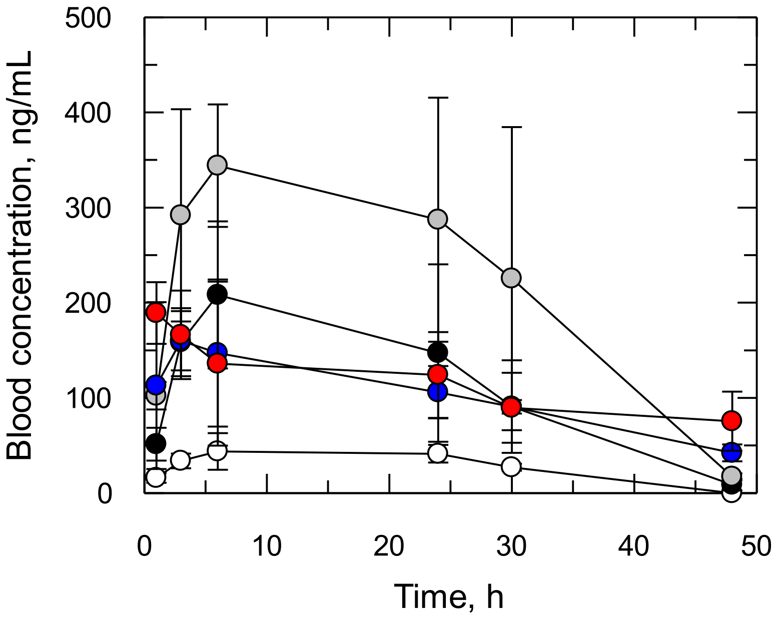

Figure S8: Compound 2 mean (n=3) blood concentration time profile following dosing in T. congolense infected mice via an ALZET pump implant. Using a 2001D ALZET pump (nominal pumping rate of 8ul/hr): 2mg/ml white; 6 mg/mL black; 12 mg/mL grey. Using a 1003D ALZET pump (nominal pumping rate of 1uL/hr): 25 mg/mL blue; 50 mg/mL red. Formulation used was 50:50 DMSO/sterile water. All mice at each dose level were cured.

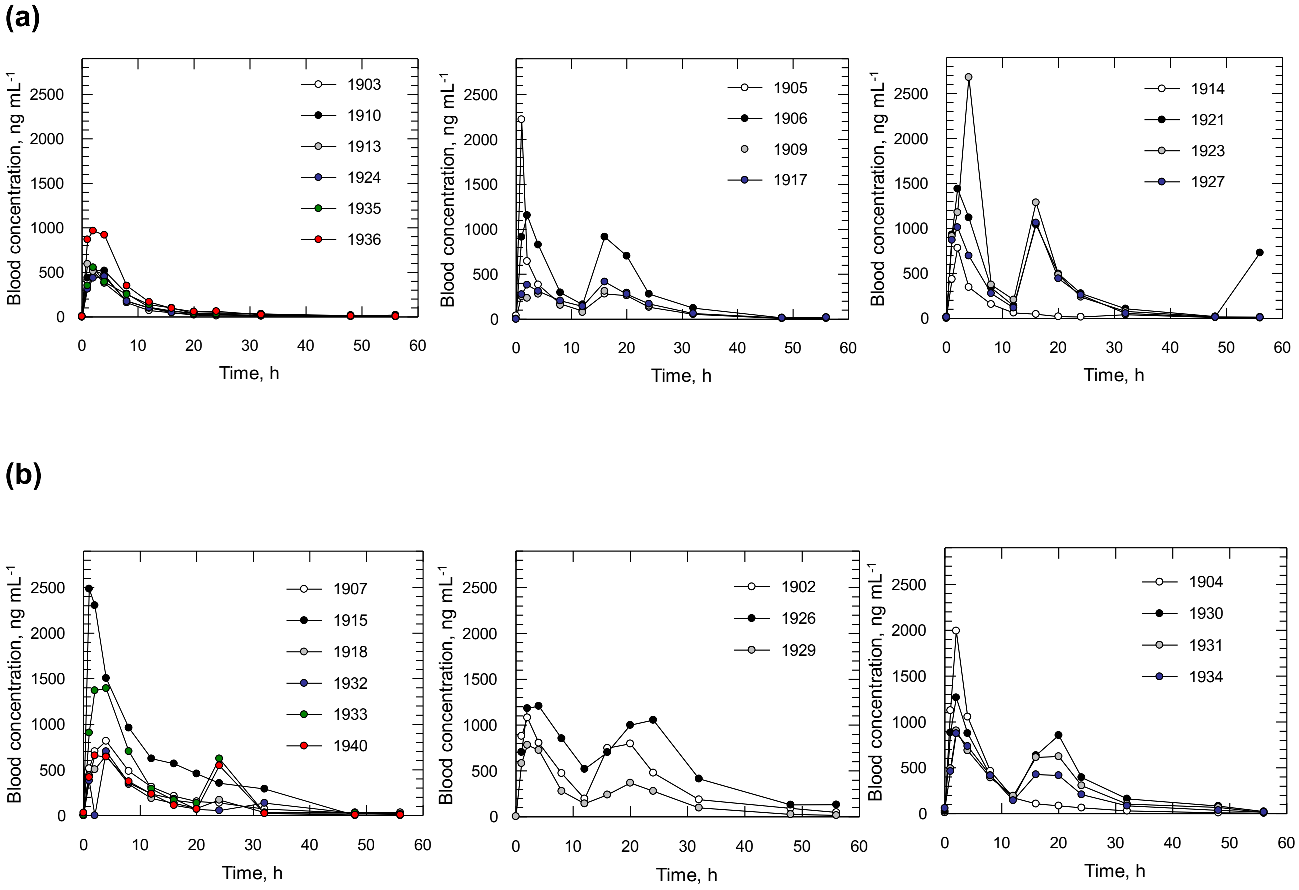

Figure S9: Blood concentration time profiles for compound **2** in (a) *T. congolense*- and (b) *T. vivax*-infected cattle (CIRDES, Burkina Faso). Kolliphor EL, *N*-methyl pyrrolidone, 2-pyrrolidone and 11% water as the formulation using the free base.

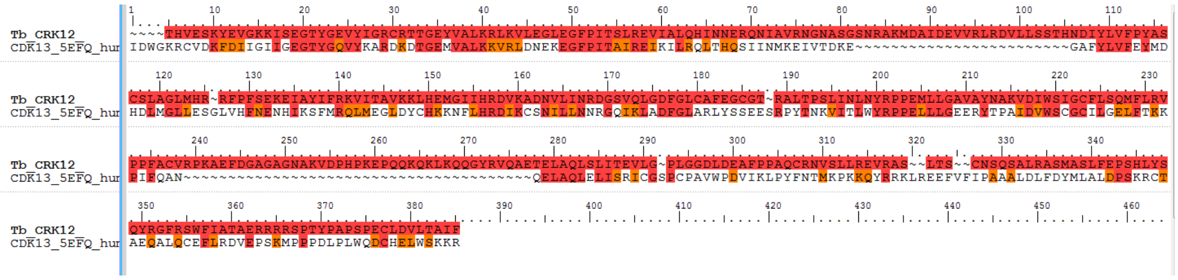

Figure S10. Sequence alignment between the target *Tb*CRK12 (Tb927.11.12310 entry in the TriTrypDB database) and the template *h*CDK13 (from PDB entry 5EFQ) used for the generation of the homology model.

7. LCMS chromatograms for compounds used in in vivo studies

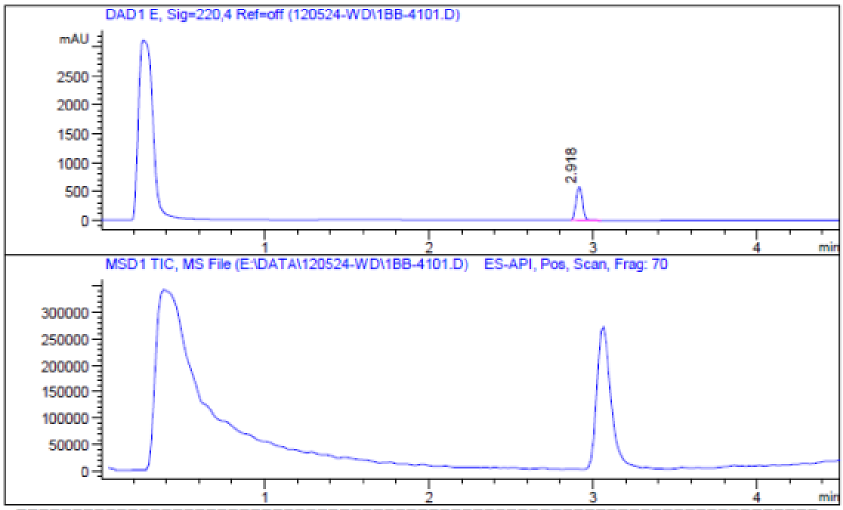

Integration Result

Signal 1 : DAD1 E, Sig=220,4 Ref=off

Peak #	RT [min]	Area	Height	Height %	Width [min]	Area %
1	2.918	1552.253	575.063	100.000	0.043	100.000

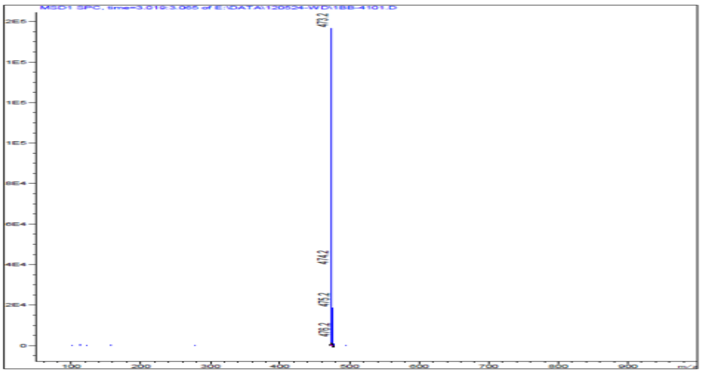

Figure S11. LCMS chromatogram for compound **1**.

Data Express Report — Page 1 of 2

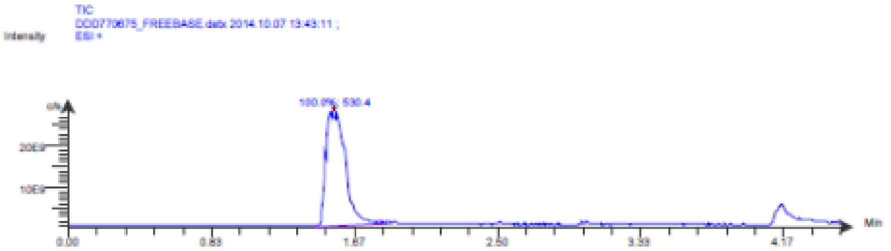

Time (Peak Maximum M:S)	Maximum Intensity	Time (Peak Centroid M:S)	Peak Area	% Peak Area	Peak Resolution	Base Peak Mass
1.54	2.8E10	1.55	2E11	100.0	7.3	530.4

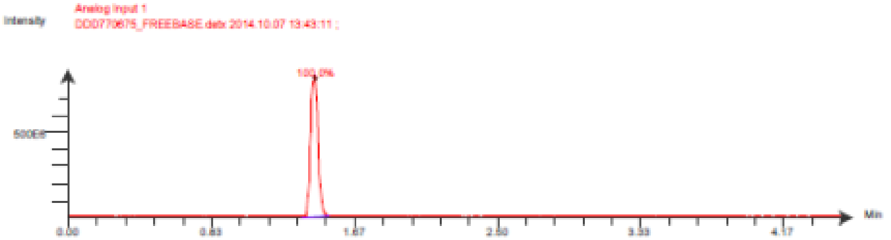

Time (Peak Maximum M:S)	Maximum Intensity	Time (Peak Centroid M:S)	Peak Area	% Peak Area	Peak Resolution
1.43	8E8	1.44	2.3E9	100.0	2.8

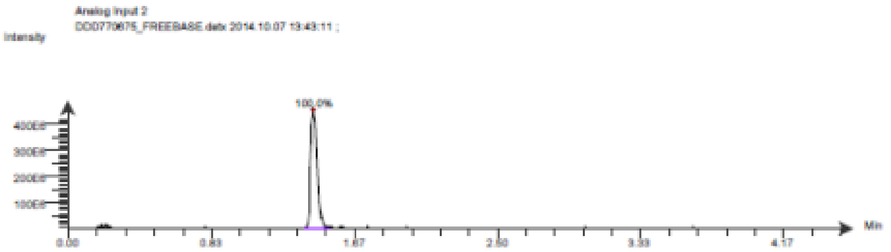

Time (Peak Maximum M:S)	Maximum Intensity	Time (Peak Centroid M:S)	Peak Area	% Peak Area	Peak Resolution
1.43	4.5E8	1.43	1.2E9	100.0	2.5

Data Express Report — Page 2 of 2

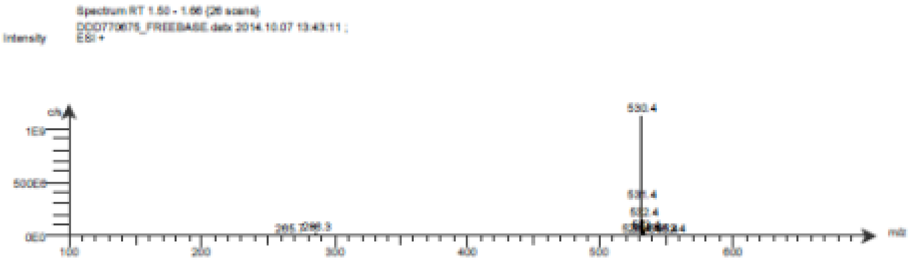

Figure S12. LCMS chromatogram for compound **2**.

Peak#	Time	Area%		Target Mass	BPM	Area	Amount	Start Time	End Time
		DAD1 254	MSD1 TIC+						
1	0.32	4.16				2.66E2		0.21	0.37
2	0.42	7.24				0.46E3		0.37	0.48
3	0.63	5.97				0.38E3		0.48	0.66
4	0.79	17.52				1.12E3		0.66	0.98
5	4.18	65.12	100.00		585.3	0.42E4		4.09	4.62

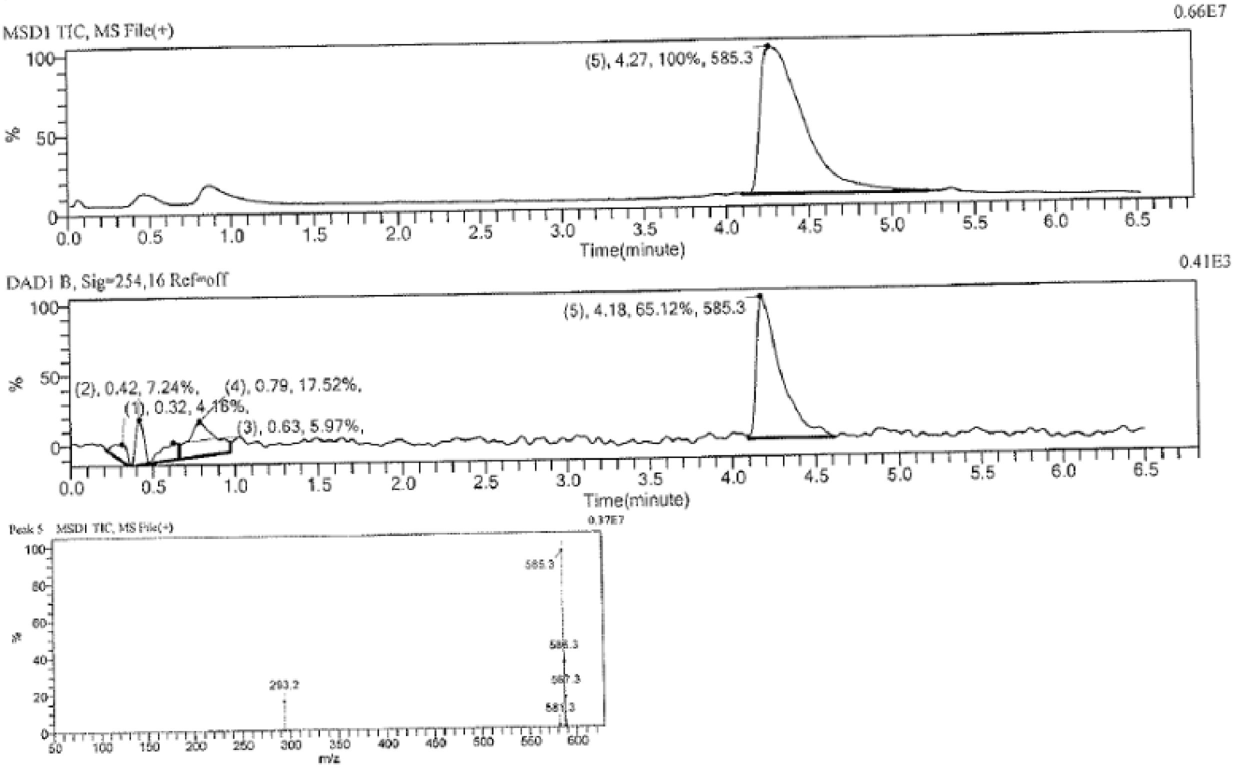

Figure S13. LCMS chromatogram for compound **3**.

Peak#	Time	Area%		Target Mass	BPM	Area	Amount	Start Time	End Time
		DAD1 254	MSD1 TIC+						
1	0.20		4.18		320	1.96E6		0.14	0.35
2	0.40		0.35		267.1	1.66E5		0.36	0.51
3	0.65		0.82		554.2	0.38E6		0.51	0.68
4	0.76	100.00	73.81		572.2	1.44E3		0.64	1.01
5	1.20		0.18		572.2	0.84E5		1.19	1.22
6	1.24		1.56		572.2	0.73E6		1.22	1.61
7	1.67		0.54		215	2.53E5		1.61	1.85
8	1.90		0.28		215	1.33E5		1.85	1.96
9	1.99		0.17		214.9	0.81E5		1.96	2.02
10	2.06		0.27		214.9	1.28E5		2.02	2.10
11	2.44		8.18		572.2	0.38E7		2.10	2.60
12	2.63		1.10		572.3	0.52E6		2.60	2.67
13	2.73		2.12		267.1	0.99E6		2.67	2.78
14	2.84		5.24		399.2	2.45E6		2.78	3.18
15	3.28		1.18		282.3	0.55E6		3.18	3.50

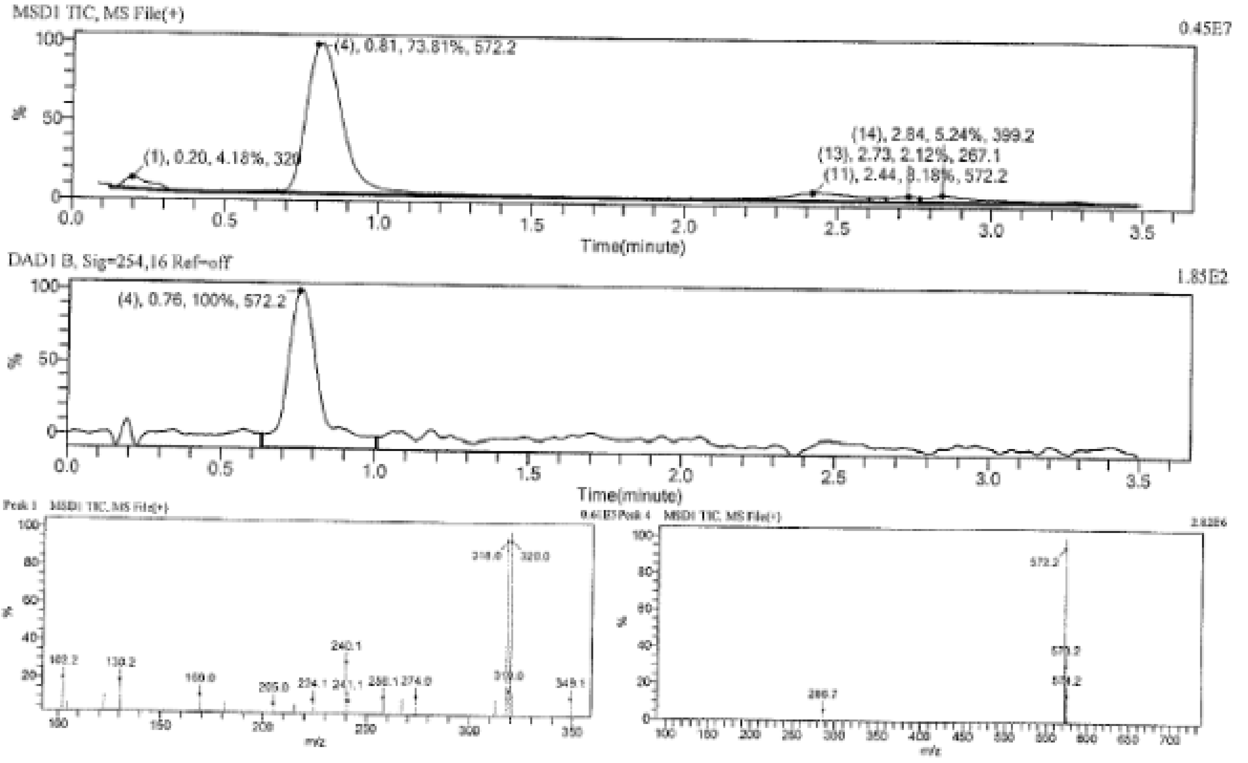

Figure S14. LCMS chromatogram for compound **4**.

Peak#	Time	Area%		Target Mass	BPM	Area	Amount	Start Time	End Time
		DAD1 254	MSD1 TIC+						
1	0.41	14.48				0.45E3		0.36	0.47
2	0.60	34.94				1.08E3		0.47	0.75
3	0.79	14.67				0.45E3		0.75	0.87
4	0.90	10.90				0.34E3		0.87	1.00
5	3.96	25.00	94.81		556.2	0.77E3		3.79	4.10
6	5.15		5.19		399.3	1.82E6		5.08	5.33

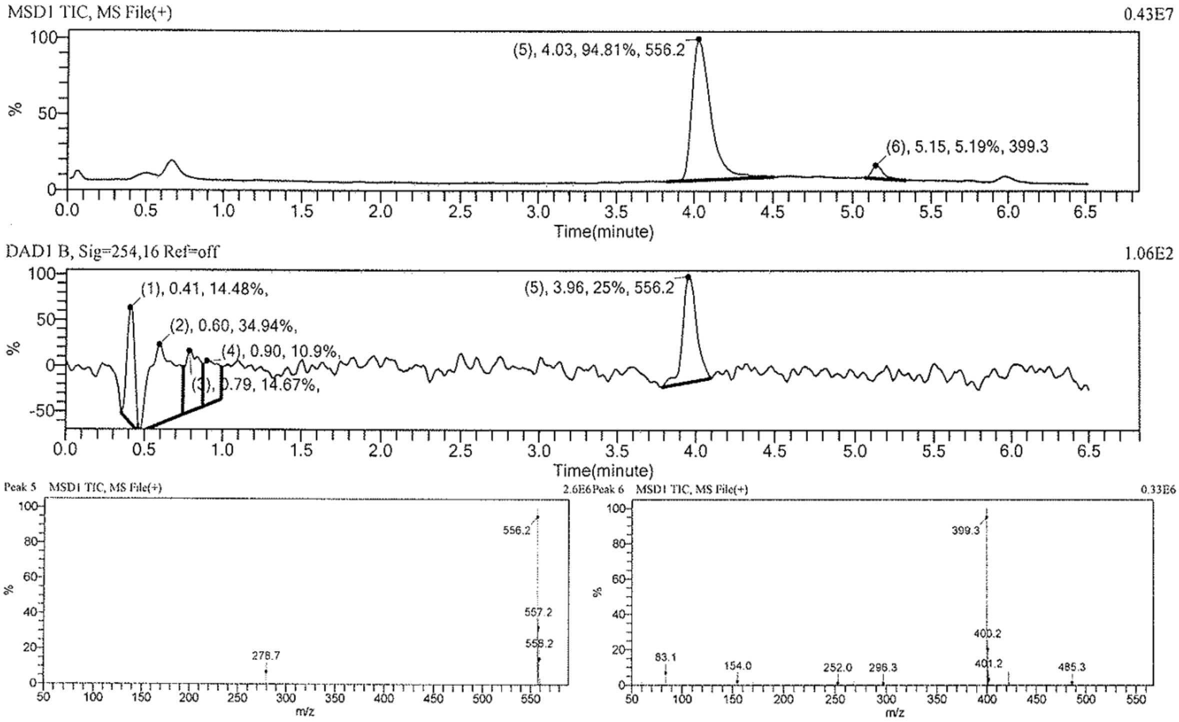

Figure S15. LCMS chromatogram for compound **5**.

Chromatogram

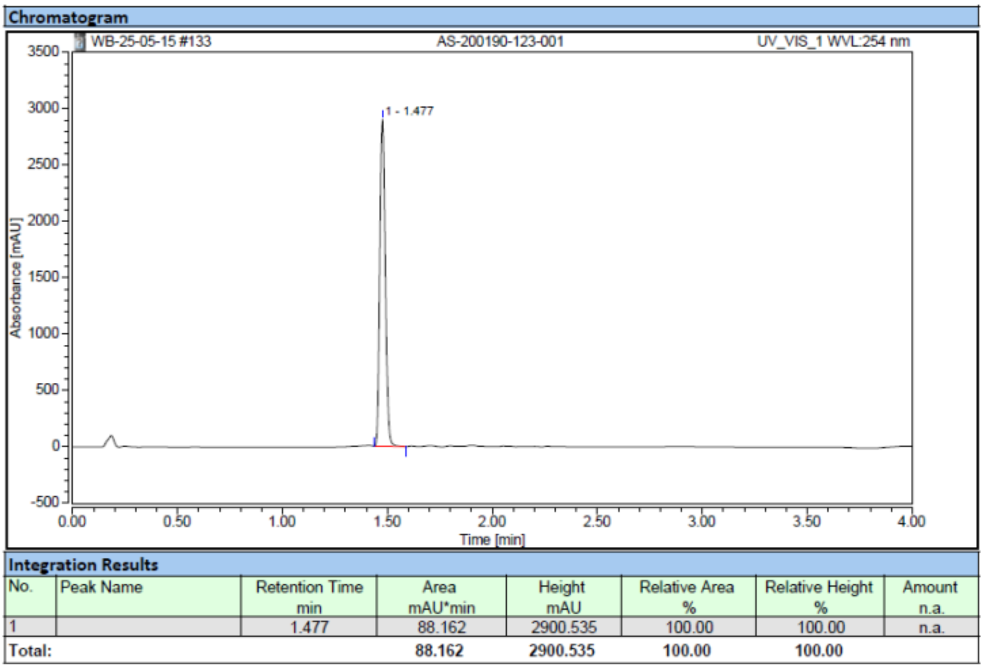

Integration Results

No.	Peak Name	Retention Time<br>min	Area<br>mAU*min	Height<br>mAU	Relative Area<br>%	Relative Height<br>%	Amount<br>n.a.
1		1.477	88.162	2900.535	100.00	100.00	n.a.
**Total:**			**88.162**	**2900.535**	**100.00**	**100.00**	

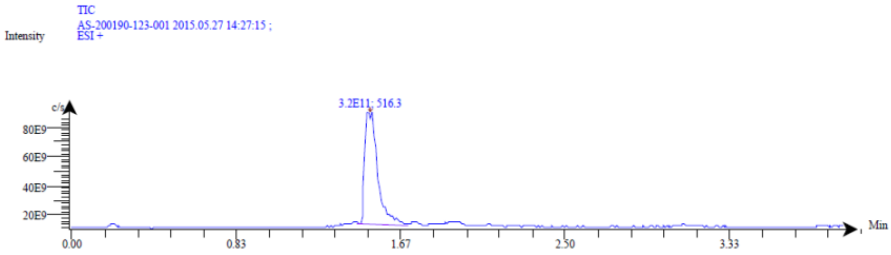

Time (Peak Maximum M:S/Minutes)	Maximum Intensity (c/s)	Time (Peak Centroid M:S/Minutes)	Peak Area	% Peak Area	Peak Resolution	Base Peak Mass (m/z)
1.51	7.7E10	1.51	3.2E11	100.0	3.7	516.3

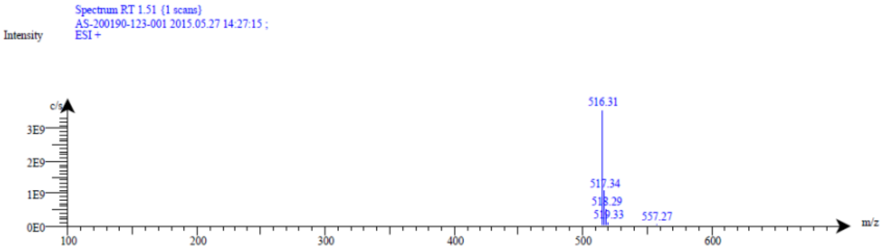

Figure S16. LCMS chromatogram for compound **8**.

Peak#	Time	Area%		Target Mass	BPM	Area	Amount	Start Time	End Time
		DAD1 254	MSD1 TIC+						
1	0.41	14.44				0.48E3		0.36	0.46
2	0.55	10.85				0.36E3		0.47	0.61
3	0.65	8.73				2.91E2		0.61	0.70
4	0.86	17.12				0.57E3		0.70	0.92
5	4.31	48.86	100.00		505.1	1.63E3		4.16	4.42

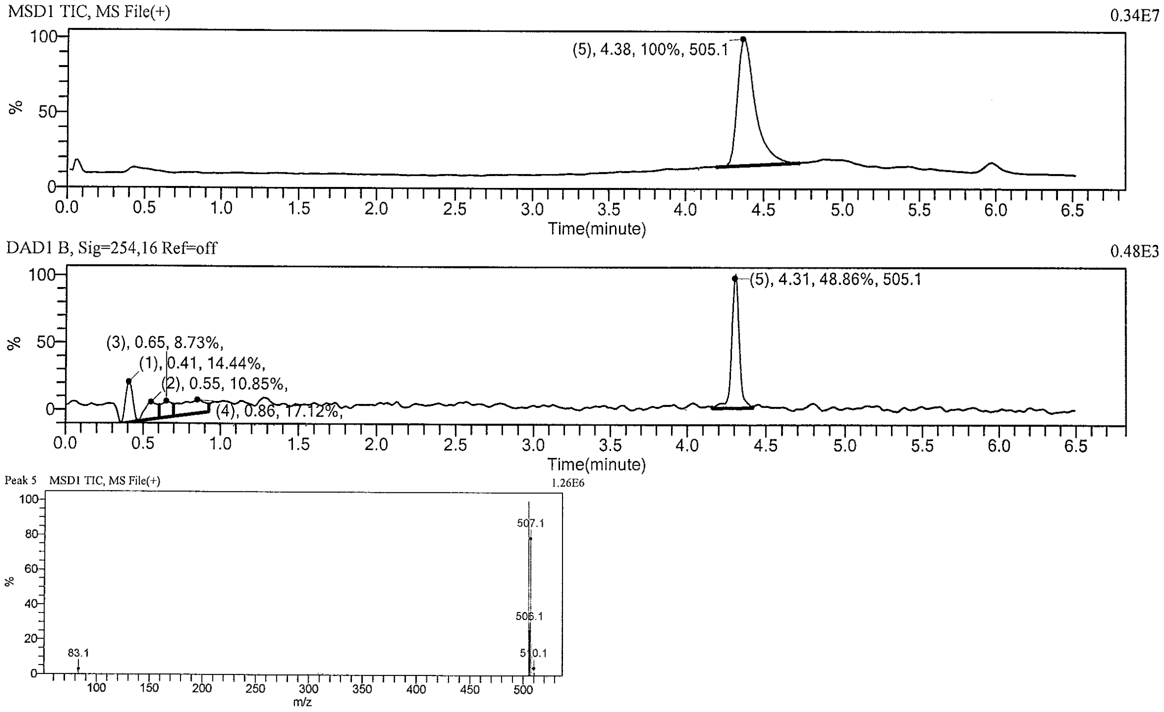

Figure S17. LCMS chromatogram for compound **10**.

8. Supplementary tables

Table S4: Pharmacokinetic parameters of compound **1** following IV (A) or IM (B) administration in male Fresian-Holstein cattle (n=4) at 2.5 or 5 mg free base/kg, respectively.

A: IV

**Parameters**	**Animal no.**			
	**2754**	**7462**	**1753**	**6752**
Clb (mL/min/kg)	24	20	21	15
Vdss (L/kg)	3.5	1.8	2.5	1.7
Half-life (h)	2.4	1.2	1.8	1.7
AUC $_{0-\infty}$ (ng-min/mL)	100,000	120,000	120,000	160,000

B: IM

**Parameters**	**Animal no.**			
	**2754**	**7462**	**1753**	**6752**
Cmax (ng/mL)	70	128	118	216
Tmax (h)	0.5	0.5	0.5	0.25
AUC $_{0-\infty}$ (ng-min/mL)	21,000	26,000	15,000	22,000
F (%)	10	11	7	7

Table S5: Pharmacokinetic parameters of compound **2** following IV (A) or IM (B) administration at 2 or 10 mg free base/kg to the male Friesian-Holstein cattle (n=3), respectively.

A:

**Parameters**	**Animal no.**			**Mean (n=3)**
	**1644**	**3646**	**7643**	
Clb (mL/min/kg)	14	21	11	15
Vdss (L/kg)	2	3	2	3
Half-life (h)	6	6	3	4
AUC $_{0-\infty}$ (ng-min/mL)	140,000	94,000	180,000	130,000

B:

**Parameters**	**Animal no.**			**Mean (n=3)**
	**1644**	**3646**	**7643**	
Cmax (ng/mL)	950	1,700	2,600	1,700
Tmax (h)	2	1	1	1
AUC $_{0-\infty}$ (ng-min/mL)	530,000	510,000	460,000	490,000
F (%)	78	75	66	74

Table S6: Pharmacokinetic data for compound **2** when administered to cattle IM.

IM injection (mL)	Time (min)	Formulation 1<br>HCl salt<br>Kolliphor, Glycerol Formal,<br>2-Pyrollidone,<br>11% Water	Formulation 2<br>HCl salt<br>Kolliphor, NMP,<br>2-Pyrollidone,<br>11% Water	Formulation 3<br>Free Base<br>Kolliphor, NMP,<br>2-Pyrollidone,<br>11% Water
		Scaled concentration (ng/mL)		
		Calf 0242	Calf 0091	Calf 0481
1	pre-dose	≤ 3	≤ 3	≤ 3
	30	12	32	107
	60	13	25	4
	180	42	42	3
	300	20	32	≤ 3
5	pre-dose	≤ 3	≤ 3	≤ 3
	30	62	247	59
	60	68	257	50
	180	50	181	46
	300	55	121	38
10	pre-dose		≤ 3	7
	30		119	93
	60		94	68
	180		90	48
	300		109	29
IM injection (mL)	Time (min)	Scaled concentration (ng/mL)		
		Calf 0110	Calf 0090	Calf 0131
1	pre-dose	≤ 3	≤ 3	4
	30	20	12	8
	60	15	13	6
	180	36	26	8
	300	20	17	11
	pre-dose	≤ 3	≤ 3	≤ 3
	30	54	27	111

5	60	60	47	81
	180	74	74	64
	300	83	84	44
10	pre-dose	-	≤ 3	4
	30	-	29	96
	60	-	67	64
	180	-	107	75
	300	-	157	131

Table S7: Pharmacokinetic Parameters for Compound **2** when dosed to Aberdeen Angus crossbreed and Limousin crossbreed cattle at 5 mg/kg as the free base using Kolliphor EL, N-methyl Pyrrolidone, 2-Pyrrolidone and 11% water as the vehicle. Dosing was IM or SC.

AUC, ng-min/mL		Cmax (obs) ng/mL		Tmax (h)	
IM	SC	IM	SC	IM	SC
190,000	58,000	360	86	0.5	3.0

Table S8: Concentration of compound **2** (ng/mL) in blood following dosing in *T. congolense* infected cattle (CIRDES, Burkina Faso).

	**T1**						**T2**			
	Compound **2** (single dose IM)						Compound **2** (dosed twice IM)			
Animal	1903	1910	1913	1924	1935	1936	1905	1906	1909	1917
Time (h)										
0	<LOQ	<LOQ	10	<LOQ	<LOQ	5	39	4	<LOQ	<LOQ
1	413	439	593	312	351	868	2224	914	251	275
2	525	523	511	436	555	967	644	1156	234	381
4	473	518	379	453	391	920	383	827	279	312
8	159	260	178	174	252	349	169	295	154	204
12	71	108	98	104	138	167	90	162	75	141
16	47	67	64	48	101	96	281	916	312	415
20	22	39	40	28	39	58	261	703	293	278
24	11	30	46	23	34	62	132	279	146	170
32	8	15	33	12	33	24	54	122	53	63
48	18	5	14	5	6	9	7	14	15	12
56	3	21	4	3	4	6	8	20	15	19
Efficacy	no	no	no	no	no	no	no	yes	no	no

	**T3**		**T4**							
	Dosed twice with saline		Dosed twice with saline, followed by two doses of compound **2** rescue IM 5 days later							
Animal	1912	1920	1914	1914	1921	1921	1923	1923	1927	1927
			After saline	After **2**	After saline	After **2**	After saline	After **2**	After saline	After **2**
Time (h)										
0	<LOQ	5	4	15	<LOQ	<LOQ	5	15	5	11
1	4	<LOQ	47	434	16	930	3	912	3	870
2	14	<LOQ	15	781	<LOQ	1440	<LOQ	1176	<LOQ	1011
4	6	<LOQ	<LOQ	344	4	1120	<LOQ	2680	<LOQ	695
8	<LOQ	<LOQ	4	155	<LOQ	339	3	373	<LOQ	275
12	4	<LOQ	5	60	6	137	<LOQ	205	<LOQ	116
16	<LOQ	11	4	45	14	1041	19	1287	7	1062

20	357	6	<LOQ	19	4	494	4	484	<LOQ	441
24	<LOQ	72	3	14	13	276	12	236	<LOQ	258
32	<LOQ	<LOQ	3	39	22	105	51	76	71	54
48	3	5	4	8	104	18	<LOQ	16	28	11
56	<LOQ	4	<LOQ	4	3	728	5	12	<LOQ	8
Efficacy	no	no	no	no	no	yes	no	yes	no	no

LOQ = 2 ng/ml

Table S9: Concentration of compound **2** (ng/mL) in blood following dosing in *T. vivax* infected cattle (CIRDES, Burkina Faso).

	**T5**						**T6**			
	Compound **2** dosed once IM						Compound **2** dosed twice IM			
Animal	1907	1915	1918	1932	1933	1940	1902	1916	1926	1929
Time (h)										
0	<LOQ	ND	ND	<LOQ	5	29	<LOQ		<LOQ	3
1	514	2485	437	379	907	418	880		704	583
2	702	2304	504	NS	1370	656	1081		1183	782
4	815	1505	657	702	1394	643	806		1208	726
8	485	960	342	357	703	373	475		854	280
12	313	623	186	235	291	235	196		520	143
16	211	567	133	180	166	110	748		703	240
20	153	457	77	67	136	69	797		999	368
24	137	353	169	53	623	546	479		1055	279
32	70	291	31	136	26	21	187		414	97
48	25	NS	32	22	26	5	92		130	26
56	20	NS	32	12	9	5	48		131	15
Efficacy	No	ND	No	No	No	No	No		No	No

ND, not determined

	**T7**		**T8**		**T9**					
	Dosed twice with saline		Dosed twice with saline, followed by single rescue dose of compound **2** IM 4 days later		Dosed twice with saline, followed by single rescue dose of compound **2** IM 4 days later					
Animal	1908	1928	1904	1904	1930	1930	1931	1931	1934	1934
			After saline	After **2**	After saline	After **2**	After saline	After **2**	After saline	After **2**
Time (h)										
0	<LOQ	<LOQ	<LOQ	11	3	28	<LOQ	<LOQ	<LOQ	59
1	<LOQ	<LOQ	<LOQ	1125	<LOQ	883	<LOQ	493	3	462
2	5	4	<LOQ	1994	<LOQ	1265	<LOQ	905	<LOQ	875
4	6	7	7	1056	<LOQ	876	<LOQ	688	3	735
8	14	<LOQ	<LOQ	465	<LOQ	417	<LOQ	391	<LOQ	415
12	<LOQ	<LOQ	4	173	<LOQ	167	3	193	<LOQ	144
16	19	7	<LOQ	107	14	635	<LOQ	613	14	427
20	4	<LOQ	<LOQ	85	<LOQ	854	3	624	<LOQ	416
24	38	5	30	63	101	395	120	305	14	208
32	3	201	<LOQ	30	1165	161	8	104	3	86
48	21	54	39	10	6	82	114	68	24	36
56	2	27	31	5	4	22	<LOQ	13	3	17
Efficacy	No	no	No	ND	No	ND	No	No	No	no

LOQ = 2 ng/ml

Table S10: Metabolism of compound **2** to compound **8**.

	T. congolense (animal 1903)			*T. vivax* (animal 1907)		
Time (h)	Compound **8** Peak area	Compound **2** peak area	% cpd **8**/ cpd **2** by peak area	Compound **8** peak area	Compound **2** peak area	% cpd **8**/ cpd **2** by peak area
0	0	0	0		285	0
1	4,000	180,000	2.2	6,900	160,000	4.2
2	6,600	240,000	2.8	11,000	230,000	4.8
4	9,000	210,000	4.3	19,000	260,000	7.2
8	2,000	69,000	2.9	19,000	160,000	12
12	1,500	32,000	4.7	15,000	100,000	15
16	1,000	21,000	5.0	11,000	68,000	17
20	790	9,900	8.0	8,300	49,000	17
24	380	5,100	7.5	6,300	44,000	14
32	270	3,600	7.5	4,100	23,000	18
48	130	8,000	1.6	1,600	8,300	19
56	130	1,600	8.2	660	6,600	10

Table S11: Identification of *T. brucei* proteins that bind specifically to resin-bound 13. Data derived from analysis of chemo-proteomic data from stable isotope labelled *T. brucei* procyclic lysate incubated with either compound **2** or DMSO followed by incubation with compound-resin (compound **13**). Resin-bound proteins were analysed by LS-MS/MS with a high DMSO/compound ratio indicating specific binding to **13**. Data shown for Log_2 DMSO/compound >1.5, 2.8-fold enrichment. Proteins shown were identified by the detection of at least 2 unique peptides and 2 ratio H/L counts. Data is the mean of three experiments.

Gene ID	Log_2 ratio	Protein description	Abbreviation	Unique peptides
Tb927.1014800	4.15	MAP kinase 9, putative	MAPK9	10
Tb927.6.3110	3.57	Cdc-2 related kinase 11	CRK11	7
Tb927.11.12310	3.47	Cdc2-related kinase 12	CRK12	19
Tb927.11.14030	3.16	Cyclin 9	CYC9	6
Tb927.10.16030	2.84	MAP kinase 4	MAPK4	5
Tb927.1.700	1.92	Phosphoglycerate kinase	PGK	4
Tb927.7.4400	1.85	Inositol polyphosphate kinase-like protein	IPPK	7
Tb927.10.5140	1.62	MAP kinase 6	MAPK6	30

Table S12: Summary of read counts and genome coverage following whole genome sequencing.

Cell line	Number of reads	Read length	Percentage mapped	Fold coverage	Loss of SNP heterozygosity	Gain of SNP heterozygosity	Gain of SNP homozygosity
WT	40710212	100	80.89	90	-	-	-
RES1	40380322	100	81.42	90	852	3	0
RES2	41040418	100	81.47	92	26	3	0
RES3	41109768	100	81.11	91	856	2	1

Table S13: Summary of mutations found in compound 2-resistant lines.

Chromosome	Nucleotide position	Gene ID	WT	Mut	Amino acid change	Resistant lines			Gene name
						R1	R2	R3	
Homozygous mutations (coding regions only)									
11	3291310	Tb927.11.12310	G	C	Gly492Ala	-	-	1/1	CRK12
Heterozygous mutations (coding regions only)									
4	1272440	Tb927.4.4590	A	G	Leu474Pro	0/1	0/1	0/1	AGC family Ser/Thr kinase
11	2749965	Tb927.11.10300	G	T	Glu56STOP	0/1	0/0	0/1	Hypothetical
11	3291279	Tb927.11.12310	C	T	Leu482Phe	0/0	0/1	0/0	CRK12
11	3291310	Tb927.11.12310	G	C	Gly492Ala	0/1	0/1	-	CRK12

Table S14: Extended hit list for Compound 12 RIT-seq analysis (>1000 RPKM 'barcoded reads' cut-off)

		All mapped reads		Barcoded reads only		
Gene ID	Gene description	RPKM	Actual	RPKM	Actual	Fragments
Tb927.9.13470	hypothetical protein, conserved	99232	948093	198910	296661	3
Tb927.10.16125	hypothetical protein	17238	7381	52829	3196	0
Tb927.10.11720	CW-type Zinc Finger, putative	18666	178339	52130	78400	1
Tb927.1.4250	hypothetical protein, conserved	21077	203698	36461	54701	3
Tb927.10.16120	inosine-5'-monophosphate dehydrogenase	13916	87335	26099	24976	8
Tb927.11.11530	histone acetyltransferase, putative	11758	87022	18400	19861	3
Tb927.11.14870	NAD+ synthase, putative	10691	38581	9004	5460	1
Tb927.6.1070	hypothetical protein, conserved	3250	34355	7981	13377	4
Tb927.4.990	hypothetical protein, conserved	2787	11967	7709	4677	0
Tb927.4.980	actin, putative	4334	19775	7423	5258	4
Tb927.7.2760	Mitochondrial ribosomal protein L22	3128	10677	7269	4168	0
Tb927.7.1690	Silent information regulator 2 related protein 1	4015	17290	5089	3167	2
Tb927.10.11690	YEATS family, putative	2415	14331	5013	4476	3
Tb927.6.1670	PAB-dependent poly(A)-specific ribonuclease subunit PAN2 (pseudogene)	2168	28804	4612	9566	8
Tb927.5.1980	Galactose oxidase, central domain/Kelch motif containing protein, putative	2337	18582	3533	4353	4
Tb927.7.2770	hypothetical protein, conserved	6669	33370	3341	2366	2
Tb927.10.8060	SET domain containing protein, putative	3643	20413	3262	3047	2
Tb927.3.5190	hypothetical protein, conserved	1399	6142	2906	1967	2
Tb927.6.2570	hypothetical protein, conserved	731	4293	1913	1886	2
Tb927.10.9160	Cyclin 12, L-type	831	6506	1756	2293	1
Tb927.7.5230	lanosterol synthase	795	8826	1726	3046	5
Tb927.4.2700	Hydroxymethylglutaryl-CoA lyase, mitochondrial, putative	771	4074	1602	1293	3
Tb927.11.1070	glycosomal transporter (GAT3), putative	1049	8787	1591	2154	2
Tb927.2.4510	cdc2-related kinase 9	476	4517	1492	2136	1
Tb927.4.4820	amino acid transporter 10, putative	710	4115	1440	1287	2
Tb927.8.2160	p-glycoprotein	1120	21678	1295	3744	5
Tb927.6.3320	ferric reductase transmembrane protein, putative	595	1747	1260	523	0
Tb927.7.4540	hypothetical protein, conserved	945	3608	1241	789	3
Tb927.7.4400	inositol hexakisphosphate kinase	721	6675	1178	1757	6
Tb927.10.2160	hypothetical protein, conserved	360	757	1174	351	3
Tb927.1.670	conserved protein, unknown function	409	3080	1146	1328	2
Tb927.11.16360	hypothetical protein, conserved	350	1508	1130	720	6
Tb927.4.4840	amino acid transporter 7, putative	544	3156	1130	940	2
Tb927.7.950	Polycystin cation channel, putative	385	2333	1120	959	0
Tb927.3.4050	hypothetical protein, conserved	164	333	1083	311	0

Primary 'hit' >1 fragment per gene
Secondary 'hit' ≤ 1 fragment per gene
Adjacent hit

Table S15: Extended hit list for Compound 2 RIT-seq analysis (>1000 RPKM 'barcoded reads' cut-off)

		All mapped reads		Barcoded reads only		
Gene ID	Gene description	RPKM	Actual	RPKM	Actual	Fragments
Tb927.9.13470	hypothetical protein, conserved	30403	305080	94211	163003	7
Tb927.10.6600	hypothetical protein, conserved	40913	183455	73811	60990	1
Tb927.11.14870	NAD+ synthase, putative	36458	138184	34753	23909	2
Tb927.11.11530	histone acetyltransferase, putative	14460	112397	34314	43909	3
Tb927.1.4250	hypothetical protein, conserved	10010	101604	20465	33845	6
Tb927.10.8100	hypothetical protein, conserved	5755	26247	15417	11585	1
Tb927.6.1070	hypothetical protein, conserved	5553	61645	12721	23833	2
Tb927.7.1690	Silent information regulator 2 related protein 1	9439	42687	10795	8292	2
Tb927.6.4860	S-adenosylmethionine synthetase, putative	4762	24351	7938	6686	9
Tb927.6.4910	S-adenosylmethionine synthetase, putative	4368	22335	7178	5886	10
Tb927.6.4920	S-adenosylmethionine synthetase, putative	4096	20944	7171	5872	6
Tb927.6.4840	S-adenosylmethionine synthetase, putative	4144	21190	7003	5746	9
Tb927.6.4900	S-adenosylmethionine synthetase, putative	4354	22263	6949	5692	10
Tb927.3.2780	hypothetical protein, conserved	2097	29714	6669	15695	6
Tb927.7.4540	hypothetical protein, conserved	4941	19805	6521	4819	3
Tb927.6.4870	S-adenosylmethionine synthetase, putative	3970	20300	6507	5338	9
Tb927.6.4890	S-adenosylmethionine synthetase, putative	3883	19854	6340	5137	9
Tb927.6.4880	S-adenosylmethionine synthetase, putative	3593	18375	5546	4834	8
Tb927.6.4850	S-adenosylmethionine synthetase, putative	3294	16842	5015	4093	8
Tb927.5.1450	receptor-type adenylate cyclase GRESAG 4, putative	4106	65942	4913	14545	1
Tb927.9.9400	ceramide phosphorylethanolamine synthase	1855	7723	3933	2683	1
Tb927.7.2770	hypothetical protein, conserved	33474	175902	3164	2672	3
Tb927.10.8060	SET domain containing protein, putative	1156	6805	2499	2257	4
Tb927.10.13710	p90 ATPase, N-terminal/Activator of Hsp90 ATPase homolog 1-like p	981	4183	2256	1584	5
Tb927.7.2760	Mitochondrial ribosomal protein L22	1001	3587	2169	1431	0
Tb927.10.2240	RNA binding protein	830	6281	1922	2546	4
Tb927.2.4340	hypothetical protein, conserved	464	2572	1850	1730	1
Tb927.9.13030	QA-SNARE protein putative	725	2851	1656	1069	1
Tb927.10.4590	mitochondrial inner membrane signal peptidase, putative	390	1041	1257	613	2
Tb927.2.2015	hypothetical protein	386	124	1169	69	2
Tb927.11.2860	hypothetical protein, conserved	1335	13207	1125	1672	1
Tb927.10.11720	CW-type Zinc Finger, putative	413	4145	1103	1873	3
Tb927.10.9400	splicing factor 1	533	1861	1034	591	1

Primary 'hit' >1 fragment per gene
Secondary 'hit' ≤ 1 fragment per gene
Adjacent hit

9. References

1. Greig, N.; Wyllie, S.; Patterson, S.; Fairlamb, A. H., A comparative study of methylglyoxal metabolism in trypanosomatids. *Febs j* **2009,** *276* (2), 376-86.
2. Jones, D. C.; Hallyburton, I.; Stojanovski, L.; Read, K. D.; Frearson, J. A.; Fairlamb, A. H., Identification of a k-opioid agonist as a potent and selective lead for drug development against human African trypanosomiasis. *Biochem. Pharmacol.* **2010,** *80*, 1478-1486.
3. Raz, B.; Iten, M.; Grether-Buhler, Y.; Kaminski, R.; Brun, R., The Alamar Blue assay to determine drug sensitivity of African trypanosomes (*T. b. rhodesiense* and *T. b. gambiense*) in vitro. *Acta Tropica* **1997,** *68*, 139-147.
4. Gillingwater, K.; Kunz, C.; Braghiroli, C.; Boykin, D. W.; Tidwell, R. R.; Brun, R., *In vitro, ex vivo*, and *in vivo* activities of diamidines against *Trypanosoma congolense* and *Trypanosoma vivax*. *Antimicrob Agents Chemother* **2017,** *61* (5), e02356-16.
5. https://www.americanpharmaceuticalreview.com/Featured-Articles/160452-Thermodynamic-vs-Kinetic-Solubility-Knowing-Which-is-Which.
6. Baragana, B.; Norcross, N. R.; Wilson, C.; Porzelle, A.; Hallyburton, I.; Grimaldi, R.; Osuna-Cabello, M.; Norval, S.; Riley, J.; Stojanovski, L.; Simeons, F. R.; Wyatt, P. G.; Delves, M. J.; Meister, S.; Duffy, S.; Avery, V. M.; Winzeler, E. A.; Sinden, R. E.; Wittlin, S.; Frearson, J. A.; Gray, D. W.; Fairlamb, A. H.; Waterson, D.; Campbell, S. F.; Willis, P.; Read, K. D.; Gilbert, I. H., Discovery of a quinoline-4-carboxamide derivative with a novel mechanism of action, multistage antimalarial activity, and potent *in vivo* efficacy. *J. Med. Chem.* **2016,** *59* (21), 9672-9685.
7. Gibson, W., The origins of the trypanosome genome strains *Trypanosoma brucei brucei* TREU 927, *T. b. gambiense* DAL 972, *T. vivax* Y486 and *T. congolense* IL3000. *Parasit Vectors* **2012,** *5*, 71.
8. Duffy, C. W.; Morrison, L. J.; Black, A.; Pinchbeck, G. L.; Christley, R. M.; Schoenefeld, A.; Tait, A.; Turner, C. M.; MacLeod, A., *Trypanosoma vivax* displays a clonal population structure. *Int J Parasitol* **2009,** *39* (13), 1475-1483.
9. Frearson, J. A.; Brand, S.; McElroy, S. P.; Cleghorn, L. A.; Smid, O.; Stojanovski, L.; Price, H. P.; Guther, M. L.; Torrie, L. S.; Robinson, D. A.; Hallyburton, I.; Mpamhanga, C. P.; Brannigan, J. A.; Wilkinson, A. J.; Hodgkinson, M.; Hui, R.; Qiu, W.; Raimi, O. G.; van Aalten, D. M.; Brenk, R.; Gilbert, I. H.; Read, K. D.; Fairlamb, A. H.; Ferguson, M. A.; Smith, D. F.; Wyatt, P. G., N-myristoyltransferase inhibitors as new leads to treat sleeping sickness. *Nature* **2010,** *464* (7289), 728-732.
10. Langmead, B.; Salzberg, S. L., Fast gapped-read alignment with Bowtie 2. *Nat Methods* **2012,** *9* (4), 357-9.
11. Li, H.; Handsaker, B.; Wysoker, A.; Fennell, T.; Ruan, J.; Homer, N.; Marth, G.; Abecasis, G.; Durbin, R., The Sequence Alignment/Map format and SAMtools. *Bioinformatics* **2009,** *25* (16), 2078-9.
12. Li, H., A statistical framework for SNP calling, mutation discovery, association mapping and population genetical parameter estimation from sequencing data. *Bioinformatics* **2011,** *27* (21), 2987-93.
13. Carver, T.; Harris, S. R.; Berriman, M.; Parkhill, J.; McQuillan, J. A., Artemis: an integrated platform for visualization and analysis of high-throughput sequence-based experimental data. *Bioinformatics* **2012,** *28* (4), 464-9.
14. Glover, L.; Alsford, S.; Baker, N.; Turner, D. J.; Sanchez-Flores, A.; Hutchinson, S.; Hertz-Fowler, C.; Berriman, M.; Horn, D., Genome-scale RNAi screens for high-throughput phenotyping in bloodstream-form African trypanosomes. *Nature protocols* **2015,** *10* (1), 106-133.
15. Carver, T.; Harris, S. R.; Otto, T. D.; Berriman, M.; Parkhill, J.; McQuillan, J. A., BamView: visualizing and interpretation of next-generation sequencing read alignments. *Brief Bioinform* **2013,** *14* (2), 203-212.

16. Rico, E.; Jeacock, L.; Kovářová, J.; Horn, D., Inducible high-efficiency CRISPR-Cas9-targeted gene editing and precision base editing in African trypanosomes. *Sci Rep* **2018,** *8* (1), 7960.
